# Traditional healers working with primary care and mental health for early intervention in psychosis in young persons: protocol for the feasibility cluster randomised controlled trial

**DOI:** 10.1136/bmjopen-2023-072471

**Published:** 2023-07-14

**Authors:** Saeed Farooq, Saima Sheikh, Lisa Dikomitis, Mian Mukhtar Ul Haq, Abdul Jalil Khan, Noor Sanauddin, Malik Wajid Ali, Johar Ali, Muhammad Firaz Khan, Imran Chaudhry, Nusrat Husain, Muhammad Gul, Muhammad Irfan, Gabrielle Andrews, Prachi Kaistha, Syed Muhammad Uzair Shah, Ishfaq Azeemi, Shumaila Hamid, Aaliya Minhaz, Christian Mallen, Martyn Lewis

**Affiliations:** 1 School of Primary, Community and Social Care, Keele University, Keele, UK; 2 Midlands Partnership NHS Foundation Trust, Stafford, UK; 3 Kent and Medway Medical School, University of Kent, Canterbury, UK; 4 Medical Teaching Institution, Lady Reading Hospital, Peshawar, Pakistan; 5 Institute of Public Health & Social Sciences, Khyber Medical University, Peshawar, Pakistan; 6 Department of Sociology, University of Peshawar, Peshawar, Pakistan; 7 Combined Military Hospital, Rawalpindi, Pakistan; 8 Pakistan Institute of Living and Learning, Karachi, Pakistan; 9 Ziauddin University Hospital, Karachi, Pakistan; 10 Division of Psychology and Mental Health, The University of Manchester, Manchester, UK; 11 Research and Innovation, Midlands Partnership NHS Foundation Trust, Stafford, UK; 12 Department of Mental Health, Psychiatry & Behavioral Sciences, Peshawar Medical College, Peshawar, Pakistan; 13 Riphah International University, Islamabad, Pakistan; 14 School of Medicine, Keele University, Keele, UK; 15 Department of Chemistry, Shaheed Benazir Bhutto Women University, Peshawar, Pakistan

**Keywords:** MENTAL HEALTH, PUBLIC HEALTH, EPIDEMIOLOGIC STUDIES, Child & adolescent psychiatry, MEDICAL EDUCATION & TRAINING, Schizophrenia & psychotic disorders

## Abstract

**Objectives:**

In low/middle-income countries (LMICs), more than half of patients with first-episode psychosis initially seek treatment from traditional and religious healers as their first care. This contributes to an excessively long duration of untreated psychosis (DUP). There is a need for culturally appropriate interventions to involve traditional and spiritual healers to work collaboratively with primary care practitioners and psychiatrists through task-shifting for early detection, referral and treatment of first episode of psychosis.

**Methods:**

To prevent the consequences of long DUP in adolescents in LMICs, we aim to develop and pilot test a culturally appropriate and context-bespoke intervention. *T*raditional *HE*alers working with primary care and mental *H*ealth for early interventi*O*n in *P*sychosis in young p*E*rsons (THE HOPE) will be developed using ethnographic and qualitative methods with traditional healers and caregivers. We will conduct a randomised controlled cluster feasibility trial with a nested qualitative study to assess study recruitment and acceptability of the intervention. Ninety-three union councils in district Peshawar, Pakistan will be randomised and allocated using a 1:1 ratio to either intervention arm (THE HOPE) or enhanced treatment as usual and stratified by urban/rural setting. Data on feasibility outcomes will be collected at baseline and follow-up. Patients, carers, clinicians and policymakers will be interviewed to ascertain their views about the intervention. The decision to proceed to the phase III trial will be based on prespecified stop–go criteria.

**Ethics and dissemination:**

Ethical approval has been obtained from Keele University Ethical Review Panel (ref: MH210177), Khyber Medical University Ethical Review Board (ref: DIR/KMU-EB/IG/001005) and National Bioethics Committee Pakistan (ref no. 4-87/NBC-840/22/621). The results of THE HOPE feasibility trial will be reported in peer-reviewed journals and academic conferences and disseminated to local stakeholders and policymakers.

**Trial registration number:**

ISRCTN75347421.

**STRENGTHS AND LIMITATIONS OF THIS STUDY**

- The proposed study seeks to develop and pilot test a novel and culturally appropriate intervention for early detection, referral and treatment of first episode of psychosis with the aid of traditional spiritual healers to prevent consequences of untreated psychosis in resource-limited settings.
- The present study aims to integrate mental health and primary care services to provide early intervention in untreated psychosis in collaboration with traditional healers and evaluate it in a feasibility randomised controlled trial (RCT).
- We will use a mixed-methods design, comprising both quantitative and qualitative methods, to develop, test and evaluate the feasibility of the intervention and understand its strengths and limitations.
- A limitation of this feasibility cluster RCT is that the planned study has a small study and is not powered to test the effectiveness of the intervention. This being a feasibility trial will evaluate the acceptability of the intervention and will provide precision estimates for an adequately powered definitive RCT in future.

## Introduction

### Background and rationale

First-episode psychosis (FEP) has peak onset between 15 and 35 years of age.^1–3^ FEP can disrupt education, reduce long-term employment, cause huge economic burden and is associated with significantly higher mortality as compared with the general population.^4^ Duration of untreated psychosis (DUP), the period between the onset of psychosis and initiating treatment, is 125 weeks in low/middle-income countries (LMICs) compared with 62.5 weeks in high-income countries (HICs).^5^ Even when initiated, the treatment is mostly limited to acute episodes resulting in a huge treatment gap with around 70% of people not receiving regular care^6 7^ with incidence of self-harm during long periods of untreated psychosis.^5^

A major factor for the long DUP in LMICs is the preference of the majority of the patients to see traditional and spiritual healers (TSHs) as their first care providers for mental disorders.^8^ This is consistent with a systematic review showing predominantly supernatural explanatory models of illness for psychosis in LMICs.^9^ Research with TSHs in Pakistan^10–12^ has identified that the following terms are used for identifying people suffering from psychosis: influence of jinn, saya (influence or shadow), ‘strong spell or under the control of spirits’ lewanay (insane) and ‘superstitious’. This is consistent with previous literature.^11 12^ These healers use different methods of treatment, including de-possession from jinn or saya, instruction and advice regarding prayers, Tawiz (symbolic lines and verses written on a piece of paper), Dam (holy verses recited and blown on the body), Dua (prayers) and special religious ceremonies, which include reciting holy verses loudly and family accompanying the spiritual healers in a trance-like state and exorcism to remove the spirits from home and offering holy water.^11 12^ Other methods for treatment include homoeopathy, danyalism (shamanism), the holy Tabbarak (blessed food), sugar or salt, and drinking Dam Wala Pani (sacred verses recited on water) available at Dargahs (shrines).^12 13^

Another key factor in the long DUP is the lack of primary care involvement in the treatment of psychosis.^14^ The integration of mental health and primary care services is widely advocated, especially in LMICs, and primary care practitioners have an important role in providing effective early intervention in psychosis.^15^ However, the evidence suggests that primary care physicians (PCPs) have practically no role in the treatment of psychosis in LMICs.^14^

Evidence available in other disease areas, such as HIV/AIDS, suggests that with appropriate training and support, services have been scaled up in collaboration with traditional healers.^16^ However, there are hardly any interventions for improving collaboration with TSHs in mental health. A systematic review on the effectiveness of TSHs in treating mental disorders highlighted limited interventions mostly described in generally poor-quality studies with no intervention found involving TSHs in the care of FEP.^17^ There is an urgent need to work collaboratively with TSHs and develop and rigorously evaluate early intervention in psychosis interventions in LMICs, as TSHs have far greater influence in treating mental disorders, and a pilot study showed that TSHs are open to identifying and referring individuals with possible psychosis.^18^

### Aims and objectives

To prevent the consequences of long DUP in young persons in LMICs, we aim to develop and pilot test a culturally appropriate and context-bespoke intervention (Traditional HEalers working with primary care and mental Health for early interventiOn in Psychosis in young pErsons (THE HOPE)) for early identification, referral and management of FEP in the young population in Peshawar, Khyber Pakhtunkhwa (KP) in Pakistan and evaluate its feasibility for implementation. THE HOPE intervention will involve TSHs working collaboratively with primary care practitioners and psychiatrists through task-shifting for early detection, referral and treatment of FEP.

#### Objectives

1. To co-develop with key stakeholders a culturally appropriate intervention for early identification, referral and management of FEP.
2. To investigate the acceptability of task-sharing and training procedures, and to establish pathways for referral and management of FEP in consultation with all stakeholders.
3. To establish the feasibility and acceptability of involving mental health service users in training and feasibility study methods.
4. To evaluate the acceptability and feasibility of THE HOPE in terms of changes in knowledge and referral to mental health services by TSH and users’ satisfaction.
5. To estimate the parameters (eg, recruitment and retention rates), means and standard deviations of the key outcome measures to benchmark potential outcome measures and enable sample size calculations for future pragmatic randomised controlled trials (RCTs).
6. To assess the best methods for implementing and evaluating the intervention in the healthcare system.

## Methods and analysis

The research plan for THE HOPE Study includes a development phase to robustly co-design and co-produce THE HOPE intervention following the Medical Research Council framework for developing and evaluating complex interventions.^19^ This paper describes in detail the methodology of the pragmatic cluster pilot RCT and the intervention development process is given here briefly.

The intervention will be co-developed working with TSHs, PCPs and service users through a series of (1) culturally appropriate interactive community engagement activities, (2) ethnographic studies and (3) qualitative studies. Trained ethnographers will participate in up to 15 different communal settings involving interactions with TSHs, healthcare workers, patients and carers. Ethnographic data will provide insights for intervention development and will inform about disease awareness and indigenous knowledge, stigma, lived experience of the disease in adolescents, effects on autonomy, the concept of psychosis, impact on social relations, local lay referral system, any harmful practices used by TSHs, and triggers, barriers and facilitators to seeking formal healthcare.

The findings of the ethnography will feed into the topic guides for semistructured interviews (SSIs) and focus group discussions (FGDs) that will be undertaken with parents, especially mothers, who have a critical role in initiating psychiatric treatment for their child with FEP. We will conduct SSIs (n=~25) with both patients and carers and six FGDs with 8–12 participants, including TSHs, PCPs, psychiatrists and other health workers. These SSIs and FGDs will be conducted in the local Pashto language to explore perceptions, understandings and experiences with FEP of patients and carers, involvement of young persons in decision-making, ways to improve early detection of psychosis, views regarding the nature of symptoms in young people, experiences of receiving treatment from TSHs, barriers and facilitators to early treatment, training needs for TSHs and professionals and best methods to deliver the intervention.

This development phase will inform the development of THE HOPE intervention (see below), which will be evaluated in a cluster feasibility RCT.

### Study design

We will use a pragmatic cluster pilot RCT to test the feasibility and acceptability of the intervention. The study will be reported using the Consolidated Standards of Reporting Trials extension for randomised pilot and feasibility trials.^20^

### Study setting

The study will be carried out in Peshawar district, the capital city of KP province in Pakistan. District Peshawar has a population of about 4 million. The district has 93 union councils (UCs) as basic administrative divisions. Each UC has, on average, a population of about 20 000 and is served by a primary healthcare centre called Basic Health Unit (BHU), providing essential primary healthcare services. The mental health services are provided mainly in three teaching hospitals in Peshawar with about 20 qualified psychiatrists.

### Randomisation

Randomisation will occur at the UC level and be allocated using a 1:1 ratio to either the intervention arm (THE HOPE) or enhanced treatment as usual (ETAU) and stratified by urban/rural setting.

### Sample size

This being a feasibility study, we do not provide a power calculation as there is no proposal for formal hypothesis testing, and the sample size focuses on the required precision of feasibility parameters in line with recommendations for pilot and feasibility studies.^21^ FEP has a relatively low incidence of about 26.6 per 100 000 person-years.^22^ In order to obtain an adequate sample size to measure feasibility outcomes, we will include all UCs in district Peshawar (n=93) in the sample, allocated to ETAU and THE HOPE arm in a 1:1 ratio. We will aim to recruit a minimum of 90 patients (45 in each arm) over a 12-month period. We believe this will help us to achieve reasonable precision of feasibility estimates, for example, for evaluation of intervention fidelity and participant retention in the study (as a proportion). This will enable the 90% one-sided lower confidence bound to be derived to a margin of error of 0.1 for single-arm evaluation and 0.07 for overall sample evaluation. This fits the criteria for sample size justification and calculation recommendations for pilot and feasibility studies.^23^

### The intervention and control arms

#### The intervention arm

**Figure 1:**
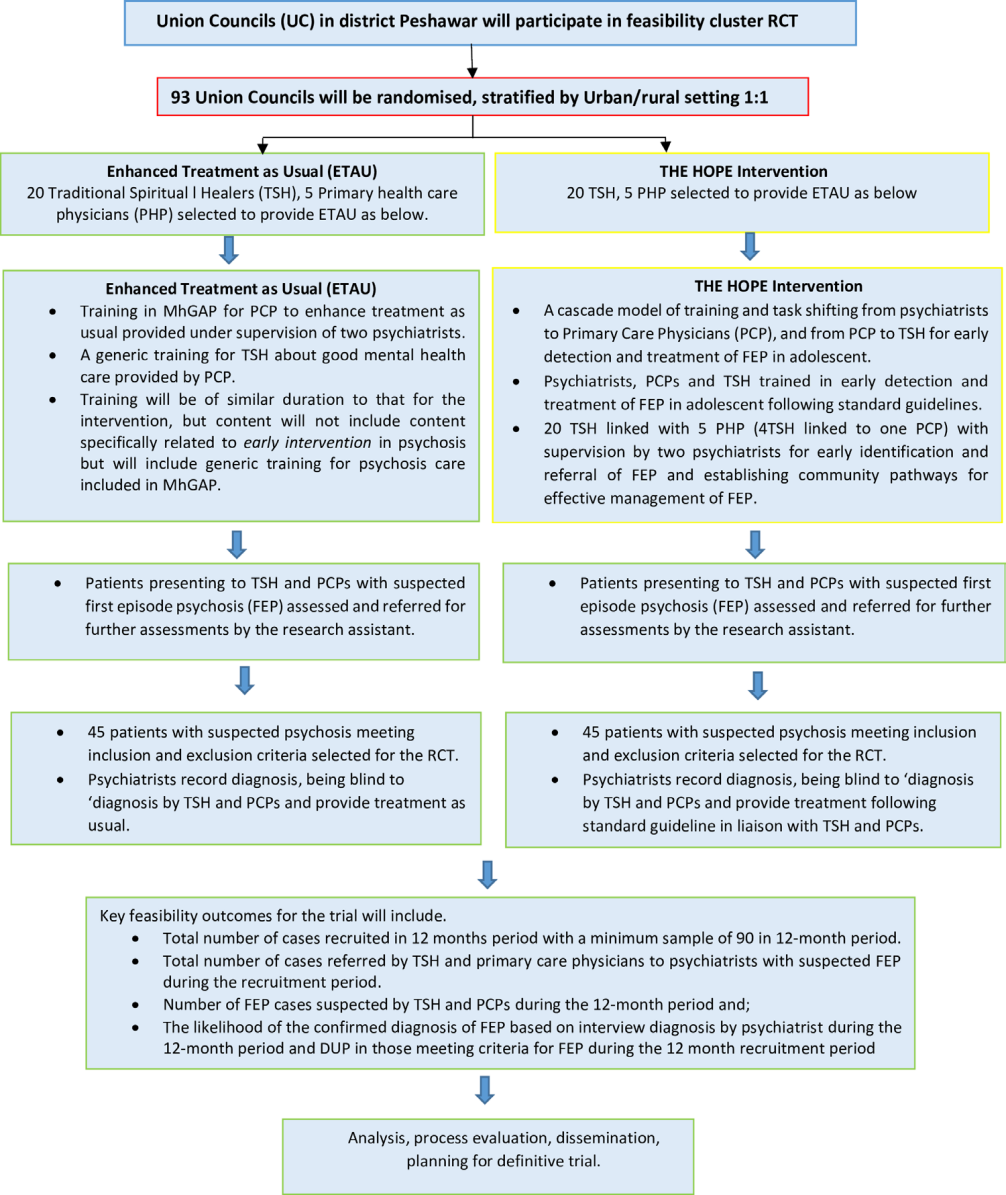
The HOPE flow diagram. DUP, duration of untreated psychosis; MhGAP, Mental Health Gap Action Programme; RCT, randomised controlled trial; THE HOPE, Traditional HEalers working with primary care and mental Health for early interventiOn in Psychosis in young pErsons.

THE HOPE intervention arm will receive THE HOPE intervention in addition to ETAU. THE HOPE is an organisational/service-level intervention with the following essential components (see figure 1):

1. A cascade model of training and task-shifting from psychiatrists to PCPs and from PCPs to TSHs for early detection and treatment of FEP in adolescents.
2. Linking TSHs and PCPs with mental health professionals for early identification and referral of FEP and establishing community pathways for effective management of FEP.

A group of four TSHs will be linked with one PCP working in the nearest BHU. PCPs trained in early identification and referral of FEP in adolescents in Work Package 1 (WP1) will work collaboratively with the TSH and psychiatrist supervising the treatment.

The TSHs and PCPs will work collaboratively in establishing care pathways for the optimum management of FEP in young persons.

The cascade training for early identification and management will consist of the following:

#### Training for psychiatrists

First, a team of psychiatrists with extensive experience in early intervention in psychosis and child and adolescent psychiatry in the UK and Pakistan will train local psychiatrists in the early detection and treatment of FEP. We will train two psychiatrists who will act as master trainers (MTs). These MTs, in turn, will train PCPs. We will use face-to-face teaching, case studies and direct clinical supervision. The training will be based on recent guidelines^24 25^ on the early detection and management of psychosis in young people^24^ adopted for the local settings. Mental Health Gap Action Programme (MhGAP) guidelines on Training of Trainers^26^ will be used for training MTs, and quality assured by Khyber Medical University (KMU).

#### Training for PCPs

Second, these MTs will train PCPs using interactive methods. PCPs in both arms will be trained in the MhGAP treatment guidelines, while those in the intervention arm will receive additional training in the early detection and management of FEP by MTs, as described above.

#### Training for TSHs

Third, these PCPs will train TSHs for identification, referral and working collaboratively with PCPs and health services for effective management of FEP. The information gained from ethnographic and qualitative studies will inform the contents, format and methodology for training TSHs. Based on our experience of working with traditional healers in the STOPS+ Programme,^10^ the training will mainly focus on the following topics: overcoming barriers to working with psychiatric services, symptoms and signs of FEP and differentiating these from normal behaviours expected in adolescence, attitudes and behaviours towards people with psychosis, avoiding harmful practices, respecting individual autonomy, preventing gender-based discrimination and information about referral pathways.

The training will be delivered in a workshop.

Two main educational tools used in workshops will be (1) case vignettes of typical patients for identification of suspected cases of FEP, which we have successfully used in our previous research with lay health workers, and (2) a video in the local language that will deliver the key messages on topics mentioned above.

We have used this model of task-shifting and cascade training in our previous research^27^ and will use a similar approach in implementing the intervention.

#### Control arm: ETAU

This arm will include treatment as usual, enhanced by MhGAP training for PCPs and generic training for TSHs about good mental health. Training will be of similar duration to that for the intervention but will not include content specifically related to early intervention in psychosis but will include generic training for psychosis care included in MhGAP. ETAU provided in the control clusters will include the treatment received by patients’ routine healthcare setting, which includes treatment provided by a psychiatrist in the outpatient clinics of the psychiatry department of the local hospital and brief counselling about the treatment and outcome of the disorder.

A group of four TSHs will be linked with one PCP working in the nearest BHU. The PCPs in the control group will be trained in the general management of psychosis provided in the MhGAP manual.

### Identification of the PCPs and TSHs

We will select 20 TSHs who will be linked with five PCPs working in the local primary healthcare centres in each arm of the study. A group of five TSHs will be linked with one PCP working in the nearest primary healthcare centre. The PCPs will be randomly selected in both intervention and control arms and will be linked to TSHs. The selection will be made by the Department of Family Medicine at KMU.

Our previous work in this area has identified three types of TSH: (1) Pirs (spiritual leaders in Sufi tradition) with well-established shrines, (2) imams, spiritual leaders based in mosques and (3) traditional healers who practise traditional methods such as (taweez) alms or herbs. After consultations with community elders, 20 TSHs in each arm will be selected based on (1) an established reputation for treating mental health problems and not using harmful practices, and (2) willingness to work with the healthcare system.

### Patient and public involvement

THE HOPE comprises a novel culturally appropriate early intervention for FEP that will involve patient and public community engagement at every stage of the research (intervention development, feasibility RCT and process evaluation). Key stakeholders, including service users, care providers, cultural groups, community experts and policymakers, will be consulted at each stage of the research process. Keele patient and public involvement and engagement group and service users’ group in Peshawar, Pakistan provided key inputs in developing the study design.

### Participant eligibility criteria

We will recruit patients (aged 14–25 years) residing in one of the UCs in district Peshawar with first ever psychotic episode who have not received antipsychotic medication previously, or if they already have used antipsychotic medications, it was for no longer than 6 weeks.^28^ The FEP diagnosis will be made at an interview by psychiatrists based on the International Classification of Diseases, 11th revision (ICD-11) criteria for schizophrenia, persistent delusional disorder, acute and transient psychotic disorders, schizoaffective disorder, mood disorders (mania, severe depressive episode, bipolar affective disorder) with psychotic symptoms.

The exclusion criteria will be: (1) evidence of overt learning disability; (2) organic brain damage or pervasive developmental disorder; (3) severe substance abuse (except nicotine dependence); and (4) a young person or parent not willing to provide informed consent.

### Recruitment

Participants suspected of having FEP will be identified from the following possible sources: (1) potentially eligible participants seen by TSHs, (2) potentially eligible participants identified by the PCPs, (3) those presenting in mental health services with suspected FEP and have been in contact and receiving treatment from a TSH in the area.

The cluster allocation will determine the treatment path for each participant. It is possible, for example, that a potentially eligible patient residing in the control cluster presents to the TSH in the intervention arm and vice versa. In such a case, the patient will be given the choice of visiting the nearest TSH based in the control arm (and vice versa). Otherwise, he/she will not be included in the study.

To avoid any selection bias in both groups, the recruitment into the study (both arms) would be done using the same methods by an independent researcher who would enrol in a non-selection bias way that gives parity/equality in uptake. The baseline information about all suspected cases of psychosis by TSHs will be collected, including symptomatology and demographic features. Those with a confirmed diagnosis of psychosis (by the psychiatric team following the procedures given below) will be treated in one of the treatment arms.

**Figure 2:**
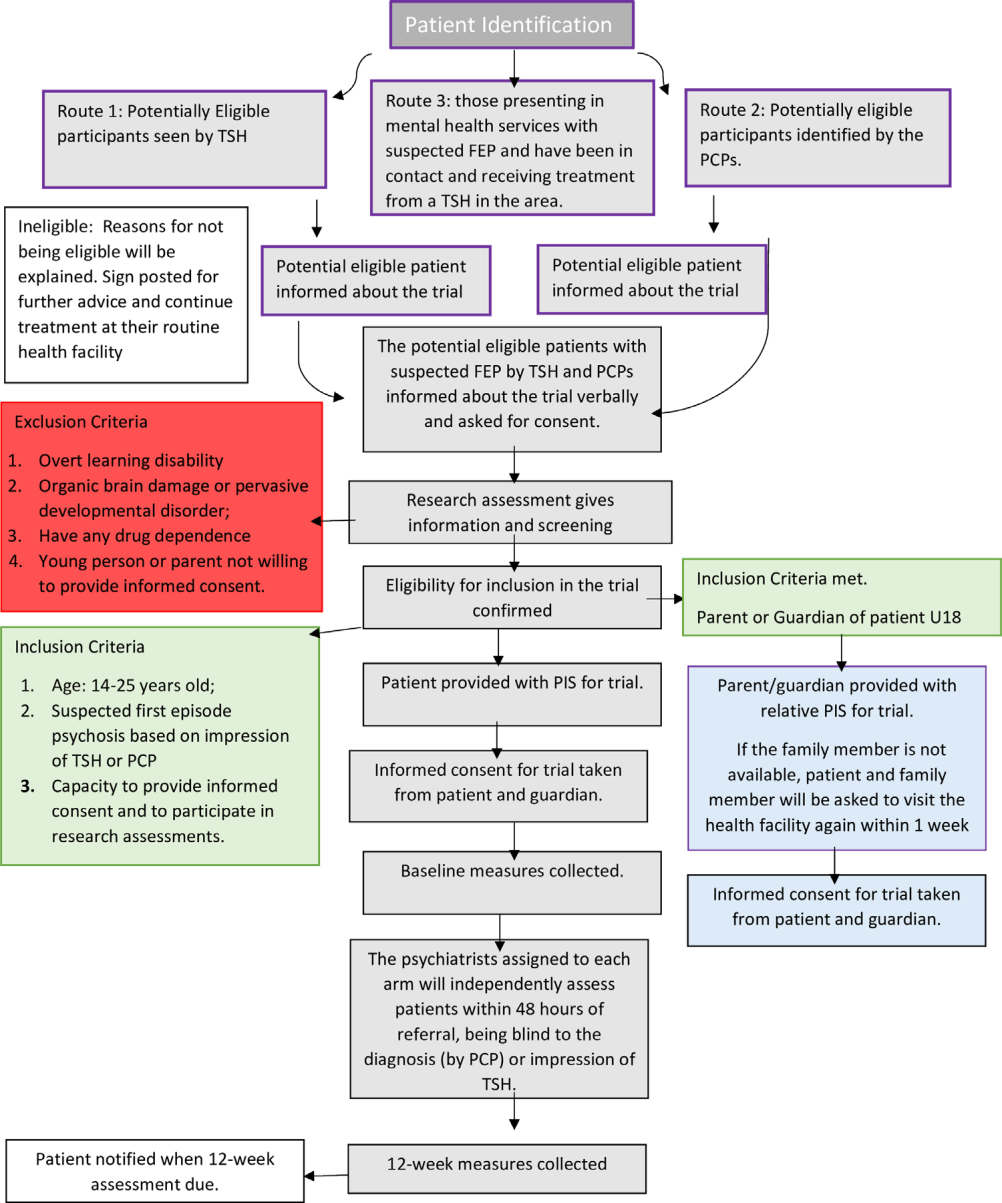
Patient flow and assessments in THE HOPE trial. FEP, first-episode psychosis; PCPs, primary care physicians; PIS, participants’ information sheet; THE HOPE, Traditional HEalers working with primary care and mental Health for early interventiOn in Psychosis in young pErsons; TSH, traditional and spiritual healer.

Potential participants will be verbally informed about the trial by the TSHs, PCPs or research assistant (RA) working with the research team. Patients who agree to participate will be given a full written information sheet about the study by an RA (written information sheets read by RA for those who cannot read) about the study. In case of a young person below the age of 16 years, written informed consent will be obtained from the patient and the parent, who will almost always be accompanying the young person (online supplemental appendices 1 and 2). For illiterate participants, witnessed oral consent and a thumbprint in lieu of a signature will be sufficient. The witness will not be a member of the research team. Both alternatives to full written consent are routinely practised in Pakistan, are culturally acceptable and have been used in our previous studies in this setting^27 29^ (see figure 2).

10.1136/bmjopen-2023-072471.supp1 Supplementary data

Those willing to participate will be requested to complete the baseline sociodemographic data and a suicide risk screen based on the MhGAP intervention guide by an RA. In case of any concerns about the safety of the individual due to suicide risk, the RA will seek help from psychiatric services following the study standard operating procedure developed for this purpose.

### Study procedures and data collection

The RA will refer all suspected cases from TSHs and PCPs to the psychiatrists assigned to each arm, who will independently assess patients within 48 hours of referral, being blind to the diagnosis (by PCPs) or impression of TSHs. The ‘diagnosis’ of FEP by TSHs will be recorded using an instrument developed in consultation with TSHs. This instrument will indicate the likelihood of a person suffering from FEP, as judged by the TSH. Separately, TSHs will also be asked to identify the lay terminology they use in their routine work to capture their conceptualisation of FEP. For patients referred from primary care, PCPs will be asked to provide the likely diagnosis based on ICD-10 (see figure 2).^30^

The psychiatrists will be trained to achieve adequate inter-rater reliability in making the FEP diagnosis. The psychiatrists will diagnose based on the ICD-10 criteria of FEP described above.

Those diagnosed with FEP will be started on treatment in collaboration with participating PCPs or TSHs (if the patients and family want to continue treatment by TSHs) in the intervention arm, while those in the control arm will be provided with the treatment as usual. The treatment will be based on recent guidelines for the treatment of FEP adopted for the purpose of the study.

#### Quantitative data collection and follow-up

Once the participant has provided informed consent, trained RAs will collect the demographic information and complete baseline assessment and suicide risk screen based on the MhGAP intervention guide. Follow-up data will be collected 12 weeks post-baseline by the RAs blinded to the allocation status.

##### Outcome measures

###### Primary outcome measures

Key feasibility outcomes for the trial will include the total number of cases recruited in the 12-month period and cases referred by TSHs and PCPs to psychiatrists with suspected FEP during the recruitment period. It will also include the number of FEP cases suspected by TSHs and PCPs and the likelihood of the confirmed diagnosis of FEP based on interview diagnosis by psychiatrists and DUP in those meeting criteria for FEP.

###### Secondary outcome measures

In addition to feasibility outcomes, the study will aim to measure a range of clinical outcomes and service utilisation measures for assessing the feasibility and acceptability of the intervention. In addition, process evaluation (see below) will be done using qualitative methods. Table 1 provides details of these outcomes and the baseline and follow-up points for these assessments.

**Table 1:** Data collection schedule and list of assessments

Description	Measure	Screening assessment	Baseline assessment	4 weeks post-baseline	3 months post-baseline
Age	Date of birth	✓			
Gender	Male/female	✓			
Education	Years spent in education	✓			
Marital status		✓			
Work status		✓			
Psychopathology	Brief Psychiatric Rating Scale^31^		✓		✓
	Young Mania Rating Scale^32^		✓		✓
	Hamilton Depression Rating Scales^33^		✓		✓
	Clinical Global Impression Scale^34^		✓		✓
Occupational and social functioning	Global Assessment of Functioning^35^		✓		✓
Duration of untreated psychosis	Comprehensive Assessment of At-Risk Mental States^36^	✓			
Quality of life	EuroQol-5^37^		✓		✓
Family burden	Perceived Family Burden Scale^38^		✓		✓
Perceived stigma	Internalised Stigma of Mental Illness^39^		✓		✓
Physical health	Weight and body mass index		✓		✓
Substance abuse	Drug Abuse Screening Tool^40^		✓		✓
Response to antipsychotic treatment	Brief Psychiatric Rating Scale^31^			✓	
Adherence to antipsychotic treatment	Medication Adherence Rating Scale^41^				✓
Rates of disengagement	Response to contact requests for a consecutive 4-week period during follow-up				✓
Remission rates	Score of 3 or below on the Brief Psychiatric Rating Scale,^31^ maintained at least for 3 months		✓		✓
Service utilisation metrics	indicated by: Number of TSHs approached, consented and completed the training programme Patients screened for suspected psychosis Total number of confirmed cases	✓	✓		✓
The acceptability of trial procedures	Adherence to trial procedures and completion of the assessments at baseline and 12 weeks		✓		✓
Fidelity of intervention	First-Episode Psychosis Services Fidelity Scale, modified and adapted for the local settings^43^		✓		✓
Implementation costs	The Client Service Receipt Inventory^44^		✓		✓

TSHs, traditional and spiritual healers.

1. **Psychopathology:** Brief Psychiatric Rating Scale (BPRS),^31^ Young Mania Rating Scale,^32^ Hamilton Depression Rating Scale,^33^ and Clinical Global Impression Scale^34^ for measuring improvement in symptoms and remission rates.
2. **Functioning:** Global Assessment of Functioning.^35^
3. **DUP:** Comprehensive Assessment of At-Risk Mental States at baseline.^36^
4. **Quality of life:** EuroQol-5.^37^
5. **Family burden**: Family Burden Scale.^38^
6. **Stigma**: Internalised Stigma of Mental Illness.^39^
7. **Physical health**: weight and body mass index.
8. **Substance abuse**: Drug Abuse Screening Tool.^40^
9. **Response to antipsychotic treatment:** change in BPRS^31^ from baseline to 4 weeks after starting antipsychotics.
10. **Adherence to antipsychotic treatment:** Medication Adherence Rating Scale.^41^
11. **Rates of disengagement** are defined as a participant’s refusal to engage with treatment or not responding to contact requests for a consecutive 4-week period during follow-up while still residing within the study site catchment area.^42^
12. **Remission rates:** improvement in psychotic symptoms from baseline (operationalised as a score of 3 or below on the BPRS^31^), maintained for 3 months.
13. **Service utilisation metrics** measures will be used as a measure of the acceptability of the intervention. These will include patients screened for suspected psychosis out of all those presenting to at different contact points, the median time taken in screening, frequency and duration of contacts, the median length of time to treatment from assessment to starting treatment, the proportion of cases identified as at-risk mental state for FEP out of total accepted cases and the willingness of TSHs to work with health services (indicated by the number of TSHs approached, consented and completing training programme).
14. **The acceptability of trial procedures** will also be assessed through adherence to trial procedures and completion of the assessments at baseline and 12 weeks.
15. **Fidelity of intervention:** FEP Services Fidelity Scale, modified and adapted for the local settings,^43^ will be used to assess the degree to which mental health teams deliver evidence-based care for FEP.
16. **Implementation costs:** the Client Service Receipt Inventory^44^ will measure direct and indirect costs in implementing the intervention to estimate health economic costs from the payer and provider perspective. We will estimate mean, median and quartile costs at each time point.

##### Blinding and allocation concealment

It is not possible to blind study participants from their treatment arm allocation. However, the assessment team conducting the baseline and follow-up assessments will be blinded to the treatment arm of the cluster. During all assessments, the key feasibility outcome measures will be completed first to minimise the risk of bias in the event of unblinding, and if it occurs, the point of unblinding will be recorded. Sensitivity analyses will be carried out to assess the effect of unblinding on the clinical outcomes. The trial statistician will be blind to allocation status. The psychiatrist diagnosing FEP will be blind to the diagnosis given by PCPs and the impression of illness by TSHs. This will help to minimise the bias and provide a good measure of training intervention.

#### Qualitative data collection

We will use SSIs after the trial follow-up to explore the views of participants and carers from both trial arms, physicians, traditional healers and decision-makers with responsibilities for developing or implementing health policy in the directorate of health for future implementation and evaluation. Around four to six separate interviews will be conducted with each group. The SSIs will explore the (1) acceptability of educational materials and delivery, (2) acceptability of evaluating THE HOPE in a definitive trial, (3) burden of training, (4) barriers and facilitators to early detection and collaboration with mental health services, and (5) overall satisfaction with the intervention. Participants for these interviews will be purposively sampled to stratify by gender and urban setting. An SSI topic guide will be used to facilitate the interviews.

##### Process evaluation

We will conduct (1) interviews with those receiving and delivering THE HOPE (assess acceptability) and (2) interviews with healthcare decision-makers (to support implementation) for the evaluation of training (pretraining and post-training scores).

### Withdrawal criteria

There are no anticipated risks involved for those who decide to take part in this study. The study includes sharing personal experiences, which some may find uncomfortable. Participants are under no obligation to answer any questions that make them feel uncomfortable. The information received from the participant will not be used in any way that might cause any harm. Participants will be informed that if there is any problem throughout the participation, the trained mental health professionals will be available for help.

Participants will be informed that they can withdraw from the study at any time without giving a reason and that their clinical care will not be affected. Participants will be withdrawn from the study in the event of any of the following: (1) non-compliance with the study protocol that results in a serious breach of protocol (Good Clinical Practice or other), which can compromise data integrity or significantly affect the scientific value of the reported results; (2) a serious untoward incident compromising the patient’s safety. Any data collected up to the point of discontinuation from the study will be reported as such.

Patients requiring inpatient admission or electroconvulsive therapy or any other therapeutic procedure as part of the treatment or exacerbation of the symptoms will not be discontinued from the study, provided that the procedure is as advised by their treating psychiatrist and the patient continues to consent for the study.

In case of reported harm to the participants directly because of receiving the treatment or the control, the harms will be reported to the Data and Safety Monitoring Board (DSMB) and Trial Steering Committee (TSC), who will further advise if the trial needs to be stopped.

### Study timeline

We aim for the pilot RCT to start on 1 April 2023. Recruitment will commence when the trial commences and will be conducted until 1 April 2024; there will be a 3-month follow-up after the recruitment of the last patient. We anticipate that the analysis will start in September 2024.

### End of trial

The end of the study is defined as when the 12-week follow-up assessment and all qualitative interviews have been completed. Keele University ethics and the ethics board in the local context will be notified of the end of the study.

### Data management

All data will be managed in line with the protocol and will be stored securely following the KMU and Keele University standard policy on data storage and confidentiality. Individual participant data will be pseudonymised through the use of a unique study ID and will be stored securely and separately from any identifiable data. Access to data will be restricted to members of the Keele and KMU research team and will be stored in accordance with standard operating procedures. Outcome data will be entered into an electronic database which will be stored on an encrypted secure network at KMU, requiring a password to access. Data entry will be quality-checked and coded using standard processes.

### Data analysis

#### Quantitative analysis

Being a feasibility trial, the main analysis will focus on process outcomes and include estimation (with one-sided and two-sided 80% and 90% CI estimation) of the number/proportion of potentially eligible patients, consent rate, overall uptake rate (in relation to number potentially eligible), average cluster sizes (and variation in cluster sizes) and treatment fidelity—the proportion of patients having the protocolled intervention pathway (intervention arm), intervention acceptability, assessment of possible contamination effect across clusters, overall retention/follow-up rate of participants. Our point and interval estimates will be considered alongside target success criteria for informing overall feasibility and potential progress to a main RCT.

We will also calculate means and CIs of clinical outcomes (both within-study and between-study groups) and determine which outcome(s) is/are most sensitive to change, and evaluate parameters required in order to inform the sample size calculation for the main trial.

Due to the lack of similar studies in this area, it is not possible to suggest clear progression criteria to guide the decisions about the feasibility of proceeding to a definitive trial in future. We suggest following a priori progression criteria in order to guide the feasibility of a future definitive trial. In light of the following criteria, the trial findings will be presented to the steering committee, which will advise about the feasibility of proceeding to the definitive trial.

**Red**, that is, the progression to a definitive trial using the proposed intervention and procedures is not feasible. Recruitment below 90 in total (45 in both arms) over a 12-month period, less than 70% of recruited sample completed all follow-up visits, and less than 70% of TSHs in both arms consented, participated in the training and worked with psychiatric services in referring the suspected cases.

**Amber**, that is, the progression to a definitive trial using the proposed intervention is feasible but would require modifications in the intervention, recruitment or follow-up procedures. Recruitment of 90–130 participants in total over a 12-month period, 70%–80% of recruited sample completed all follow-up visits, and 70%–85% of TSHs in both arms consented, participated in training and worked with psychiatric services in referring the suspected cases.

**Green**, that is, the progression to a definitive trial using the proposed intervention is feasible without changes in the intervention, recruitment or follow-up procedures.

Recruitment of more than 130 participants in total over a 12-month period (averaging at least about 1.5 patients recruited per UC), more than 80% of the recruited sample completed all follow-up visits and >85% proportion of TSHs in both arms consented, participated in training and worked with psychiatric services in referring the suspected cases.

#### Qualitative analysis

An inductive, exploratory framework will be adopted using thematic analysis incorporating elements of grounded theory.^45^ A sample of early transcripts will be independently coded by the research team, and a coding framework will be agreed upon. This framework will then be applied to subsequent coding. Independent researchers will analyse coded data to develop categories and themes. The constant comparison^46^ will be used in the analysis to explore connections within and across transcripts and codes, highlighting consistencies and variation. The analysis will be an iterative process; emergent findings will be used to further refine topic guides for subsequent interviews.

### Trial management and monitoring

The Project Management Committee for THE HOPE Study will be responsible for setting up ongoing management and monitoring of the trial. The trial will be monitored in line with the protocol and the trial standard operating procedures. The standard operating procedure will be based on the recently concluded STOPS+ trial,^47^ and the procedures will be audited by an independent team. An independent TSC, which will have service user and sponsor representatives, will monitor the trial progress. The TSC is empowered to independently review the ethical and data management procedures. A DSMB will be convened to ensure the safety of participants and the integrity of the data.

#### Safety and adverse events reporting

The principal investigator and local site staff will be responsible for detecting, documenting and reporting events that meet the definition of adverse events (AEs) or serious AEs (SAEs). Any potential AE or SAE reported by the participant, family member or identified by the trial staff will be reported to the supervising psychiatrist within 24 hours. It will be the responsibility of the supervising psychiatrist to assess the seriousness and causality, depending on the nature of the event reported. The relevant safety data will be reported to the principal investigator and the Local Ethical Advisory and Review Board, according to the nature of the AE and the necessary reporting time frame. AEs categorised as ‘serious’ (SAEs) will also be reported to DSMB and TSC within 15 days, whereas non-serious AEs will be ‘bundled’ and documented in the annual summary sent to the regulatory authority if required.

## Ethics and dissemination

This study has obtained ethical approval from Keele University Ethical Review Panel (ref: MH210177), KMU Ethical Review Board (ref: DIR/KMU-EB/IG/001005) and National Bioethics Committee Pakistan (ref no. 4-87/NBC-840/22/621).

Any amendments to the protocol will not be implemented prior to receipt of the required approvals. This does not affect the individual clinician’s responsibility to take immediate action if thought necessary to protect the health and interest of individual participants. The chief investigator/sponsor will ensure that the main Research Ethics Committee is notified that the trial has finished within 90 days after the end of the trial. If the trial is terminated prematurely, those reports will be made within 15 days after the end of the trial.

Study results will be shared with local communities, service users, traditional healers and health administrators. Throughout the study period, we will hold several information-sharing and dissemination meetings involving different stakeholders to discuss the findings from different phases of the study and trial findings. We will also produce training manuals for healthcare professionals and TSHs, and these will be shared widely with relevant local and international health and policy agencies. The study findings will also be presented in local and international research and health service research. At least three publications are planned, which will be published in open-access resources. Manuscripts with the results of the pilot trial will be submitted for publication in peer-reviewed journals and presented at academic conferences. Trial results will be disseminated to patients with a summary sheet outlining the trial findings in lay language. We will also disseminate findings through social media such as Twitter accounts. Participant-level dataset will be available after trial completion on request for academic use, in line with patient confidentiality and anonymity guidelines.

## Discussion

In many LMICs, psychiatric disorders are considered mainly as a result of a social imbalance or to have spiritual causes, and these beliefs are also prevalent in immigrants living in HICs.^9 48^ These alternate explanations of disease, among other factors, alter the help-seeking behaviour of patients and their pathway to care, which seems to be of great importance in psychotic disorders.^49^ A recent qualitative study highlighted the role of complementary medicine and stigma in DUP in an LMIC setting. It was reported that patients and families had difficulty recognising symptoms as an illness and would attribute these to supernatural causes, resulting in a delay before contacting a general practitioner or psychiatrist. Similarly, a systematic review concluded that a large proportion of patients in LMICs use TSHs as their first point of contact for accessing care associated with longer DUP. The review recommended that services in these countries need to focus on collaborative working with TSHs to facilitate access to biomedical care.^50^

There is almost no literature on implementation research involving traditional healers in the care of psychosis. This study aims to include TSHs and primary care in the treatment pathways for improving the care of FEP. THE HOPE will make a significant contribution to the study of working collaboratively with traditional healers. This will have a significant impact on developing new ways of working with traditional healers and evaluating these using the RCT.

The proposed research has three major innovations: developing intervention by using ethnographic and qualitative methods to understand the context and the working of TSHs; improving the skills of primary care and TSHs by using a well-structured education and training programme; and using a task-sharing and task-shifting approach involving psychiatrists, primary care and traditional healers. We also include a complete process evaluation of the study after the trial is completed.

The results of this study and process evaluation will inform the planning and conduct of a full-scale pragmatic RCT. At present, it is not possible to conduct an adequately powered trial of any intervention with TSHs in these settings, as the feasibility and acceptability of such an intervention have not been studied.

Developing a potential model that can be implemented with the help of traditional healers will have a wide economic and social impact and is likely to be highly acceptable to those using these services. By promoting active partnership between patients, members of the public and researchers, we will maximise impact by recognising and supporting the unique roles service users have in research prioritisation, design and management, data collection and the analysis and dissemination of findings.

The integration of mental health and primary care services is advocated in developed as well as developing countries.^51 52^ The detection and management of FEP in primary care are major challenges and are practically lacking in most LMICs, and evidence suggests the importance of training general practitioners to improve the early detection and management of FEP to reduce the delay between the onset of symptoms and initiation of treatment.^53^ The proposed intervention involves PCPs in training the TSHs. Involving PCPs in The HOPE intervention and cascade model of training will help to involve the primary care in the treatment of FEP and will help to reduce the DUP and build capacity in primary care.

### Limitations of the proposed study

To the best of our knowledge, no study prior to this has attempted collaborating with TSHs to integrate mental health and primary care services to provide early intervention in untreated psychosis and evaluate the model in a feasibility RCT. We use a mixed-methods approach, comprising both quantitative and qualitative methods, to develop, test and evaluate the feasibility of the intervention and understand its strengths and limitations.

A major limitation of our study is the small sample size. As discussed in the Methods section, the sample size is aimed to evaluate the acceptability and feasibility of the intervention, but the study is not adequately powered to test the clinical parameters. Contamination between the intervention and control arm is possible, considering that the participants in the intervention and the control arm may interact within the community. We propose measures to implement the blinding of assessors to the trial arms. However, it may not be possible to maintain the assessors’ blindness to the intervention or control arm.

## Supplementary Material

Reviewer comments

Author's manuscript
