# Supplementary material for: Traditional healers working with primary care and mental health for early intervention in psychosis in young persons: protocol for the feasibility cluster randomised controlled trial
Source: BMJ Open. 2023 Jul 14;13(7):e072471. doi: 10.1136/bmjopen-2023-072471 (PMC10351321; doi:10.1136/bmjopen-2023-072471)
Supplement: Supplementary data [file bmjopen-2023-072471supp001.pdf]

Appendices

Appendix 1. Participant Consent Form – Young Person 16+

Participant Identification Number:

Participant Consent Form – Young Person 16+

Study title: Traditional healers working with primary care and mental health for early intervention in psychosis in adolescent: intervention development and feasibility study

This form should be initialled and signed unless the young person is unable to read and write. If they cannot write then the form should be read aloud to the young person and they should be asked to mark the form in the relevant places.

Please initial/mark box if you agree with the statements

1. I confirm that I have read and understood the information sheet dated 05/Mar/2021 (Version 1.0) for the above study. I have had the opportunity to consider the information, ask questions and have had these answered satisfactorily. ☐

2. I understand that participating in the THEHOPE study involves: ☐
   - Attending appointments with the research team. This includes; research assistants, primary care physicians, spiritual healers and psychiatrists.
   - Attending appointments with clinicians where my mental health will be assessed
   - Receiving treatment for my mental health condition if it is appropriate

3. I understand that my participation is voluntary and that I am free to withdraw at any time without giving any reason and without my legal or medical rights being affected. ☐

4. I understand that all information and data collected during the course of the research will be kept strictly confidential and that any personal data e.g. name and address will only be used for the THEHOPE study.

5. I understand that my data will be pseudonymised through the use of a unique study code.

6. I agree that if the researcher is worried about my safety they may have to tell my doctor or other clinician about things I have told them.

Please continue to next page

7. I understand that I may be invited to take part in research relating to THEHOPE in the future.

8. I voluntarily agree to take part in this study.

Name of Participant	Date	Signature or mark
Name of Researcher taking consent	Date	Signature

1 copy for participant and 1 copy for researcher.

Appendix 2. Participant Consent Form – Parent or Carer

Participant Identification Number:

Participant Consent Form –
Parent or Carer

Study title: Traditional healers working with primary care and mental health for early intervention in psychosis in adolescent: intervention development and feasibility study

This consent form is intended for the parents or caregivers of the young person participating in the study.
This form should be initialled and signed unless the parent or carer is unable to read and write.

If they cannot write then the form should be read aloud and they should be asked to mark the form in the relevant places. This consent procedure must be witnessed by a trusted member of the family or community who is independent to the project. It is necessary that this individual then signs the form at the bottom in the indicated place.

Please initial/mark box if you agree with the statements

9. I confirm that I have read and understood the information sheet dated 05/Mar/2021 (Version 1.0) for the above study. I have had the opportunity to consider the information, ask questions and have had these answered satisfactorily. ☐ ☐

10. I understand that participation of **my child** / **young person I care for** in the THEHOPE study involves:
    - **My child** / **young person I care for** attending appointments with the research team. This includes; research assistants, primary care physicians, spiritual healers and psychiatrists.

- Attending appointments with clinicians where **my child** / **young person I care for** will be assessed for their mental health condition
- **My child** / **young person I care for** receiving treatment for their mental health condition if it is appropriate

11. I understand that participation of **my child** / **young person I care for** is voluntary and that we are free to withdraw at any time without giving any reason and without our legal or medical rights being affected. The information I have provided up to that point will be included for research purposes unless I state otherwise. After publication, my information cannot be withdrawn. ☐

Please continue to next page

12. I understand that all information and data collected during the course of the research will be kept strictly confidential and that any personal data e.g. name and address will only be used for the THEHOPE study. ☐
13. I understand that data collected from **my child** / **young person I care for** will be pseudonymised through the use of a unique study code. ☐
14. I understand and agree that should there be any concern for the safety and welfare of **my child/young person I care for** that the research team may wish to contact their doctor or other clinician. ☐
15. I understand that **my child** / **young person I care for** may be invited to take part in research relating to THEHOPE in the future. ☐
16. I voluntarily agree for **my child** / **young person I care for** to take part in this study. ☐

______________________________	___________________________	__________________
Name of Parent / Carer	Date	Signature or Mark
______________________________	___________________________	__________________
Name of Researcher	Date	Signature

taking consent

If parent or carer cannot write:

______________________	___________________	_____________
Name and role of trusted observer	Date	Signature

1 copy for participant and 1 copy for researcher.
