# Supplementary material for: Traditional healers working with primary care and mental health for early intervention in psychosis in young persons: protocol for the feasibility cluster randomised controlled trial
Source: BMJ Open. 2023 Jul 14;13(7):e072471. doi: 10.1136/bmjopen-2023-072471 (PMC10351321; doi:10.1136/bmjopen-2023-072471)
Supplement: Author's manuscript [file bmjopen-2023-072471.draft_revisions.pdf]

BMJ Open

BMJ Open is committed to open peer review. As part of this commitment we make the peer review history of every article we publish publicly available.

When an article is published we post the peer reviewers' comments and the authors' responses online. We also post the versions of the paper that were used during peer review. These are the versions that the peer review comments apply to.

The versions of the paper that follow are the versions that were submitted during the peer review process. They are not the versions of record or the final published versions. They should not be cited or distributed as the published version of this manuscript.

BMJ Open is an open access journal and the full, final, typeset and author-corrected version of record of the manuscript is available on our site with no access controls, subscription charges or pay-per-view fees (http://bmjopen.bmj.com).

If you have any questions on BMJ Open's open peer review process please email info.bmjopen@bmj.com

BMJ Open

Traditional healers working with primary care and mental health for early intervention in psychosis in young persons: Protocol for the feasibility cluster randomised controlled trial

Journal:	*BMJ Open*
Manuscript ID	bmjopen-2023-072471
Article Type:	Protocol
Date Submitted by the Author:	06-Feb-2023
Complete List of Authors:	Farooq, Saeed; Keele University, School of Primary, Community and Social Care<br>Sheikh, Saima; Keele University, School of Primary, Community and Social Care<br>Dikomitis, Lisa; University of Kent, Kent and Medway Medical School<br>Ul-Haq, Mian; Lady Reading Hospital, Medical Teaching Institution<br>Khan, Abdul Jalil; Khyber Medical University, Institute of Public Health & Social Sciences<br>Sanauddin, Noor; University of Peshawar, Department of Sociology<br>Malik, Wajid; Combined Military Hospital<br>Ali, Johar; University of Peshawar, Department of Sociology<br>Khan, Muhammad Firaz; Lady Reading Hospital, Medical Teaching Institution<br>Chaudhry, Imran; Pakistan Institute of Living and Learning<br>Husain, Nusrat; The University of Manchester, Division of Psychology and mental Health<br>Gul, Muhammad; Midlands Partnership NHS Foundation Trust, Research and Innovation<br>Irfan, Muhammad; Peshawar Medical College<br>Andrews, Gabrielle; Midlands Partnership NHS Foundation Trust, Research and Innovation<br>Kaistha, Prachi; Keele University, School of Medicine<br>Shah, Syed Muhammad Uzair; University of Peshawar, Department of Sociology<br>Azeemi, Ishfaq; University of Peshawar, Department of Sociology<br>Hamid, Shumaila; Khyber Medical University, Institute of Public Health & Social Sciences<br>Minhaz, Aaliya; Shaheed Benazir Bhutto Women University<br>Mallen, Christian; Keele University, School of Primary, Community and Social Care<br>Lewis, Martyn; Keele University, School of Primary, Community and Social Care
Keywords:	MENTAL HEALTH, PUBLIC HEALTH, EPIDEMIOLOGIC STUDIES, Child & adolescent psychiatry < PSYCHIATRY, MEDICAL EDUCATION & TRAINING, Schizophrenia & psychotic disorders < PSYCHIATRY

Traditional healers working with primary care and mental health for early intervention in psychosis in young persons: Protocol for the feasibility cluster randomised controlled trial

Saeed Farooq [1, 2,] Saima Sheikh[1], Lisa Dikomitis[3], Mian Mukhtar- Ul- Haq Azeemi [4], Abdul Jalil Khan [5,] Noor Sanauddin[6], Malik Wajid Ali [7], Johar Ali [6] Muhammad Firaz Khan [4], Imran Chaudhry [8, 9], Nusrat Hussain [9,10], Muhammad Gul [2], Muhammad Irfan [11], Gabrielle Andrews [1,2], Prachi Kaista [1], Syed Mohammad Uzair Shah [6], Ishfaq Azeemi [6], Shumaila Hamid [5], Aaliya Minhaz [12], Christian Mallen [1], Martyn Lewis [1]

1. School of Primary, Community and Social Care, Keele University, Keele, Staffordshire, UK
2. Research and Innovation, Midlands Partnership Foundation Trust, Staffordshire, UK
3. Kent and Medway Medical School, University of Kent, Kent, UK
4. Medical Teaching Institution, Lady Reading Hospital, Peshawar, Khyber Pakhtunkhwa, Pakistan
5. Institute of Public Health & Social Sciences, Khyber Medical University, Peshawar, Khyber Pakhtunkhwa, Pakistan
6. Department of Sociology, University of Peshawar, Peshawar, Khyber Pakhtunkhwa, Pakistan
7. Consultant psychiatrist Combined Military Hospital, Rawalpindi
8. Pakistan Institute of Living and Learning, Karachi, Pakistan
9. Division of Psychology and mental Health, University of Manchester, Manchester, UK
10. Research and Innovation, Merseycare NHS Foundation Trust, Liverpool, UK
11. Peshawar Medical College, Peshawar, Khyber Pakhtunkhwa, Pakistan
12. Shaheed Benazir Bhutto Women University, Peshawar, Khyber Pakhtunkhwa, Pak

Correspondence to Dr Saeed Farooq; s.farooq@keele.ac.uk

Professor of Psychiatry and Public Mental Health

School of Medicine, Keele University, Staffordshire, ST5 5BG, UK.

Honorary Consultant Psychiatrist,

Midlands Partnership NHS Foundation Trust.

Version 3. Dated 06.02.2023.

For peer review only - http://bmjopen.bmj.com/site/about/guidelines.xhtml

For peer review only

For peer review only - http://bmjopen.bmj.com/site/about/guidelines.xhtml

	Name	Contact	Postal Address
1	Professor Saeed Farooq	s.farooq@keele.ac.uk	School of Primary, Community and Social Care, Keele University, Keele, Staffordshire, UK, ST5 5BG
2	Saima Sheikh	s.sheikh@keele.ac.uk	School of Primary, Community and Social Care, Keele University, Keele, Staffordshire, UK, ST5 5BG
3	Professor Lisa Dikomitis	lisa.dikomitis@kmms.ac.uk	Kent and Medway Medical School, University of Kent, Kent, UK CT2 7FS
4	Dr Mian Mukhtar -Ul- Haq	doctormian@yahoo.co.uk	Medical Teaching Institution, Lady Reading Hospital, Peshawar, Khyber Pakhtunkhwa, 25000 Pakistan
5	Dr Abdul Jalil Khan	jalilkhan@kmu.edu.pk	Institute of Public Health & Social Sciences, Khyber Medical University, Peshawar, Khyber Pakhtunkhwa,25100, Pakistan
6	Dr Noor Sanauddin	noor.sanauddin@uop.edu.pk	Department of Sociology, University of Peshawar, Peshawar, Khyber Pakhtunkhwa, 25120, Pakistan
7	Dr Malik Wajid Ali	malikwajid10@gmail.com	Consultant psychiatrist Combined Military Hospital, Rawalpindi
8	Professor Johar Ali	johar_ali55@yahoo.com	Department of Sociology, University of Peshawar, Peshawar, Khyber Pakhtunkhwa, 25120, Pakistan
9	Muhammad Firaz Khan	drfiraz786@yahoo.co.uk	Medical Teaching Institution, Lady Reading Hospital, Peshawar, Khyber Pakhtunkhwa, 25000, Pakistan
10	Professor Imran Chaudhry	Imran.chaudhry@pill.ac.uk	Pakistan Institute of Living and Learning, Karachi, Sindh 75600, Pakistan
11	Professor Nusrat Husain	Nusrat.husain@manchester.ac.uk	Division of Psychology and mental Health, University of Manchester, Manchester, UK M13 9PL
12	Dr Muhammad Gul	Muhammad.Gul@mpft.nhs.uk	Research and Innovation, Midlands Partnership Foundation Trust, Staffordshire, UK, ST16 3SR
13	Professor Muhammad	mirfan78@yahoo.com	Peshawar Medical College, Peshawar, Khyber Pakhtunkhwa, Pakistan

For peer review only - http://bmjopen.bmj.com/site/about/guidelines.xhtml

	Irfan		
14	Gabrielle Andrews	gabrielle.andrews@mpft.nhs.uk	Research and Innovation, Midlands Partnership Foundation Trust, Staffordshire, UK
15	Prachi Kaistha	p.k.kaistha@keele.ac.uk	School of Primary, Community and Social Care, Keele University, Keele, Staffordshire, UK, ST5 5BG
16	Syed Muhammad Uzair Shah	uzairshah2011@yahoo.com	Department of Sociology, University of Peshawar, Peshawar, Khyber Pakhtunkhwa, 25120, Pakistan
17	Ishfaq Azeemi	ishfaqazeemi3@gmail.com	Department of Sociology, University of Peshawar, Peshawar, Khyber Pakhtunkhwa, 25120, Pakistan
18	Shumaila Hamid	drshumailahk@gmail.com	Institute of Public Health & Social Sciences, Khyber Medical University, Peshawar, Khyber Pakhtunkhwa,25100, Pakistan
19	Aliya Minhas	aaliyachem@sbbwu.edu.pk	Shaheed Benazir Bhutto Women University, Peshawar, Khyber Pakhtunkhwa, Pakistan
20	Professor Christian Mallena	c.d.mallen@keele.ac.uk	School of Primary, Community and Social Care, Keele University, Keele, Staffordshire, UK, ST5 5BG
21	Dr Martyn Lewis	a.m.lewis@keele.ac.uk	School of Primary, Community and Social Care, Keele University, Keele, Staffordshire, UK, ST5 5BG

For peer review only

Abstract

Objectives: **I**n Low- and Middle-Income Countries (LMICS), more than half of patients with First Episode Psychosis initially seek treatment from traditional and religious healers as their first care. This contributes to an excessively long duration of untreated Psychosis (DUP). There is a need for culturally appropriate interventions to involve traditional and spiritual healers (TSH) to work collaboratively with primary care practitioners (PCPs) and psychiatrists through task shifting for early detection, referral, and treatment of First Episode of Psychosis (FEP).

Methods: To prevent the consequences of long DUP in adolescents in LMICS, we aim to develop and pilot test a culturally appropriate and context-bespoke intervention. **T**raditional **H**Ealers working with primary care and mental **H**ealth for early interventi**O**n in **P**sychosis in young p**Er**sons (THE HOPE) will be developed by using ethnographic and qualitative methods with traditional healers and caregivers. We will conduct a randomised controlled cluster feasibility trial with a nested qualitative study to assess study recruitment and acceptability of the intervention. Ninety-three union councils in district Peshawar, Pakistan, will be randomised and allocated using a 1:1 ratio to either intervention arm (THE HOPE) or Enhanced Treatment as Usual (ETAU) and stratified by urban/rural setting. Data on feasibility outcomes will be collected at baseline and follow-up. Patients, carers, clinicians, and policymakers will be interviewed to ascertain their views about the intervention. The decision to proceed to the phase III trial will be based on prespecified stop-go criteria.

Ethics and dissemination: Ethical approval has been obtained from Keele University Ethical Review Panel (ref: MH210177), Khyber Medical University Ethical Review Board (ref: DIR/KMU-EB/IG/001005) and National Bioethics Committee Pakistan (Ref: No.4-87/NBC-840/22/621). The results of THE HOPE feasibility trial will be reported in peer-reviewed journals and academic conferences and disseminated to local stakeholders and policymakers.

Trial registration***:*** ISRCTN75347421 https://doi.org/10.1186/ISRCTN75347421

Word count: 6175

For peer review only

For peer review only - http://bmjopen.bmj.com/site/about/guidelines.xhtml

Strengths and Limitations:

- The proposed study seeks to develop and pilot test a novel and culturally appropriate intervention for early detection, referral, and treatment of First Episode of Psychosis with the aid of traditional spiritual healers to prevent consequences of untreated psychosis in resource-limited settings.
- The present study aims to integrate mental health and primary care services to provide early intervention in untreated psychosis in collaboration with traditional healers and evaluate it in feasibility randomised controlled trial.
- We will use a mixed methods design, comprising both quantitative and qualitative methods to develop, test and evaluate the feasibility of the intervention, understand its strengths and limitations.
- A limitation of this feasibility cluster RCT is that the planned study has a small study and is not powered to test the effectiveness of the intervention. This being a feasibility trial will evaluate the acceptability of the intervention and will provide precision estimates for an adequately powered definitive randomised controlled trial in future.

Introduction:

Background and Rationale:

First Episode Psychosis (FEP) has peak onset between 15-35 years of age.[1,2,3] FEP can disrupt education, reduce long-term employment, causes huge economic burden and is associated with significantly higher mortality as compared to the general population.[4] Duration of Untreated Psychosis (DUP), the period between the onset of psychosis and initiating treatment is 125 weeks in low-and middle-income countries (LMICs) compared to 62.5 weeks in high-income countries (HICs).[5] Even when initiated, the treatment is mostly limited to acute episodes resulting in a huge treatment gap with around 70% of people not receiving regular care[6,7] with incidence of self-harm during long periods of untreated psychosis.[5]

A major factor for the long DUP in LMICs is the preference of the majority of the patients to see traditional and spiritual healers (TSHs) as their first care providers for mental disorders.[8] This is consistent with a systematic review showing predominantly supernatural explanatory models of illness for psychosis in LMICs.[9] Research with TSHs in Pakistan[10,11,12] has identified that the following terms are used for identifying people suffering from psychosis: influence of jinn, saya (influence or shadow), 'strong spell or under the control of spirits' lewanay (insane) and 'superstitious'. This is consistent with previous literature.[11,12] These healers use different methods of treatment,

For peer review only - http://bmjopen.bmj.com/site/about/guidelines.xhtml

including de-possession from jinn or saya, instruction and advice regarding prayers, Tawiz (symbolic lines and verses written on a piece of paper), Dam (Holy verses recited and blown on the body), Dua (prayers), and special religious ceremonies which includes reciting holy verses loudly and family accompanying the spiritual healers in a trance like state and exorcism to remove the spirits from home and offering holy water.[11,12] Other methods for treatment include homoeopathy, danyalism (shamanism), the holy Tabbarak (blessed food), sugar or salt, drinking Dam Wala Pani (sacred verses recited on water) available at Dargahs (shrines).[12, 13]

Another key factor in the long DUP is the lack of primary care involvement in the treatment of psychosis.[14] The integration of mental health and primary care services is widely advocated, especially in LMIC, and primary care practitioners have an important role in providing effective early intervention in psychosis.[15] However, the evidence suggests that primary care physicians have practically no role in the treatment of psychosis in LMIC.[14]

Evidence available in other disease areas, such as HIV/AIDS suggests that with appropriate training and support, services have been scaled up in collaboration with traditional healers.[16] However, there are hardly any interventions for improving collaboration with TSH in mental health. A systematic review on the effectiveness of TSHs in treating mental disorders highlighted limited interventions mostly described in generally poor-quality studies with no intervention found on involving TSHs in care of FEP.[17] There is an urgent need to work collaboratively with TSH and develop and rigorously evaluate EIP interventions in LMICs as TSHs have far greater influence in treating mental disorders, and a pilot study showed that TSH are open to identifying and referring individuals with possible psychosis.[18]

Aims and Objectives:

To prevent the consequences of long DUP in young person in LMICS, we aim to develop and pilot test a culturally appropriate and context-bespoke intervention (THE HOPE) for early identification, referral, and management of FEP in the young population in Peshawar, Khyber Pakhtunkhwa (KP) in Pakistan and evaluate its feasibility for implementation. THE HOPE intervention will involve TSHs working collaboratively with primary care practitioners (PCPs) and psychiatrists through task shifting for early detection, referral, and treatment of FEP.

Objectives:

1. To co-develop with key stakeholders a culturally appropriate intervention for early identification, referral, and management of FEP.
2. To investigate acceptability of task sharing, training procedures and to establish pathways for referral and management of FEP in consultation with all stakeholders.
3. To establish feasibility and acceptability of involving mental health service users in training and feasibility study methods.

For peer review only - http://bmjopen.bmj.com/site/about/guidelines.xhtml

4. To evaluate the acceptability and feasibility of the THEHOPE in terms of changes in knowledge and referral to mental health services by TSH and users' satisfaction.
5. To estimate the parameters (e.g., recruitment and retention rates), means and standard deviations of the key outcome measures to benchmark potential outcome measures and enable sample size calculations for future pragmatic RCT.
6. To assess the best methods for implementing and evaluating the intervention in the health care system.

Methods and analysis:

The research plan for THE HOPE study includes a development phase to robustly co-design and co-produce the THEHOPE intervention following the MRC framework for developing and evaluating complex interventions.[19] This paper describes in detail the methodology of the pragmatic cluster pilot RCT and intervention development process is given here briefly.

The intervention will be co-developed working with TSH, PCP and service users through a series of (i) culturally appropriate interactive community engagement activities, (ii) ethnographic studies and (iii) qualitative studies. Trained ethnographers will participate in up to 15 different communal settings involving interactions with TSH, healthcare workers, patients and carers. Ethnographic data will provide insights for intervention development and will inform about: disease awareness and indigenous knowledge, stigma, lived experience of the disease in adolescent, effects on autonomy, concept of psychosis, impact on social relations, local lay referral system, any harmful practices used by TSH, and triggers, barriers and facilitators to seeking formal healthcare.

The findings of the ethnography will feed into the topic guides for Semi-Structured Interviews (SSI) and Focus Group Discussions (FGD) that will be undertaken with parents, especially mothers, who have a critical role in initiating psychiatric treatment for their child with FEP. We will conduct SSI (n=~25) with both patients and carers) and six FGDs, with 8-12 participants including TSH, primary care physicians, psychiatrists, and other health workers. These SSIs and FGDs will be conducted in the local Pashto language, to explore perceptions, understandings and experience with FEP of patients and carers, involvement of young persons in decision making, ways to improve early detection of psychosis, views regarding the nature of symptoms in young person, experience of receiving treatment from TSH, barriers and facilitators to early treatment, training needs for TSH and professionals and best methods to deliver the intervention.

This development phase will inform the development of THE HOPE intervention (see below), which will be evaluated in a cluster feasibility RCT.

Study Design:

We will use a pragmatic cluster pilot RCT to test the feasibility and acceptability of the intervention. The study will be reported using the CONSORT extension for randomised pilot and feasibility trials.[20]

For peer review only - http://bmjopen.bmj.com/site/about/guidelines.xhtml

Study Setting:

The study will be carried out in the district Peshawar, the capital city of KP province in Pakistan. District Peshawar has a population of about 4 million. The district has 93 union councils (UC), as basic administrative division. Each UC has on average a population of about 20000 and served by a primary health care centre called Basic Health Unit (BHU) providing essential primary health care services. The mental health services are provided mainly in 3 teaching hospitals in Peshawar with about 20 qualified psychiatrists.

Randomisation:

Randomisation will occur at the Union Council level and will be allocated using a 1:1 ratio to either intervention arm (THE HOPE) or Enhanced Treatment as Usual (ETAU) and stratified by urban/rural setting.

Sample Size:

This being a feasibility study we do not provide a power calculation as there is no proposal for formal hypothesis testing and the sample size focuses on required precision of feasibility parameters in line with recommendations for pilot and feasibility studies.[21] First Episode Psychosis has relatively low incidence of about 26·6 per 100 000 person-years.[22] In order to obtain adequate sample size to measure feasibility outcomes we will include all union councils in district Peshawar (n=93) in the sample; allocated to ETAU and THE HOPE arm in 1:1 ratio. We will aim to recruit a minimum of 90 patients (45 in each arm) over 12 months period. We believe this will help us to achieve reasonable precision of feasibility estimates e.g., for evaluation of intervention fidelity and participant retention in the study (as a proportion). This will enable the 90% one-sided lower confidence bound to be derived to a margin of error of 0.1 for single-arm evaluation and 0.07 for overall sample evaluation. This fits with criteria for sample size justification and calculation recommendations for pilot and feasibility studies.[23]

The intervention and control arms: (see Figure 1)

The Intervention Arm

THE HOPE intervention arm will receive THE HOPE intervention in addition to Enhanced Treatment As Usual (ETAU). THE HOPE is an organisational/service level intervention with following essential components:

1. **A cascade model** of training and task shifting from psychiatrists to Primary Care Physicians (PCP), and from PCP to TSH for early detection and treatment of FEP in adolescent.

For peer review only - http://bmjopen.bmj.com/site/about/guidelines.xhtml
For peer review only

2. **Linking TSH and PCP** with mental health professionals for early identification and referral of FEP and establishing community pathways for effective management of FEP

A group of 4 TSH will be linked with one PCP working in the nearest Basic Health Unit (BHU). The PCPs who are trained in early identification and referral of FEP in adolescent in WP1 will work collaboratively the TSH and psychiatrist supervising the treatment.

. The TSH and PCPs will work collaboratively in establishing care pathways for optimum management of FEP in young persons.

The cascade training for early identification and management will consist of following:

Training for Psychiatrists:

First, a team of psychiatrists with extensive experience in early intervention in psychosis and child and adolescent psychiatry in UK and Pakistan will train local psychiatrists in early detection and treatment of FEP. We will train two psychiatrists who will act as Master Trainers (MTs). These master trainers in turn will train PCPs. We will use face to face teaching, case studies and direct clinical supervision. The training will be based on recent guidelines[24,25] on early detection and management of psychosis in young people [24] adopted for the local settings. MhGAP guidelines on Training of Trainers[26] will be used for training master trainers and quality assured by Khyber Medical University (KMU) University.

Training for Primary Care Physicians (PCPs):

Secondly, these MTs will train PCPs using interactive methods. PCPs in both arms will be trained in the mhGAP treatment guidelines while those in intervention arm will receive additional training in early detection and management of FEP by MTs as described above.

Training for Traditional and Spiritual Healers (TSH):

Thirdly, these PCPs will train TSH for identification, referral and working collaboratively with PCPS and health services for effective management of FEP. The information gained from ethnographic and qualitative studies will inform the contents, format and methodology for training TSH. Based on our experience of working with traditional healers in STOPS+ programme[10] the training will mainly focus on following topics: overcoming barriers in working with psychiatric services, symptoms and signs of FEP and differentiating these from normal behaviours expected in adolescence, attitudes and behaviours towards people with psychosis, avoiding harmful practices, respecting individual autonomy, preventing gender-based discrimination and information about referral pathways.

The training will be delivered in a workshop.

Two main educational tools used in workshops will be (i) case vignettes of typical patients for identification of suspected cases of FEP which we have successfully used in our previous research with lay health workers and (ii) a video in local language that will deliver the key messages on topics mentioned above.

For peer review only - http://bmjopen.bmj.com/site/about/guidelines.xhtml

We have used this model of task shifting and cascade training in our previous research[27] and will use similar approach in implementing the intervention.

<u>Control arm: Enhanced Treatment as Usual (ETAU):</u>

This arm will include treatment as usual, enhanced by mhGAP training for PCPs and a generic training for TSH about good mental health. Training will be of similar duration to that for the intervention but will not include content specifically related to early intervention in psychosis but will include generic training for psychosis care included in mhGAP. ETAU provided in the control clusters will include the treatment received by patients' routine healthcare setting, which includes treatment provided by a psychiatrist in the outpatient clinics of the psychiatry department of the local hospital and brief counselling about the treatment and outcome of the disorder.
A group of four TSH will be linked with one PCP working in the nearest Basic Health Unit (BHU). The PCPs in control group will be trained in general management of psychosis provided in MhGAP manual.

Identification of the PCPs and TSHs:

We will select 20 TSH who will be linked with 5 primary care physicians (PCPs) working the local primary health care centres in each arm of the study. A group of five TSH will be linked with one PCP working in the nearest PHC. The PCPs will be randomly selected in both intervention and control arms and will be linked to TSHs. The selection will be done by the department of family medicine at Khyber Medical University.

Our previous work in this area has identified three types of TSH: (i) Pirs (spiritual leaders in Sufi tradition) with well-established shrines, (ii) imams, spiritual leaders based in mosques and (iii) traditional healers who practice traditional methods such as (taweez) alms or herbs. After consultations with community elders, 20 TSH in each arm will be selected based on (i) an established reputation for treating mental health problems and not using harmful practises and (ii) willingness to work with health care system.

Patient and Public Involvement

HOPE comprises a novel culturally appropriate early intervention for FEP that will involve patient and public community engagement at every stage of the research (intervention development, feasibility RCT and process evaluation). Key stakeholders including service users, care providers, cultural groups, community experts and policy makers will be consulted at each stage of the research process. Keele Patient and Public Involvement and Engagement (PPIE) group and service users' group in Peshawar, Pakistan provided key inputs in the development of study design.

For peer review only - http://bmjopen.bmj.com/site/about/guidelines.xhtml

Participants' Eligibility Criteria:

We will recruit patients (aged 14—25 years) residing in one of the union councils in district Peshawar with (a) First ever psychotic episode who have not received antipsychotic medication previously, or if they already have used antipsychotic medications, it was for no longer than six weeks.[28] The FEP diagnosis will be made at interview by psychiatrists using a Composite International Diagnostic Interview based on the International Classification of Diseases, 11th revision (ICD-11) criteria for schizophrenia, persistent delusional disorder, acute and transient psychotic disorders, schizoaffective disorder, mood disorders (mania, severe depressive episode, bipolar affective disorder) with psychotic symptoms.

The exclusion criteria will be (i) evidence of overt learning disability (ii) organic brain damage or pervasive developmental disorder; (iii) severe substance abuse (except nicotine dependence) and young person or parent not willing to provide informed consent.

Recruitment:

Participants suspected of having FEP will be identified from following possible sources (1) potentially eligible participants seen by TSH, (2) potentially eligible participants identified by the PCPs (3) those presenting in mental health services with suspected FEP and have been in contact and receiving treatment from a TSH in the area.

The cluster allocation will determine treatment path for each participant. It is possible, for example, a potentially eligible patient residing in the control cluster presents to the TSH in intervention arm and vice versa. In such a case the patient will be given the choice of visiting the nearest TSH based in the control arm (and vice versa). Otherwise, he/she will not be included in the study.

To avoid any selection bias in both groups, the recruitment into the study (both arms) would be done using the same methods by an independent researcher who would enrol in a non-selection-bias way that gives parity/equality in uptake. The baseline information about all suspected cases of psychosis by TSH will be collected, including symptomatology and demographic features. Those with confirmed diagnosis of psychosis (by the psychiatric team following the procedures given below) will be treated in one of the treatment arms.

Potential participants will be verbally informed about the trial by the TSH, PCPs or RA working with the research team. Patients who agree to participate will be given full written information sheet about the study by a research assistant (RA) (written information sheets read by RA for those who can't read) about the study. In case of young person below the age of 16, written informed consent will be obtained from patient and the parent, who will almost always be accompanying the young persons (Appendix 1 and 2). For participants who are illiterate, witnessed oral consent and a thumb print in lieu of a signature will be sufficient. The witness will not be a member of the research

For peer review only - http://bmjopen.bmj.com/site/about/guidelines.xhtml

team. Both alternatives to full written consent are routinely practiced in Pakistan, are culturally acceptable and have been used in our previous studies in this setting [27, 29] Please see Figure 2.

Those willing to participate will be requested to complete the baseline sociodemographic data and a suicide risk screen based on MhGAP intervention guide by a research assistant (RA). In case of any concerns about safety of the individual due to suicide risk, the RA will seek help from psychiatric services following the study SOP developed for this purpose.

Study procedures and data collection (see Figure 2):

The RA will refer all suspected cases from TSH and PCP to the psychiatrists assigned to each arm who will independently assess patients within 48 hours of referral, being blind to the diagnosis (by PCP) or impression of TSH. The 'diagnosis' of FEP by TSH will be recorded using an instrument developed in consultation with TSH. This instrument will indicate the likelihood of a person suffering from FEP, as judged by the TSH. Separately, TSH will also be asked to identify the lay terminology they use in their routine work to capture their conceptualisation of FEP. For patients referred from primary care, PCPs will be asked to provide the likely diagnosis based on ICD-10.[30]

The Psychiatrists will be trained for achieving adequate interrater reliability in making the FEP diagnosis. The psychiatrists will make a diagnosis based on ICD-10 criteria of first episode psychosis as described above.

Those diagnosed with FEP will be started on treatment in collaboration with participating PCPs or TSH (if the patients and family want to continue treatment by TSH) in the intervention arm, while those in the control arm will be provided the treatment as usual. The treatment will be based on recent guidelines for the treatment of FEP adopted for the purpose of the study.

Quantitative Data Collection and Follow up:

Once the participant has provided informed consent, trained RAs will collect the demographic information and complete baseline assessment and suicide risk screen based on MhGAP intervention guide. Follow up data will be collected at 12 weeks post baseline by the RAs blinded to the allocation status.

For peer review only - http://bmjopen.bmj.com/site/about/guidelines.xhtml

Outcome Measures

Primary outcome measures: Key feasibility outcomes for the trial will include total number of cases recruited in 12 months period and cases referred by TSH and primary care physicians to psychiatrists with suspected FEP during the recruitment period. It will also include number of FEP cases suspected by TSH and PCPs and the likelihood of the confirmed diagnosis of FEP based on interview diagnosis by psychiatrist and DUP in those meeting criteria for FEP.

Secondary outcome measures: In addition to feasibility outcomes, the study will aim to measure a range of clinical outcomes and service utilisation measures for assessing the feasibility and acceptability of the intervention. In addition,_process evaluation (see below) will be done using qualitative methods. Table 1 provides details of these outcomes and the baseline and follow-up points for these assessments.

For peer review only

For peer review only - http://bmjopen.bmj.com/site/about/guidelines.xhtml

Table 1. Data Collection Schedule and List of Assessments

Description	Measure	Screening Assessment	Baseline Assessment	4 weeks post baseline	3 Month Post baseline
Age	Date of birth	✓			
Gender	Male/female	✓			
Education	Years spent in education	✓			
Marital status		✓			
Work status		✓			
Psychopathology	Brief Psychiatric Rating Scale (BPRS)[31]		✓		✓
	Young Mania Rating Scale (YMRS)[32]		✓		✓
	Hamilton Depression Rating Scales (HDRS)[33]		✓		✓
	Clinical Global Impression Scale (CGI)[34]		✓		✓
Occupational and Social functioning	Global Assessment of Functioning (GAF)[35]		✓		✓
Duration of untreated psychosis	Comprehensive Assessment of At-Risk Mental States (CAARMS)[36]	✓			

For peer review only - http://bmjopen.bmj.com/site/about/guidelines.xhtml

Quality of Life	EuroQol-5[37]		✓		✓
Family Burden	Perceived Family Burden Scale (PFBS)[38]		✓		✓
Perceived Stigma:	Internalized Stigma of Mental Illness (ISMI)[39]		✓		✓
Physical Health	Weight, and Body Mass Index		✓		✓
Substance Abuse	Drug Abuse Screening Tool (DAST-10)[40]		✓		✓
Response to antipsychotics treatment	Brief Psychiatric Rating Scale (BPRS)[31]			✓	
Adherence to antipsychotic treatment	Medication Adherence Rating Scale (MARS)[41]				✓
Rates of disengagement	Response to contact requests for a consecutive 4-week period during follow-up				✓
Remission rates	score of 3 or below on the Brief Psychiatric Rating Scale[31]) maintained at least for three months.		✓		✓
Service utilisation metrics	indicated by;<br>- number of TSH approached, consented and completing training programme.<br>- patients screened for suspected psychosis<br>-Total number of confirmed cases	✓	✓		✓
The acceptability of trial	adherence to trial procedures and completion		✓		✓

For peer review only

For peer review only - http://bmjopen.bmj.com/site/about/guidelines.xhtml

procedures	of the assessments at baseline and 12 weeks.				
Fidelity of intervention:	First-Episode psychosis services fidelity scale, modified and adapted for the local settings[43]		✓		✓
Implementation costs:	The client Service Receipt Inventory[44]		✓		✓

1. **Psychopathology:** Brief Psychiatric Rating Scale (BPRS)[31], Young Mania Rating Scale (YMRS)[32,] Hamilton Depression Rating Scales (HDRS)[33], Clinical Global Impression Scale (CGI)[34] for measuring improvement in symptoms and remission rates
2. **Functioning:** Global assessment of functioning GAF[35]
3. **Duration of Untreated Psychosis:** Comprehensive Assessment of At-Risk Mental States (CAARMS) at baseline[36]
4. **Quality of life:** EuroQol-5[37]
5. **Family burden**: Family Burden Scale[38]
6. **Stigma**: Internalised Stigma of Mental Illness[39]
7. **Physical health**: weight, and Body Mass Index
8. **Substance abuse**: Drug Abuse Screening Tool[40]
9. **Response to antipsychotics treatment:** Change in BPRS[31] from baseline to four weeks after starting antipsychotics.
10. **Adherence to antipsychotic treatment:** Medication Adherence Rating Scale (MARS)[41]
11. **Rates of disengagement** defined as a participant's refusal to engage with treatment or not responding to contact requests for a consecutive 4-week period during follow-up while still residing within the study site catchment area.[42]
12. **Remission rates:** Improvement in psychotic symptoms from baseline (operationalised as a score of 3 or below on the Brief Psychiatric Rating Scale[31]) maintained for three months.

For peer review only - http://bmjopen.bmj.com/site/about/guidelines.xhtml

13. **Service utilisation metrics** measures will be used as measure of acceptability of intervention. These will include patients screened for suspected psychosis out of all those presenting to at different contact points, the median time taken in screening, frequency and duration of contacts, median length of time to treatment from assessment to starting treatment and proportion of cases identified as At-Risk Mental State (ARMS) for FEP out of total accepted cases, the willingness of TSH to work with health services (indicated by number of TSH approached, consented and completing training programme)
14. **The acceptability of trial procedures** will also be assessed through adherence to trial procedures and completion of the assessments at baseline and 12 weeks.
15. **Fidelity of intervention:** First-Episode psychosis services fidelity scale, modified and adapted for the local settings[43] will be used to assess the degree to which mental health teams deliver evidence-based care for FEP.
16. **Implementation costs:** The client Service Receipt Inventory[44] will measure direct and indirect costs in the implementing the intervention to estimate health economic costs from the payer and provider perspective. We will estimate mean, median and quartile costs at each time point.

For peer review only - http://bmjopen.bmj.com/site/about/guidelines.xhtml

Blinding and allocation concealment:

It is not possible to blind study participants from their treatment arm allocation. However, the assessment team that conducts the baseline and follow up assessments will be masked to the treatment arm of the cluster. During all assessments, the key feasibility outcome measures will be completed first to minimise the risk of bias in the event of unmasking and, if it occurs, the point of unmasking will be recorded. Sensitivity analyses will be carried out to assess the effect of unmasking on the clinical outcomes. Trial statistician will be blind to allocation status. The psychiatrist making the diagnosis of FEP will be blind to diagnosis given by PCPs and impression of illness by TSH. This will help to minimise the bias and provide good measure of training intervention.

Qualitative Data Collection:

We will use semi structured interviews after the trial follow up to explore the views of participants and carers from both trial arms, physicians, traditional healers, and decision-makers with responsibilities for developing or implementing health policy in the directorate of health for future implementation and evaluation.   Around 4-6 separate interviews will be conducted with each group. The semi structured interviews will explore the (1) acceptability of educational materials and delivery, (2) the acceptability of evaluating THEHOPE in definitive trial (3) the burden of training (4) barriers and facilitators to early detection and collaboration with mental health services and (5) overall satisfaction with the intervention. Participants for these interviews will be purposively sampled to stratify by gender and urban setting. A semi-structured interview topic guide will be used to facilitate the interviews.

Process evaluation:

We will conduct (i) Interviews with those receiving and delivering THEHOPE (assess acceptability) (ii) Interviews with healthcare decision-makers (to support implementation) evaluation of training (pre- and post-training scores).

Withdrawal criteria:

For peer review only - http://bmjopen.bmj.com/site/about/guidelines.xhtml

There are no anticipated risks involved for those who decide to take part in this study. The study includes sharing personal experiences, which some may find uncomfortable. Participants are under no obligation to answer any questions that make them feel uncomfortable. The information received from the participant will not be used in any way that might cause any harm. Participants will be informed that if there is any problem throughout participation the trained mental health professionals will be available for help.

Participants will be informed that they can withdraw from the study at any time, without giving a reason and that their clinical care will not be affected. Participants will be withdrawn from the study in the event of any of the following (i) Non-compliance with the study protocol that results in serious breach of protocol (GCP or other) which can compromise data integrity or significantly affect the scientific value of the reported results. (ii) A serious untoward incident compromising the patient safety. Any data collected up to the point of discontinuation from study will be reported as such. Patient requiring inpatients in patient admission or ECT (electroconvulsive therapy) or any other therapeutic procedure as part of the treatment or exacerbation of the symptoms will not be discontinued from study, provided that the procedure is as advised by their treating psychiatrist and patient continues to consent for the study.

In case of reported harms to the participants as directly because of receiving the treatment or the control, the harms will be reported to the Data and Safety Monitoring Board (DSMB) and Trial Steering Committee (TSC) who will further advice if the trial needs to be stopped.

Study timeline:

We aim the pilot RCT to start on 1st April 2023. Recruitment will commence when the trial commences and will be conducted till 1st April 2024; there will be a three month follow up after recruitment of the last patient. We anticipate that the analysis will start in September 2024.

End of Trial:

The end of the study is defined as when the 12-week follow-up assessment, and all qualitative interviews have been completed. Keele University ethics and the ethics board in the local context will be notified of the end of the study.

For peer review only - http://bmjopen.bmj.com/site/about/guidelines.xhtml

Data Management:

All data will be managed in line with the protocol and will be stored securely following the KMU and Keele University standard policy on data storage and confidentiality. Individual participant data will be pseudonymised through the use of a unique study ID and will be stored securely and separately from any identifiable data. Access to data will be restricted to members of the Keele and KMU Research Team and will be stored in accordance with SOPs. Outcome data will be entered into an electronic database which will be stored on an encrypted secure network at KMU requiring a password to access. Data entry will be quality checked and coded using standard processes.

Data Analysis:

Quantitative analysis:

Being a feasibility trial, main analysis will focus on process outcomes and include estimation (with one- and two-sided 80% and 90% confidence interval estimation) of number/proportion of potentially eligible patients, consent rate, overall uptake rate (in relation to number potentially eligible), average cluster sizes (and variation in cluster sizes), treatment fidelity – proportion of patients having the protocolled intervention pathway (intervention arm), intervention acceptability, assessment of possible contamination effect across clusters, overall retention/follow-up rate of participants. Our point and interval estimates will be considered alongside target success criteria for informing overall feasibility and potential progress to a main RCT.

We will also calculate means and confidence intervals of clinical outcomes (both within- and between- study groups) and determine which outcome(s) is/are most sensitive to change, and evaluate parameters required in order to inform the sample size calculation for the main trial.

In view of lack of similar studies in this area, it is not possible to suggest clear progression criteria to guide the decisions about the feasibility to proceed to a definitive trial in future. We suggest following a priori progression criteria in order to guide the feasibility of a future definitive trial. The trial findings in the light of following criteria will be presented to the trial steering committee who will advise about the feasibility of proceeding to the definitive trial.

For peer review only - http://bmjopen.bmj.com/site/about/guidelines.xhtml

Red: i.e., the progression to definitive trial using the proposed intervention and procedures is not feasible. Recruitment below 90 in total (45 in both arms) over 12 months period, less than 70% of recruited sample complete all follow up visits and less than 70% of TSH in both arms consented, participated in trainings, and worked with psychiatric services in referring the suspected cases.

Amber i.e., the progression to definitive trial using the proposed intervention is feasible but would require modifications in the intervention, recruitment or follow up procedures. Recruitment of 90-130 participants in total over 12 months period, 70-80 % of recruited sample complete all follow up visits and 70-85% of TSH in both arms consented, participated in trainings, and worked with psychiatric services in referring the suspected cases.

Green i.e. the progression to definitive trial using the proposed intervention is feasible without changes in the intervention, recruitment or follow up procedures.
Recruitment of more than 130 participants in total over 12 months period (averaging at least about 1.5 patients recruited per UC), more than 80% of the recruited sample complete all follow up visits and >85% proportion of TSH in both arms consented, participated in trainings and worked with psychiatric services in referring the suspected cases.

Qualitative analysis:

An inductive, exploratory framework will be adopted using thematic analysis incorporating elements of grounded theory.[45] A sample of early transcripts will be independently coded by the research team and a coding framework agreed upon. This framework will then be applied to subsequent coding. Coded data will be analysed by independent researchers to develop categories and themes. The constant comparison[46] will be used in the analysis to explore connections within and across transcripts and codes, highlighting consistencies and variation. Analysis will be an iterative process, with emergent findings used to further refine topic guides for subsequent interviews.

Trial management and Monitoring: The Project Management Committee for the HOPE study will be responsible for setting up, ongoing management and monitoring of the trial. The trial will be monitored in line with the protocol and the trial standard operating procedures. The standard operating procedure will be based on recently concluded STOPS+ trial[47] and the procedures will be audited by an independent team. An independent TSC which will have service user and sponsor representatives will monitor trial progress. The TSC is empowered to independently review the

For peer review only - http://bmjopen.bmj.com/site/about/guidelines.xhtml

ethical and data management procedures. A DSMB will be convened to ensure the safety of participants and the integrity of the data.

Safety and Adverse events reporting:

The principal investigator and local site staff will be responsible for detecting, documenting, and reporting events that meet the definition of adverse events AE or serious adverse event SAE. Any potential AE or SAE reported by the participant, family member or identified by the trial staff will be reported to the supervising psychiatrist within 24 hours. It will be the responsibility of the supervising psychiatrist to assess the seriousness and causality, depending on the nature of the event reported. The relevant safety data will be reported to the principal Investigator and the Local Ethical Advisory and Review Board, according to the nature of the AE and the necessary reporting timeframe. AEs categorised as "serious" (SAE) will also be reported to Data Safety and Management Board (DSMB) and TSC within 15 days whereas non-serious AEs will be 'bundled' and documented in the annual summary sent to the regulatory authority, if required.

Ethics and Dissemination:

This study has obtained ethical approval from Keele University Ethical Review Panel (ref: MH210177) Khyber Medical University Ethical Review Board (ref: DIR/KMU-EB/IG/001005) and National Bioethics committee Pakistan (Ref: No.4-87/NBC-840/22/621).

Any amendments to the protocol will not be implemented prior to receipt of the required approvals. This does not affect the individual clinician's responsibility to take immediate action if thought necessary to protect the health and interest of individual participants. The Chief Investigator/Sponsor will ensure that the main REC is notified that the trial has finished within 90 days after the end of the trial. If the trial is terminated prematurely, those reports will be made within 15 days after the end of the trial.

Study results will be shared with local communities, service users, traditional healers and health administrators. Throughout the study period, we will hold several information sharing and dissemination meetings involving different stakeholders to discuss the findings from different phases of study and trial findings. We will also produce training manuals used for health care professionals and TSH and these will be shared widely with relevant local and international health and policy agencies. The findings of the study will also be presented in local and international research and

For peer review only - http://bmjopen.bmj.com/site/about/guidelines.xhtml

health-service research. At least three publications are planned which will be published in open access resources. Manuscripts with the results of the pilot trial will be submitted for publication in peer-reviewed journals and presented at academic conferences. Trial results will be disseminated to patients with a summary sheet that will outline the trial findings in lay language. We will also disseminate findings through social media such Twitter accounts. Participant level dataset will be available after trial completion on request for academic use, in line with patient confidentiality and anonymity guidelines.

Discussion:

In many LMIC the psychiatric disorders are considered mainly as a result of social imbalance or to have spiritual causes and these beliefs are also prevalent in immigrant living in high income countries. [48, 9] These alternate explanations of disease, among other factors, alter help-seeking behaviour of patients and their pathway to care, which seems to be of great importance in psychotic disorders.[49] A recent qualitative study highlighted the role alternative medicine and stigma in DUP in an LMIC setting. It was reported that patients and families had difficulty recognising symptoms as an illness and would attribute these to supernatural causes, resulting in a delay before contacting a GP or psychiatrist. Similarly, a systematic review concluded that a large proportion of patients in LMIC use TSH as their first point of contact for accessing care which as associated with longer DUP. The review recommended that services in these countries need to focus collaborative working with TSH to facilitate access to biomedical care.[50]

There is almost no literature on implementation research involving traditional healers in the care of psychosis. This study aims to include the TSH and primary care in the treatment pathways for improving the care of FEP. THE HOPE will make a significant contribution to the study of working collaboratively with traditional healers. This will have significant impact on developing new ways of working with traditional healers and evaluating these using the randomised controlled trial.
The proposed research has three major innovations: developing intervention by using ethnographic and qualitative methods to understand the context and the working of TSH; improving the skills of primary care and TSH by using a well-structured education and training programme and using a task sharing and task shifting approach involving psychiatrists, primary care and traditional healers. We also include a complete process evaluation of the study after the trial is completed.

For peer review only - http://bmjopen.bmj.com/site/about/guidelines.xhtml

The results of this study and process evaluation will inform the planning and conduct of a full-scale pragmatic RCT**.** At present it is not possible to conduct an adequately powered trial of any intervention with TSH in these setting as the feasibility and acceptability of such an intervention is not studied.

Developing a potential model that can be implemented with the help of traditional healers will have wide economic and social impact and is likely to be highly acceptable to those using these services. By promoting the active partnership between patients, members of the public and researchers we will maximise impact by recognising and supporting the unique roles service users have in research prioritisation, design and management, data collection and the analysis and dissemination of findings.

The integration of mental health and primary care services is advocated in developed as well as developing countries[51,52]. The detection and management of FEP in primary care is a major challenge and is practically lacking in most LMIC and evidence suggests the importance of training GPs to improve early detection and management of FEP to reduce the delay between onset of symptoms and initiation of treatment[53].The proposed intervention involves primary care physicians in training the TSH. Involving PCPs in The HOPE intervention and cascade model of training will help to involve the primary care in treatment of FEP and will help to reduce the duration of untreated psychosis build capacity in primary care.

For peer review only - http://bmjopen.bmj.com/site/about/guidelines.xhtml

Footnotes

Acknowledgements: The author would like to thank Dr TK, Keele University for providing valuable suggestions regarding qualitative methods. We also want to thank the service user representative Mr Sher Dil Khan for his valuable contribution to the design and oversight of the study as part of the Trial Steering Committee. We will also like express our gratitude to Keele Patient and Public Involvement and Engagement (PPIE) group and service users' group in Peshawar, Pakistan for their useful suggestions in the study design.

Author Contributions: SF, SS, PK, ML and GA were involved in initial drafts, proofing, and finalising the manuscript. All authors contributed to the conceptualisation and the design of the study. SF, LD, MM, AK, MA, JA, MK, IC, NH, MG, MI, AM, CM, ML obtained the funding for the study, conceived, and developed the intervention and study design, and led creation of the team. LD contributed to design and led qualitative studies for intervention development and process evaluation. GA and MI contributed to writing ethics applications and obtaining the ethics approvals. ML contributed significantly to the study design, analysis plan and economic evaluation part of the protocol. The initial and final manuscript has been read, revised, and approved by all named authors and that there are no other persons who satisfied the criteria for authorship but are not listed.

Funding Statement: This study is funded by Medical Research Council (MRC) UK grant number (MR/T040378/1). The funder has no role in designing and reporting of the study. An independent Trial Steering Committee (TSC) which will have service user and sponsor representatives will monitor trial progress.

Conflict of Interest: None declared.

For peer review only - http://bmjopen.bmj.com/site/about/guidelines.xhtml

For peer review only

References:

1. Kelleher I, Connor D, Clarke MC, Devlin N, Harley M, Cannon M. Prevalence of psychotic symptoms in childhood and adolescence: a systematic review and meta-analysis of population-based studies. Psychological medicine. 2012 Sep;42(9):1857-63. doi:10.1017/S0033291711002960

2. Gerstenberg M, Theodoridou A, Traber-Walker N, Franscini M, Wotruba D, Metzler S, Müller M, Dvorsky D, Correll CU, Walitza S, Rössler W. Adolescents and adults at clinical high-risk for psychosis: age-related differences in attenuated positive symptoms syndrome prevalence and entanglement with basic symptoms. Psychological medicine. 2016 Apr;46(5):1069-78. doi:10.1017/S0033291715002627

3. Sullivan SA, Kounali D, Cannon M, David AS, Fletcher PC, Holmans P, Jones H, Jones PB, Linden DE, Lewis G, Owen MJ. A population-based cohort study examining the incidence and impact of psychotic experiences from childhood to adulthood, and prediction of psychotic disorder. American Journal of Psychiatry. 2020 Apr 1;177(4):308-17. doi: 10.1176/appi.ajp.2019.19060654

4. Farooq S, Large M, Nielssen O, Waheed W. The relationship between the duration of untreated psychosis and outcome in low-and-middle income countries: a systematic review and meta-analysis. Schizophrenia research. 2009 Apr 1;109(1-3):15-23. doi: 10.1016/j.schres.2009.01.008

5. Lora A, Kohn R, Levav I, McBain R, Morris J, Saxena S. Service availability and utilisation and treatment gap for schizophrenic disorders: a survey in 50 low-and middle-income countries. Bulletin of the World Health Organization. 2012 Jan;90(1):47-54B. doi:10.2471/BLT.11.089284

6. De Silva MJ, Lee L, Fuhr DC, Rathod S, Chisholm D, Schellenberg J, Patel V. Estimating the coverage of mental health programmes: a systematic review. International journal of epidemiology. 2014 Apr 1;43(2):341-53. doi:10.1093/ije/dyt191

7. Healy C, Brannigan R, Dooley N, Coughlan H, Clarke M, Kelleher I, Cannon M. Childhood and adolescent psychotic experiences and risk of mental disorder: a systematic review and meta-analysis. Psychological Medicine. 2019 Jul;49(10):1589-99. doi:10.1017/S0033291719000485

8. Burns JK, Tomita A. Traditional and religious healers in the pathway to care for people with mental disorders in Africa: a systematic review and meta-analysis. Social psychiatry and psychiatric epidemiology. 2015 Jun;50(6):867-77. doi:10.1007/s00127-014-0989-7

9. Bhikha AG, Farooq S, Chaudhry N, Husain N. A systematic review of explanatory models of illness for psychosis in developing countries. International Review of Psychiatry. 2012 Oct 1;24(5):450-62. doi:10.3109/09540261.2012.711746

10. Khattak MI, Dikomitis L, Khan MF, Haq MU, Saeed U, Awan NR, Haq ZU, Shepherd T, Mallen CD, Farooq S. Patients' and healthcare professionals' perspectives on a community-based intervention for schizophrenia in Pakistan: A focus group study. Plos one. 2022 Aug 29;17(8):e0273286. doi:10.1371/journal.pone.0273286

11. Saeed K, Gater R, Hussain A, Mubbashar M. The prevalence, classification and treatment of mental disorders among attenders of native faith healers in rural Pakistan. Social psychiatry and psychiatric epidemiology. 2000 Oct;35(10):480-5.

For peer review only - http://bmjopen.bmj.com/site/about/guidelines.xhtml

12. Qidwai W. Use of services of spiritual healers among patients presenting to family physicians at a teaching hospital in Karachi, Pakistan. Pakistan Journal of Medical Sciences. 2003;19(1):52.

13. Mubbashar MH, Saeed K. Development of mental health services in Pakistan. EMHJ-Eastern Mediterranean Health Journal, 7 (3), 392-396, 2001. 2001.

14. Irfan M, Caldas de Almeida JM, Irfan UM, Raza UA, Farooq S. Schizophrenia diagnosis and treatment by general practitioners: A cross-sectional study in district Peshawar, Pakistan. JPMA J Pak Med Assoc. 2015 Sep 1;65(9):937-42.

15. McDaid D, Park AL, Iemmi V, Adelaja B, Knapp M. Growth in the use of early intervention for psychosis services: An opportunity to promote recovery amid concerns on health care sustainability.

16. Homsy J, King R, Balaba D, Kabatesi D. Traditional health practitioners are key to scaling up comprehensive care for HIV/AIDS in sub-Saharan Africa. Aids. 2004 Aug 20;18(12):1723-5. doi: 10.1097/01.aids.0000131380.30479.16

17. Nortje G, Oladeji B, Gureje O, Seedat S. Effectiveness of traditional healers in treating mental disorders: a systematic review. The Lancet Psychiatry. 2016 Feb 1;3(2):154-70. doi:10.1016/S2215-0366(15)00515-5

18. Veling, W., Burns, J. K., Makhathini, E. M., Mtshemla, S., Nene, S., Shabalala, S., ... & Susser, E. (2019). Identification of patients with recent-onset psychosis in KwaZulu Natal, South Africa: a pilot study with traditional health practitioners and diagnostic instruments. Social psychiatry and psychiatric epidemiology, 54(3), 303-312. doi:10.1007/s00127-018-1623-x

19. Skivington K, Matthews L, Simpson SA, Craig P, Baird J, Blazeby JM, Boyd KA, Craig N, French DP, McIntosh E, Petticrew M. A new framework for developing and evaluating complex interventions: update of Medical Research Council guidance. bmj. 2021 Sep 30;374. doi:10.1136/bmj.n2061

20. Eldridge SM, Chan CL, Campbell MJ, Bond CM, Hopewell S, Thabane L, Lancaster GA. CONSORT 2010 statement: extension to randomised pilot and feasibility trials. bmj. 2016 Oct 24;355. | doi: 10.1136/bmj.i5239

21. Eldridge SM, Costelloe CE, Kahan BC, Lancaster GA, Kerry SM. How big should the pilot study for my cluster randomised trial be?. Statistical methods in medical research. 2016 Jun;25(3):1039-56. doi: 10.1177/0962280215588242

22. Jongsma HE, Turner C, Kirkbride JB, Jones PB. International incidence of psychotic disorders, 2002–17: a systematic review and meta-analysis. The Lancet Public Health. 2019 May 1;4(5):e229-44. doi:10.1016/S2468-2667(19)30056-8

23. Julious SA. Pilot studies in clinical research. Statistical Methods in Medical Research. 2016 Jun;25(3):995-6. doi: 10.1177/0962280216651022

24. National Institute of Health and Clinical Sciences NICE Psychosis and schizophrenia in children and young people: recognition and management. Last updated: 26 October 2016, available at https://www.nice.org.uk/guidance/cg155.

25. Early Psychosis Guidelines Working Group. Australian clinical guidelines for early psychosis. Orygen The National Centre of Excellence in Youth Mental Health: Melbourne, Australia. 2016. doi:10.1007/s00127-015-1140-0

For peer review only - http://bmjopen.bmj.com/site/about/guidelines.xhtml

26. mhGAP training manuals for the mhGAP Intervention Guide for mental, neurological and substance use disorders in non-specialised health settings version 2.0 (for eld testing). Geneva: World Health Organization; 2017 (WHO/MSD/MER/17.6). Licence: CC BY-NC-SA 3.0 IGO. https://www.who.int/mental_health/mhgap/training_manuals/en/

27. Rahman A, Hamdani SU, Awan NR, Bryant RA, Dawson KS, Khan MF, Azeemi MM, Akhtar P, Nazir H, Chiumento A, Sijbrandij M. Effect of a multicomponent behavioral intervention in adults impaired by psychological distress in a conflict-affected area of Pakistan: a randomised clinical trial. Jama. 2016 Dec 27;316(24):2609-17. doi:10.1001/jama.2016.17165

28. Breitborde NJ, Srihari VH, Woods SW. Review of the operational definition for first-episode psychosis. Early intervention in psychiatry. 2009 Nov;3(4):259-65. doi:10.1111/j.1751-7893.2009.00148.x

29. Farooq S, Taylor M. Clozapine: dangerous orphan or neglected friend? The British Journal of Psychiatry. 2011 Apr;198(4):247-9. doi: 10.1192/bjp.bp.110.088690

30. Jakobsen KD, Frederiksen JN, Hansen T, Jansson LB, Parnas J, Werge T. Reliability of clinical ICD-10 schizophrenia diagnoses. Nordic journal of psychiatry. 2005 Jun 1;59(3):209-12. doi: 10.1080/08039480510027698

31. Overall JE, Gorham DR. The brief psychiatric rating scale. Psychological reports. 1962 Jun;10(3):799-812.

32. Young RC, Biggs JT, Ziegler VE, Meyer DA. Young mania rating scale. Journal of Affective Disorders. 2000. doi: 10.1037/t20936-000

33. Williams JB. A structured interview guide for the Hamilton Depression Rating Scale. Archives of general psychiatry. 1988 Aug 1;45(8):742-7. doi:10.1001/archpsyc.1988.01800320058007

34. Haro JM, Kamath SA, Ochoa SO, Novick D, Rele K, Fargas A, Rodriguez MJ, Rele R, Orta J, Kharbeng A, Araya S. The Clinical Global Impression–Schizophrenia scale: a simple instrument to measure the diversity of symptoms present in schizophrenia. Acta Psychiatrica Scandinavica. 2003 May;107:16-23. doi: 10.1034/j.1600-0447.107.s416.5.x.

35. Hall RC. Global assessment of functioning: a modified scale. Psychosomatics. 1995 May 1;36(3):267-75. doi: 10.1016/S0033-3182(95)71666-8

36. Yung A, Phillips L, McGorry P, Ward J, Donovan K, Thompson K. Comprehensive assessment of at-risk mental states (CAARMS). Melbourne, Australia, University of Melbourne, Department of Psychiatry, Personal Assessment and Crisis Evaluation Clinic. 2002 Jan.

37. Group TE. EuroQol-a new facility for the measurement of health-related quality of life. Health policy. 1990 Dec 1;16(3):199-208. doi: 10.1016/0168-8510(90)90421-9

38. Pai S, Kapur RL. The burden on the family of a psychiatric patient: development of an interview schedule. The British Journal of Psychiatry. 1981 Apr;138(4):332-5. doi: 10.1192/bjp.138.4.332

39. Lysaker PH, Davis LW, Warman DM, Strasburger A, Beattie N. Stigma, social function and symptoms in schizophrenia and schizoaffective disorder: Associations across 6 months. Psychiatry research. 2007 Jan 15;149(1-3):89-95. doi: 10.1016/j.psychres.2006.03.007

40. Skinner HA. The drug abuse screening test. Addictive behaviors. 1982 Jan 1;7(4):363-71.

For peer review only

For peer review only - http://bmjopen.bmj.com/site/about/guidelines.xhtml

41. Thompson K, Kulkarni J, Sergejew AA. Reliability and validity of a new Medication Adherence Rating Scale (MARS) for the psychoses. Schizophrenia research. 2000 May 5;42(3):241-7. doi:10.1016/S0920-9964(99)00130-9

42. Tait L, Birchwood MA, Trower P. Predicting engagement with services for psychosis: insight, symptoms and recovery style. The British Journal of Psychiatry. 2003 Feb;182(2):123-8. doi: 10.1192/bjp.182.2.123

43. Addington DE. First Episode Psychosis Services Fidelity Scale (FEPS-FS 1.0) and Manual. University of Calgary Press; 2021.

44. Knapp MR, Knudsen HC, Amaddeo F, Gaite L, van Wijngaarden B. Client Socio-Demographic and Service Receipt Inventory-European Version: Development of an instrument for international research-EPSILON Study 5. British Journal of Psychiatry. 2000;177:S28-33. doi: 10.1192/bjp.177.39.s28

45. Glaser BG, Strauss A. The discovery of grounded theory: theories for qualitative research.1967

46. Corbin JM, Strauss A. Grounded theory research: Procedures, canons, and evaluative criteria. Qualitative sociology. 1990 Mar;13(1):3-21. doi: 10.1007/BF00988593

47. Shepherd TA, Ul-Haq Z, Ul-Haq M, et al Supervised treatment in outpatients for schizophrenia plus (STOPS+): protocol for a cluster randomised trial of a community-based intervention to improve treatment adherence and reduce the treatment gap for schizophrenia in Pakistan BMJ Open 2020;10:e034709. doi: 10.1136/bmjopen-2019-034709

48. Awan NR, Jehangir SF, Irfan M, Naeem F, Farooq S. Explanatory model of illness of the patients with schizophrenia and the role of educational intervention. Schizophrenia Research. 2017 Dec 1;190:68-73. doi: 10.1016/j.schres.2017.03.006

49. Bhikha A, Farooq S, Chaudhry N, Naeem F, Husain N. Explanatory models of psychosis amongst British South Asians. Asian journal of psychiatry. 2015 Aug 1;16:48-54. doi: 10.1016/j.ajp.2015.05.042

50. Lilford P, Rajapakshe OB, Singh SP. A systematic review of care pathways for psychosis in low-and middle-income countries. Asian Journal of Psychiatry. 2020 Dec 1;54:102237. doi: 10.1016/j.ajp.2020.102237

51. Jacob KS. Community care for people with mental disorders in developing countries: problems and possible solutions. The British Journal of Psychiatry. 2001 Apr;178(4):296-8.doi: 10.1192/bjp.178.4.296

52. World Health Organization. Mental health in primary care: illusion or inclusion? World Health Organization; 2018.

53. Lester H, Birchwood M, Freemantle N, Michail M, Tait L. REDIRECT: cluster randomised controlled trial of GP training in first-episode psychosis. British Journal of General Practice. 2009 Jun 1;59(563):e183-90. doi: 10.3399/bjgp09X420851

For peer review only - http://bmjopen.bmj.com/site/about/guidelines.xhtml

Union Councils (UC) in district Peshawar will participate in feasibility cluster RCT

93 Union Councils will be randomised, stratified by Urban/rural setting 1:1

Enhanced Treatment as Usual (ETAU)
20 Traditional Spiritual l Healers (TSH), 5 Primary health care physicians (PHP) selected to provide ETAU as below.

Enhanced Treatment as Usual (ETAU)

- Training in MhGAP for PCP to enhance treatment as usual provided under supervision of two psychiatrists.
- A generic training for TSH about good mental health care provided by PCP.
- Training will be of similar duration to that for the intervention, but content will not include content specifically related to *early intervention* in psychosis but will include generic training for psychosis care included in MhGAP.

- Patients presenting to TSH and PCPs with suspected first episode psychosis (FEP) assessed and referred for further assessments by the research assistant.

- 45 patients with suspected psychosis meeting inclusion and exclusion criteria selected for the RCT.
- Psychiatrists record diagnosis, being blind to 'diagnosis by TSH and PCPs and provide treatment as usual.

THE HOPE Intervention
20 TSH, 5 PHP selected to provide ETAU as below

THE HOPE Intervention

- A cascade model of training and task shifting from psychiatrists to Primary Care Physicians (PCP), and from PCP to TSH for early detection and treatment of FEP in adolescent.
- Psychiatrists, PCPs and TSH trained in early detection and treatment of FEP in adolescent following standard guidelines.
- 20 TSH linked with 5 PHP (4TSH linked to one PCP) with supervision by two psychiatrists for early identification and referral of FEP and establishing community pathways for effective management of FEP.

- Patients presenting to TSH and PCPs with suspected first episode psychosis (FEP) assessed and referred for further assessments by the research assistant.

- 45 patients with suspected psychosis meeting inclusion and exclusion criteria selected for the RCT.
- Psychiatrists record diagnosis, being blind to 'diagnosis by TSH and PCPs and provide treatment following standard guideline in liaison with TSH and PCPs.

Key feasibility outcomes for the trial will include.

- Total number of cases recruited in 12 months period with a minimum sample of 90 in 12-month period.
- Total number of cases referred by TSH and primary care physicians to psychiatrists with suspected FEP during the recruitment period.
- Number of FEP cases suspected by TSH and PCPs during the 12-month period and;
- The likelihood of the confirmed diagnosis of FEP based on interview diagnosis by psychiatrist during the 12-month period and DUP in those meeting criteria for FEP during the 12 month recruitment period

Analysis, process evaluation, dissemination, planning for definitive trial.

Figure 1: The HOPE Flow Diagram

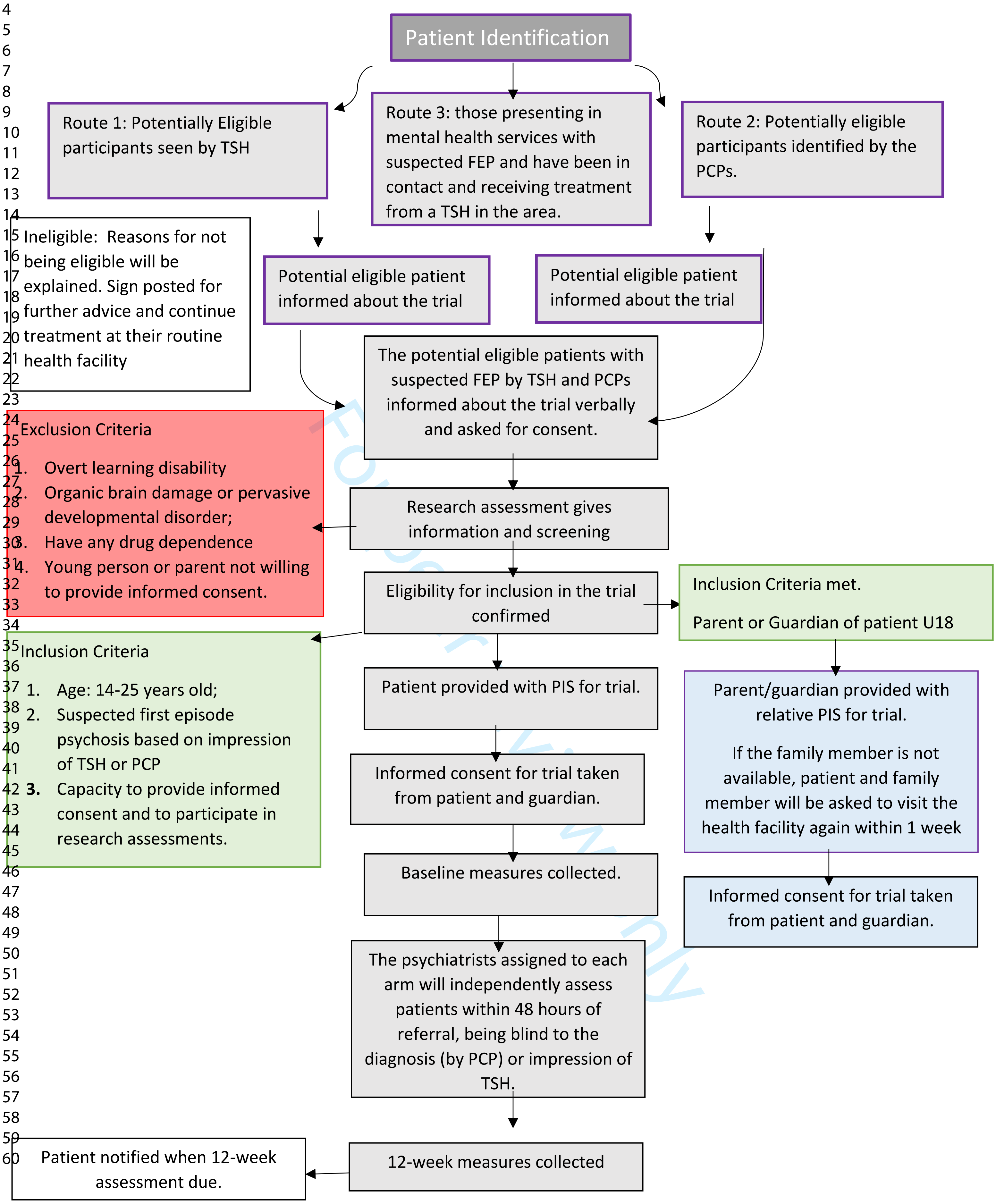

Figure 2. Patient flow and assessments in THE HOPE trial

For peer review only

Appendices

Appendix 1. Participant Consent Form – Young Person 16+

Participant Identification Number:

Participant Consent Form –

Young Person 16+

Study title: Traditional healers working with primary care and mental health for early intervention in psychosis in adolescent: intervention development and feasibility study

This form should be initialled and signed unless the young person is unable to read and write. If they cannot write then the form should be read aloud to the young person and they should be asked to mark the form in the relevant places.

	Please initial/mark box if you agree with the statements
1. I confirm that I have read and understood the information sheet dated 05/Mar/2021 (Version 1.0) for the above study. I have had the opportunity to consider the information, ask questions and have had these answered satisfactorily.	☐
2. I understand that participating in the THEHOPE study involves:<br>- Attending appointments with the research team. This includes; research assistants, primary care physicians, spiritual healers and psychiatrists.<br>- Attending appointments with clinicians where my mental health will be assessed<br>- Receiving treatment for my mental health condition if it is appropriate	☐
3. I understand that my participation is voluntary and that I am free to withdraw at any time without giving any reason and without my legal or medical rights being affected.	☐

For peer review only

4. I understand that all information and data collected during the course of the research will be kept strictly confidential and that any personal data e.g. name and address will only be used for the THEHOPE study.

5. I understand that my data will be pseudonymised through the use of a unique study code.

6. I agree that if the researcher is worried about my safety they may have to tell my doctor or other clinician about things I have told them.

Please continue to next page

7. I understand that I may be invited to take part in research relating to THEHOPE in the future.

8. I voluntarily agree to take part in this study.

Name of Participant	Date	Signature or mark
Name of Researcher taking consent	Date	Signature

1 copy for participant and 1 copy for researcher.

For peer review only

Appendix 2. Participant Consent Form – Parent or Carer

Participant Identification Number:

Participant Consent Form –

Parent or Carer

Study title: Traditional healers working with primary care and mental health for early intervention in psychosis in adolescent: intervention development and feasibility study

This consent form is intended for the parents or caregivers of the young person participating in the study.

This form should be initialled and signed unless the parent or carer is unable to read and write.

If they cannot write then the form should be read aloud and they should be asked to mark the form in the relevant places. This consent procedure must be witnessed by a trusted member of the family or community who is independent to the project. It is necessary that this individual then signs the form at the bottom in the indicated place.

Please initial/mark box if you agree with the statements

9. I confirm that I have read and understood the information sheet dated 05/Mar/2021 (Version 1.0) for the above study. I have had the opportunity to consider the information, ask questions and have had these answered satisfactorily. ☐ ☐

10. I understand that participation of **my child / young person I care for** in the THEHOPE study involves:
    - **My child / young person I care for** attending appointments with the research team. This includes; research assistants, primary care physicians, spiritual healers and psychiatrists.

For peer review only

- Attending appointments with clinicians where **my child / young person I care for** will be assessed for their mental health condition
- **My child / young person I care for** receiving treatment for their mental health condition if it is appropriate

11. I understand that participation of **my child / young person I care for** is voluntary and that we are free to withdraw at any time without giving any reason and without our legal or medical rights being affected. The information I have provided up to that point will be included for research purposes unless I state otherwise. After publication, my information cannot be withdrawn. ☐

Please continue to next page

12. I understand that all information and data collected during the course of the research will be kept strictly confidential and that any personal data e.g. name and address will only be used for the THEHOPE study. ☐

13. I understand that data collected from **my child / young person I care for** will be pseudonymised through the use of a unique study code. ☐

14. I understand and agree that should there be any concern for the safety and welfare of **my child/young person I care for** that the research team may wish to contact their doctor or other clinician. ☐

15. I understand that **my child / young person I care for** may be invited to take part in research relating to THEHOPE in the future. ☐

16. I voluntarily agree for **my child / young person I care for** to take part in this study. ☐

______________________________	______________________	______________
Name of Parent / Carer	Date	Signature or Mark

______________________________	______________________	______________
Name of Researcher	Date	Signature

For peer review only

taking consent

If parent or carer cannot write:

Name and role of trusted observer	Date	Signature
_______________	_______________	_______________

1 copy for participant and 1 copy for researcher.

For peer review only

Reporting checklist for protocol of a clinical trial.

Based on the SPIRIT guidelines.

Instructions to authors

Complete this checklist by entering the page numbers from your manuscript where readers will find each of the items listed below.

Your article may not currently address all the items on the checklist. Please modify your text to include the missing information. If you are certain that an item does not apply, please write "n/a" and provide a short explanation.

Upload your completed checklist as an extra file when you submit to a journal.

In your methods section, say that you used the SPIRIT reporting guidelines, and cite them as:

Chan A-W, Tetzlaff JM, Gøtzsche PC, Altman DG, Mann H, Berlin J, Dickersin K, Hróbjartsson A, Schulz KF, Parulekar WR, Krleža-Jerić K, Laupacis A, Moher D. SPIRIT 2013 Explanation and Elaboration: Guidance for protocols of clinical trials. BMJ. 2013;346:e7586

		Reporting Item	Page Number
**Administrative information**			
Title	#1	Descriptive title identifying the study design, population, interventions, and, if applicable, trial acronym	1
Trial registration	#2a	Trial identifier and registry name. If not yet registered, name of intended registry	1
Trial registration: data set	#2b	All items from the World Health Organization Trial Registration Data Set	NA. Available on trial registry record
Protocol version	#3	Date and version identifier	1
Funding	#4	Sources and types of financial, material, and other support	23

For peer review only

Roles and responsibilities: contributorship	#5a	Names, affiliations, and roles of protocol contributors	1-3,23
Roles and responsibilities: sponsor contact information	#5b	Name and contact information for the trial sponsor	23
Roles and responsibilities: sponsor and funder	#5c	Role of study sponsor and funders, if any, in study design; collection, management, analysis, and interpretation of data; writing of the report; and the decision to submit the report for publication, including whether they will have ultimate authority over any of these activities	23
Roles and responsibilities: committees	#5d	Composition, roles, and responsibilities of the coordinating centre, steering committee, endpoint adjudication committee, data management team, and other individuals or groups overseeing the trial, if applicable (see Item 21a for data monitoring committee)	19
**Introduction**			
Background and rationale	#6a	Description of research question and justification for undertaking the trial, including summary of relevant studies (published and unpublished) examining benefits and harms for each intervention	5-6
Background and rationale: choice of comparators	#6b	Explanation for choice of comparators	5-6
Objectives	#7	Specific objectives or hypotheses	6-7
Trial design	#8	Description of trial design including type of trial (eg, parallel group, crossover, factorial,	7

For peer review only

		single group), allocation ratio, and framework (eg, superiority, equivalence, non-inferiority, exploratory)	
**Methods: Participants, interventions, and outcomes**			
Study setting	#9	Description of study settings (eg, community clinic, academic hospital) and list of countries where data will be collected. Reference to where list of study sites can be obtained	8
Eligibility criteria	#10	Inclusion and exclusion criteria for participants. If applicable, eligibility criteria for study centres and individuals who will perform the interventions (eg, surgeons, psychotherapists)	10-11
Interventions: description	#11a	Interventions for each group with sufficient detail to allow replication, including how and when they will be administered	8-10
Interventions: modifications	#11b	Criteria for discontinuing or modifying allocated interventions for a given trial participant (eg, drug dose change in response to harms, participant request, or improving / worsening disease)	16-17
Interventions: adherence	#11c	Strategies to improve adherence to intervention protocols, and any procedures for monitoring adherence (eg, drug tablet return; laboratory tests)	14-15
Interventions: concomitant care	#11d	Relevant concomitant care and interventions that are permitted or prohibited during the trial	NA. Does not apply
Outcomes	#12	Primary, secondary, and other outcomes, including the specific measurement variable	12-15

For peer review only

		(eg, systolic blood pressure), analysis metric (eg, change from baseline, final value, time to event), method of aggregation (eg, median, proportion), and time point for each outcome. Explanation of the clinical relevance of chosen efficacy and harm outcomes is strongly recommended	
Participant timeline	#13	Time schedule of enrolment, interventions (including any run-ins and washouts), assessments, and visits for participants. A schematic diagram is highly recommended (see Figure)	13-14, 17
Sample size	#14	Estimated number of participants needed to achieve study objectives and how it was determined, including clinical and statistical assumptions supporting any sample size calculations	8
Recruitment	#15	Strategies for achieving adequate participant enrolment to reach target sample size	11
**Methods: Assignment of interventions (for controlled trials)**			
Allocation: sequence generation	#16a	Method of generating the allocation sequence (eg, computer-generated random numbers), and list of any factors for stratification. To reduce predictability of a random sequence, details of any planned restriction (eg, blocking) should be provided in a separate document that is unavailable to those who enrol participants or assign interventions	8

For peer review only

Allocation concealment mechanism	#16b	Mechanism of implementing the allocation sequence (eg, central telephone; sequentially numbered, opaque, sealed envelopes), describing any steps to conceal the sequence until interventions are assigned	NA (Cluster RCT)
Allocation: implementation	#16c	Who will generate the allocation sequence, who will enrol participants, and who will assign participants to interventions	11,12 Allocation sequence generation is not relevant (cluster RCT).
Blinding (masking)	#17a	Who will be blinded after assignment to interventions (eg, trial participants, care providers, outcome assessors, data analysts), and how	16
Blinding (masking): emergency unblinding	#17b	If blinded, circumstances under which unblinding is permissible, and procedure for revealing a participant's allocated intervention during the trial	16
**Methods: Data collection, management, and analysis**			
Data collection plan	#18a	Plans for assessment and collection of outcome, baseline, and other trial data, including any related processes to promote data quality (eg, duplicate measurements, training of assessors) and a description of study instruments (eg, questionnaires, laboratory tests) along with their reliability and validity, if known. Reference to where data collection forms can be found, if not in the protocol	11-16
Data collection plan: retention	#18b	Plans to promote participant retention and complete follow-up, including list of any outcome data to be collected for participants	NA. No specific plans to collect data from patients who discontinue and deviations

For peer review only

		who discontinue or deviate from intervention protocols	from the protocol will be reported to DSMB and TSC.
Data management	#19	Plans for data entry, coding, security, and storage, including any related processes to promote data quality (eg, double data entry; range checks for data values). Reference to where details of data management procedures can be found, if not in the protocol	16-17
Statistics: outcomes	#20a	Statistical methods for analysing primary and secondary outcomes. Reference to where other details of the statistical analysis plan can be found, if not in the protocol	18-19
Statistics: additional analyses	#20b	Methods for any additional analyses (eg, subgroup and adjusted analyses)	18-19
Statistics: analysis population and missing data	#20c	Definition of analysis population relating to protocol non-adherence (eg, as randomised analysis), and any statistical methods to handle missing data (eg, multiple imputation)	NA. feasibility trial
**Methods: Monitoring**			
Data monitoring: formal committee	#21a	Composition of data monitoring committee (DMC); summary of its role and reporting structure; statement of whether it is independent from the sponsor and competing interests; and reference to where further details about its charter can be found, if not in the protocol. Alternatively, an explanation of why a DMC is not needed	19
Data monitoring: interim analysis	#21b	Description of any interim analyses and stopping guidelines, including who will have access to these interim results and make the final decision to terminate the trial	NA. No interim analyses planned, in case of harm reported refer to pg 17.

For peer review only

Harms	#22	Plans for collecting, assessing, reporting, and managing solicited and spontaneously reported adverse events and other unintended effects of trial interventions or trial conduct	19,20
Auditing	#23	Frequency and procedures for auditing trial conduct, if any, and whether the process will be independent from investigators and the sponsor	19
**Ethics and dissemination**			
Research ethics approval	#24	Plans for seeking research ethics committee / institutional review board (REC / IRB) approval	19
Protocol amendments	#25	Plans for communicating important protocol modifications (eg, changes to eligibility criteria, outcomes, analyses) to relevant parties (eg, investigators, REC / IRBs, trial participants, trial registries, journals, regulators)	20
Consent or assent	#26a	Who will obtain informed consent or assent from potential trial participants or authorised surrogates, and how (see Item 32)	11
Consent or assent: ancillary studies	#26b	Additional consent provisions for collection and use of participant data and biological specimens in ancillary studies, if applicable	NA. These will be given in detail in the informed consent form.
Confidentiality	#27	How personal information about potential and enrolled participants will be collected, shared, and maintained in order to protect confidentiality before, during, and after the trial	20

For peer review only

Declaration of interests	#28	Financial and other competing interests for principal investigators for the overall trial and each study site	23
Data access	#29	Statement of who will have access to the final trial dataset, and disclosure of contractual agreements that limit such access for investigators	16-17
Ancillary and post trial care	#30	Provisions, if any, for ancillary and post-trial care, and for compensation to those who suffer harm from trial participation	NA. This is organizational level intervention providing psychosocial and is unlikely to result in serious harm to participants. KMU will provide post trial care in case of any serious harm.
Dissemination policy: trial results	#31a	Plans for investigators and sponsor to communicate trial results to participants, healthcare professionals, the public, and other relevant groups (eg, via publication, reporting in results databases, or other data sharing arrangements), including any publication restrictions	20
Dissemination policy: authorship	#31b	Authorship eligibility guidelines and any intended use of professional writers	NA (No professional writers sought)
Dissemination policy: reproducible research	#31c	Plans, if any, for granting public access to the full protocol, participant-level dataset, and statistical code	20
**Appendices**			
Informed consent materials	#32	Model consent form and other related documentation given to participants and authorised surrogates	Refer to appendices (supplementary file)
Biological specimens	#33	Plans for collection, laboratory evaluation, and storage of biological specimens for	NA

For peer review only

		genetic or molecular analysis in the current trial and for future use in ancillary studies, if applicable	

None The SPIRIT Explanation and Elaboration paper is distributed under the terms of the Creative Commons Attribution License CC-BY-NC. This checklist can be completed online using https://www.goodreports.org/, a tool made by the EQUATOR Network in collaboration with Penelope.ai

For peer review only

Traditional healers working with primary care and mental health for early intervention in psychosis in young persons: Protocol for the feasibility cluster randomised controlled trial

Journal:	*BMJ Open*
Manuscript ID	bmjopen-2023-072471.R1
Article Type:	Protocol
Date Submitted by the Author:	15-May-2023
Complete List of Authors:	Farooq, Saeed; Keele University, School of Primary, Community and Social Care; Midlands Partnership NHS Foundation Trust<br>Sheikh, Saima; Keele University, School of Primary, Community and Social Care<br>Dikomitis, Lisa; University of Kent, Kent and Medway Medical School<br>Ul-Haq, Mian; Lady Reading Hospital, Medical Teaching Institution<br>Khan, Abdul Jalil; Khyber Medical University, Institute of Public Health & Social Sciences<br>Sanauddin, Noor; University of Peshawar, Department of Sociology<br>Malik, Wajid; Combined Military Hospital<br>Ali, Johar; University of Peshawar, Department of Sociology<br>Khan, Muhammad Firaz; Lady Reading Hospital, Medical Teaching Institution<br>Chaudhry, Imran; Pakistan Institute of Living and Learning<br>Husain, Nusrat; The University of Manchester, Division of Psychology and mental Health<br>Gul, Muhammad; Midlands Partnership NHS Foundation Trust, Research and Innovation<br>Irfan, Muhammad; Peshawar Medical College, Department of Mental Health, Psychiatry & Behavioral Sciences; Riphah International University<br>Andrews, Gabrielle; Midlands Partnership NHS Foundation Trust, Research and Innovation<br>Kaistha, Prachi; Keele University, School of Medicine<br>Shah, Syed Muhammad Uzair; University of Peshawar, Department of Sociology<br>Azeemi, Ishfaq; University of Peshawar, Department of Sociology<br>Hamid, Shumaila; Khyber Medical University, Institute of Public Health & Social Sciences<br>Minhaz, Aaliya; Shaheed Benazir Bhutto Women University<br>Mallen, Christian; Keele University, School of Primary, Community and Social Care<br>Lewis, Martyn; Keele University, School of Primary, Community and Social Care
**Primary Subject Heading**:	Mental health
Secondary Subject Heading:	Health services research, Medical education and training, Mental health,

For peer review only - http://bmjopen.bmj.com/site/about/guidelines.xhtml

	Public health
Keywords:	MENTAL HEALTH, PUBLIC HEALTH, EPIDEMIOLOGIC STUDIES, Child & adolescent psychiatry < PSYCHIATRY, MEDICAL EDUCATION & TRAINING, Schizophrenia & psychotic disorders < PSYCHIATRY

SCHOLARONE™
Manuscripts

Traditional healers working with primary care and mental health for early intervention in psychosis in young persons: Protocol for the feasibility cluster randomised controlled trial

Saeed Farooq [1, 2,] Saima Sheikh[1], Lisa Dikomitis[3], Mian Mukhtar- Ul- Haq Azeemi [4], Abdul Jalil Khan [5,] Noor Sanauddin[6], Malik Wajid Ali [7], Johar Ali [6] Muhammad Firaz Khan [4], Imran Chaudhry [8, 9], Nusrat Husain [9,10], Muhammad Gul [2], Muhammad Irfan [11], Gabrielle Andrews [1,2], Prachi Kaistha [1], Shah, Syed Muhammad Uzair[6], Ishfaq Azeemi [6], Shumaila Hamid [5], Aaliya Minhaz [12], Christian Mallen [1], Martyn Lewis [1]

1. School of Primary, Community and Social Care, Keele University, Keele, Staffordshire, UK
2. Research and Innovation, Midlands Partnership Foundation Trust, Staffordshire, UK
3. Kent and Medway Medical School, University of Kent, Kent, UK
4. Medical Teaching Institution, Lady Reading Hospital, Peshawar, Khyber Pakhtunkhwa, Pakistan
5. Institute of Public Health & Social Sciences, Khyber Medical University, Peshawar, Khyber Pakhtunkhwa, Pakistan
6. Department of Sociology, University of Peshawar, Peshawar, Khyber Pakhtunkhwa, Pakistan
7. Consultant Psychiatrist Combined Military Hospital, Rawalpindi
8. Pakistan Institute of Living and Learning, Karachi, Pakistan
9. Division of Psychology and Mental Health, University of Manchester, Manchester, UK
10. Research and Innovation, Merseycare NHS Foundation Trust, Liverpool, UK
11. Peshawar Medical College, Peshawar, Khyber Pakhtunkhwa, Pakistan
12. Shaheed Benazir Bhutto Women University, Peshawar, Khyber Pakhtunkhwa, Pak

Correspondence to Dr Saeed Farooq; s.farooq@keele.ac.uk

Professor of Psychiatry and Public Mental Health

School of Medicine, Keele University, Staffordshire, ST5 5BG, UK.

Honorary Consultant Psychiatrist,

Midlands Partnership NHS Foundation Trust.

For peer review only - http://bmjopen.bmj.com/site/about/guidelines.xhtml

For peer review only

For peer review only - http://bmjopen.bmj.com/site/about/guidelines.xhtml

	Name	Contact	Postal Address
1	Professor Saeed Farooq	s.farooq@keele.ac.uk	School of Primary, Community and Social Care, Keele University, Keele, Staffordshire, UK, ST5 5BG
2	Saima Sheikh	s.sheikh@keele.ac.uk	School of Primary, Community and Social Care, Keele University, Keele, Staffordshire, UK, ST5 5BG
3	Professor Lisa Dikomitis	lisa.dikomitis@kmms.ac.uk	Kent and Medway Medical School, University of Kent, Kent, UK CT2 7FS
4	Dr Mian Mukhtar -Ul- Haq	doctormian@yahoo.co.uk	Medical Teaching Institution, Lady Reading Hospital, Peshawar, Khyber Pakhtunkhwa, 25000 Pakistan
5	Dr Abdul Jalil Khan	jalilkhan@kmu.edu.pk	Institute of Public Health & Social Sciences, Khyber Medical University, Peshawar, Khyber Pakhtunkhwa,25100, Pakistan
6	Dr Noor Sanauddin	noor.sanauddin@uop.edu.pk	Department of Sociology, University of Peshawar, Peshawar, Khyber Pakhtunkhwa, 25120, Pakistan
7	Dr Malik Wajid Ali	malikwajid10@gmail.com	Consultant Psychiatrist Combined Military Hospital, Rawalpindi
8	Professor Johar Ali	johar_ali55@yahoo.com	Department of Sociology, University of Peshawar, Peshawar, Khyber Pakhtunkhwa, 25120, Pakistan
9	Muhammad Firaz Khan	drfiraz786@yahoo.co.uk	Medical Teaching Institution, Lady Reading Hospital, Peshawar, Khyber Pakhtunkhwa, 25000, Pakistan
10	Professor Imran Chaudhry	Imran.chaudhry@pill.ac.uk	Pakistan Institute of Living and Learning, Karachi, Sindh 75600, Pakistan
11	Professor Nusrat Husain	Nusrat.husain@manchester.ac.uk	Division of Psychology and Mental Health, University of Manchester, Manchester, UK M13 9PL
12	Dr Muhammad Gul	Muhammad.Gul@mpft.nhs.uk	Research and Innovation, Midlands Partnership Foundation Trust, Staffordshire, UK, ST16 3SR
13	Professor Muhammad	mirfan78@yahoo.com	Peshawar Medical College, Peshawar, Khyber Pakhtunkhwa, Pakistan

For peer review only - http://bmjopen.bmj.com/site/about/guidelines.xhtml

	Irfan		
14	Gabrielle Andrews	gabrielle.andrews@mpft.nhs.uk	Research and Innovation, Midlands Partnership Foundation Trust, Staffordshire, UK
15	Prachi Kaistha	p.k.kaistha@keele.ac.uk	School of Primary, Community and Social Care, Keele University, Keele, Staffordshire, UK, ST5 5BG
16	Shah, Syed Muhammad Uzair	uzairshah2011@yahoo.com	Department of Sociology, University of Peshawar, Peshawar, Khyber Pakhtunkhwa, 25120, Pakistan
17	Ishfaq Azeemi	ishfaqazeemi3@gmail.com	Department of Sociology, University of Peshawar, Peshawar, Khyber Pakhtunkhwa, 25120, Pakistan
18	Shumaila Hamid	drshumailahk@gmail.com	Institute of Public Health & Social Sciences, Khyber Medical University, Peshawar, Khyber Pakhtunkhwa,25100, Pakistan
19	Aliya Minhas	aaliyachem@sbbwu.edu.pk	Shaheed Benazir Bhutto Women University, Peshawar, Khyber Pakhtunkhwa, Pakistan
20	Professor Christian Mallena	c.d.mallen@keele.ac.uk	School of Primary, Community and Social Care, Keele University, Keele, Staffordshire, UK, ST5 5BG
21	Dr Martyn Lewis	a.m.lewis@keele.ac.uk	School of Primary, Community and Social Care, Keele University, Keele, Staffordshire, UK, ST5 5BG

For peer review only

For peer review only - http://bmjopen.bmj.com/site/about/guidelines.xhtml

Abstract

Objectives: **I**n Low- and Middle-Income Countries (LMICS), more than half of patients with First Episode Psychosis initially seek treatment from traditional and religious healers as their first care. This contributes to an excessively long duration of untreated Psychosis (DUP). There is a need for culturally appropriate interventions to involve traditional and spiritual healers (TSH) to work collaboratively with primary care practitioners (PCPs) and psychiatrists through task shifting for early detection, referral, and treatment of First Episode of Psychosis (FEP).

Methods: To prevent the consequences of long DUP in adolescents in LMICS, we aim to develop and pilot test a culturally appropriate and context-bespoke intervention. **T**raditional **HE**alers working with primary care and mental **H**ealth for early interventi**O**n in **P**sychosis in young p**Er**sons (THE HOPE) will be developed using ethnographic and qualitative methods with traditional healers and caregivers. We will conduct a randomised controlled cluster feasibility trial with a nested qualitative study to assess study recruitment and acceptability of the intervention. Ninety-three union councils in district Peshawar, Pakistan, will be randomised and allocated using a 1:1 ratio to either intervention arm (THE HOPE) or Enhanced Treatment as Usual (ETAU) and stratified by urban/rural setting. Data on feasibility outcomes will be collected at baseline and follow-up. Patients, carers, clinicians, and policymakers will be interviewed to ascertain their views about the intervention. The decision to proceed to the phase III trial will be based on prespecified stop-go criteria.

Ethics and dissemination: Ethical approval has been obtained from Keele University Ethical Review Panel (ref: MH210177), Khyber Medical University Ethical Review Board (ref: DIR/KMU-EB/IG/001005) and National Bioethics Committee Pakistan (Ref: No.4-87/NBC-840/22/621). The results of THE HOPE feasibility trial will be reported in peer-reviewed journals and academic conferences and disseminated to local stakeholders and policymakers.

Trial registration*:* ISRCTN75347421 https://doi.org/10.1186/ISRCTN75347421

Word count: 6437

For peer review only - http://bmjopen.bmj.com/site/about/guidelines.xhtml

Strengths and Limitations:

- The proposed study seeks to develop and pilot test a novel and culturally appropriate intervention for early detection, referral, and treatment of First Episode of Psychosis with the aid of traditional spiritual healers to prevent consequences of untreated psychosis in resource-limited settings.
- The present study aims to integrate mental health and primary care services to provide early intervention in untreated psychosis in collaboration with traditional healers and evaluate it in a feasibility randomised controlled trial.
- We will use a mixed methods design, comprising both quantitative and qualitative methods, to develop, test and evaluate the feasibility of the intervention and understand its strengths and limitations.
- A limitation of this feasibility cluster RCT is that the planned study has a small study and is not powered to test the effectiveness of the intervention. This being a feasibility trial will evaluate the acceptability of the intervention and will provide precision estimates for an adequately powered definitive randomised controlled trial in future.

review only

Introduction:

Background and Rationale:

First Episode Psychosis (FEP) has peak onset between 15-35 years of age.[1,2,3] FEP can disrupt education, reduce long-term employment, causes huge economic burden and is associated with significantly higher mortality as compared to the general population.[4] Duration of Untreated Psychosis (DUP), the period between the onset of psychosis and initiating treatment is 125 weeks in low-and middle-income countries (LMICs) compared to 62.5 weeks in high-income countries (HICs).[5] Even when initiated, the treatment is mostly limited to acute episodes resulting in a huge treatment gap with around 70% of people not receiving regular care[6,7] with incidence of self-harm during long periods of untreated psychosis.[5]

A major factor for the long DUP in LMICs is the preference of the majority of the patients to see traditional and spiritual healers (TSHs) as their first care providers for mental disorders.[8] This is consistent with a systematic review showing predominantly supernatural explanatory models of illness for psychosis in LMICs.[9] Research with TSHs in Pakistan[10,11,12] has identified that the following terms are used for identifying people suffering from psychosis: influence of jinn, saya (influence or shadow), 'strong spell or under the control of spirits' lewanay (insane) and 'superstitious'. This is consistent with previous literature.[11,12] These healers use different methods of treatment,

including de-possession from jinn or saya, instruction and advice regarding prayers, Tawiz (symbolic lines and verses written on a piece of paper), Dam (Holy verses recited and blown on the body), Dua (prayers), and special religious ceremonies which includes reciting holy verses loudly and family accompanying the spiritual healers in a trance like state and exorcism to remove the spirits from home and offering holy water.[11,12] Other methods for treatment include homoeopathy, danyalism (shamanism), the holy Tabbarak (blessed food), sugar or salt, drinking Dam Wala Pani (sacred verses recited on water) available at Dargahs (shrines).[12, 13]

Another key factor in the long DUP is the lack of primary care involvement in the treatment of psychosis.[14] The integration of mental health and primary care services is widely advocated, especially in LMIC, and primary care practitioners have an important role in providing effective early intervention in psychosis.[15] However, the evidence suggests that primary care physicians have practically no role in the treatment of psychosis in LMIC.[14]

Evidence available in other disease areas, such as HIV/AIDS, suggests that with appropriate training and support, services have been scaled up in collaboration with traditional healers.[16] However, there are hardly any interventions for improving collaboration with TSH in mental health. A systematic review on the effectiveness of TSHs in treating mental disorders highlighted limited interventions mostly described in generally poor-quality studies with no intervention found involving TSHs in the care of FEP.[17] There is an urgent need to work collaboratively with TSH and develop and rigorously evaluate EIP interventions in LMICs, as TSHs have far greater influence in treating mental disorders, and a pilot study showed that TSHs are open to identifying and referring individuals with possible psychosis.[18]

Aims and Objectives:

To prevent the consequences of long DUP in young persons in LMICS, we aim to develop and pilot test a culturally appropriate and context-bespoke intervention (THE HOPE) for early identification, referral, and management of FEP in the young population in Peshawar, Khyber Pakhtunkhwa (KP) in Pakistan and evaluate its feasibility for implementation. THE HOPE intervention will involve TSHs working collaboratively with primary care practitioners (PCPs) and psychiatrists through task shifting for early detection, referral, and treatment of FEP.

Objectives:

1. To co-develop with key stakeholders a culturally appropriate intervention for early identification, referral, and management of FEP.
2. To investigate the acceptability of task sharing, training procedures and to establish pathways for referral and management of FEP in consultation with all stakeholders.
3. To establish the feasibility and acceptability of involving mental health service users in training and feasibility study methods.

For peer review only - http://bmjopen.bmj.com/site/about/guidelines.xhtml

4. To evaluate the acceptability and feasibility of THE HOPE in terms of changes in knowledge and referral to mental health services by TSH and users' satisfaction.
5. To estimate the parameters (e.g., recruitment and retention rates), means and standard deviations of the key outcome measures to benchmark potential outcome measures and enable sample size calculations for future pragmatic RCT.
6. To assess the best methods for implementing and evaluating the intervention in the health care system.

Methods and analysis:

The research plan for THE HOPE study includes a development phase to robustly co-design and co-produce THE HOPE intervention following the MRC framework for developing and evaluating complex interventions.[19] This paper describes in detail the methodology of the pragmatic cluster pilot RCT and the intervention development process is given here briefly.

The intervention will be co-developed working with TSH, PCP and service users through a series of (i) culturally appropriate interactive community engagement activities, (ii) ethnographic studies and (iii) qualitative studies. Trained ethnographers will participate in up to 15 different communal settings involving interactions with TSH, healthcare workers, patients and carers. Ethnographic data will provide insights for intervention development and will inform about disease awareness and indigenous knowledge, stigma, lived experience of the disease in adolescents, effects on autonomy, the concept of psychosis, impact on social relations, local lay referral system, any harmful practices used by TSH, and triggers, barriers and facilitators to seeking formal healthcare.

The findings of the ethnography will feed into the topic guides for Semi-Structured Interviews (SSI) and Focus Group Discussions (FGD) that will be undertaken with parents, especially mothers, who have a critical role in initiating psychiatric treatment for their child with FEP. We will conduct SSI (*n*=~25) with both patients and carers) and six FGDs, with 8-12 participants, including TSH, primary care physicians, psychiatrists, and other health workers. These SSIs and FGDs will be conducted in the local Pashto language to explore perceptions, understandings and experiences with FEP of patients and carers, involvement of young persons in decision making, ways to improve early detection of psychosis, views regarding the nature of symptoms in young people, experiences of receiving treatment from TSH, barriers and facilitators to early treatment, training needs for TSH and professionals and best methods to deliver the intervention.

This development phase will inform the development of THE HOPE intervention (see below), which will be evaluated in a cluster feasibility RCT.

Study Design:

We will use a pragmatic cluster pilot RCT to test the feasibility and acceptability of the intervention. The study will be reported using the CONSORT extension for randomised pilot and feasibility trials.[20]

For peer review only - http://bmjopen.bmj.com/site/about/guidelines.xhtml

Study Setting:

The study will be carried out in Peshawar district, the capital city of KP province in Pakistan. District Peshawar has a population of about 4 million. The district has 93 union councils (UC) as basic administrative divisions. Each UC has, on average, a population of about 20000 and is served by a primary health care centre called Basic Health Unit (BHU), providing essential primary health care services. The mental health services are provided mainly in 3 teaching hospitals in Peshawar with about 20 qualified psychiatrists.

Randomisation:

Randomisation will occur at the Union Council level and be allocated using a 1:1 ratio to either the intervention arm (THE HOPE) or Enhanced Treatment as Usual (ETAU) and stratified by urban/rural setting.

Sample Size:

This being a feasibility study, we do not provide a power calculation as there is no proposal for formal hypothesis testing, and the sample size focuses on the required precision of feasibility parameters in line with recommendations for pilot and feasibility studies.[21] First Episode Psychosis has a relatively low incidence of about 26·6 per 100 000 person-years.[22] In order to obtain an adequate sample size to measure feasibility outcomes, we will include all union councils in district Peshawar (n=93) in the sample, allocated to ETAU and THE HOPE arm in a 1:1 ratio. We will aim to recruit a minimum of 90 patients (45 in each arm) over 12 months period. We believe this will help us to achieve reasonable precision of feasibility estimates, e.g., for evaluation of intervention fidelity and participant retention in the study (as a proportion). This will enable the 90% one-sided lower confidence bound to be derived to a margin of error of 0.1 for single-arm evaluation and 0.07 for overall sample evaluation. This fits the criteria for sample size justification and calculation recommendations for pilot and feasibility studies.[23]

The intervention and control arms: (see Figure 1)

The Intervention Arm

THE HOPE intervention arm will receive THE HOPE intervention in addition to Enhanced Treatment As Usual (ETAU). THE HOPE is an organisational/service-level intervention with the following essential components:

1. **A cascade model** of training and task shifting from psychiatrists to Primary Care Physicians (PCP) and from PCP to TSH for early detection and treatment of FEP in adolescents.

For peer review only - http://bmjopen.bmj.com/site/about/guidelines.xhtml

2. **Linking TSH and PCP** with mental health professionals for early identification and referral of FEP and establishing community pathways for effective management of FEP

A group of 4 TSH will be linked with one PCP working in the nearest Basic Health Unit (BHU). PCPs trained in early identification and referral of FEP in adolescents in WP1 will work collaboratively with the TSH and psychiatrist supervising the treatment.

. The TSH and PCPs will work collaboratively in establishing care pathways for the optimum management of FEP in young persons.

The cascade training for early identification and management will consist of the following:

Training for Psychiatrists:

First, a team of psychiatrists with extensive experience in early intervention in psychosis and child and adolescent psychiatry in the UK and Pakistan will train local psychiatrists in the early detection and treatment of FEP. We will train two psychiatrists who will act as Master Trainers (MTs). These master trainers, in turn, will train PCPs. We will use face-to-face teaching, case studies and direct clinical supervision. The training will be based on recent guidelines[24,25] on the early detection and management of psychosis in young people [24] adopted for the local settings. MhGAP guidelines on Training of Trainers[26] will be used for training master trainers, and quality assured by Khyber Medical University (KMU) University.

Training for Primary Care Physicians (PCPs):

Secondly, these MTs will train PCPs using interactive methods. PCPs in both arms will be trained in the mhGAP treatment guidelines, while those in the intervention arm will receive additional training in the early detection and management of FEP by MTs, as described above.

Training for Traditional and Spiritual Healers (TSH):

Thirdly, these PCPs will train TSH for identification, referral and working collaboratively with PCPS and health services for effective management of FEP. The information gained from ethnographic and qualitative studies will inform the contents, format and methodology for training TSH. Based on our experience of working with traditional healers in the STOPS+ programme[10], the training will mainly focus on the following topics: overcoming barriers in working with psychiatric services, symptoms and signs of FEP and differentiating these from normal behaviours expected in adolescence, attitudes and behaviours towards people with psychosis, avoiding harmful practices, respecting individual autonomy, preventing gender-based discrimination and information about referral pathways. The training will be delivered in a workshop.

Two main educational tools used in workshops will be (i) case vignettes of typical patients for identification of suspected cases of FEP, which we have successfully used in our previous research with lay health workers, and (ii) a video in the local language that will deliver the key messages on topics mentioned above.

For peer review only - http://bmjopen.bmj.com/site/about/guidelines.xhtml

We have used this model of task shifting and cascade training in our previous research[27] and will use a similar approach in implementing the intervention.

Control arm: Enhanced Treatment as Usual (ETAU):

This arm will include treatment as usual, enhanced by mhGAP training for PCPs and generic training for TSH about good mental health. Training will be of a similar duration to that for the intervention but will not include content specifically related to early intervention in psychosis but will include generic training for psychosis care included in mhGAP. ETAU provided in the control clusters will include the treatment received by patients' routine healthcare setting, which includes treatment provided by a psychiatrist in the outpatient clinics of the psychiatry department of the local hospital and brief counselling about the treatment and outcome of the disorder.

A group of four TSH will be linked with one PCP working in the nearest Basic Health Unit (BHU). The PCPs in the control group will be trained in the general management of psychosis provided in the MhGAP manual.

Identification of the PCPs and TSHs:

We will select 20 TSH who will be linked with five primary care physicians (PCPs) working in the local primary health care centres in each arm of the study. A group of five TSH will be linked with one PCP working in the nearest PHC. The PCPs will be randomly selected in both intervention and control arms and will be linked to TSHs. The selection will be made by The Department of Family Medicine at Khyber Medical University.

Our previous work in this area has identified three types of TSH: (i) Pirs (spiritual leaders in Sufi tradition) with well-established shrines, (ii) imams, spiritual leaders based in mosques and (iii) traditional healers who practice traditional methods such as (taweez) alms or herbs. After consultations with community elders, 20 TSH in each arm will be selected based on (i) an established reputation for treating mental health problems and not using harmful practices and (ii) willingness to work with the health care system.

Patient and Public Involvement

HOPE comprises a novel culturally appropriate early intervention for FEP that will involve patient and public community engagement at every stage of the research (intervention development, feasibility RCT and process evaluation). Key stakeholders, including service users, care providers, cultural groups, community experts and policymakers, will be consulted at each stage of the research process. Keele Patient and Public Involvement and Engagement (PPIE) group and service users' group in Peshawar, Pakistan, provided key inputs in developing the study design.

For peer review only - http://bmjopen.bmj.com/site/about/guidelines.xhtml

Participant Eligibility Criteria:

We will recruit patients (aged 14—25 years) residing in one of the union councils in district Peshawar with (a) First ever psychotic episode who have not received antipsychotic medication previously, or if they already have used antipsychotic medications, it was for no longer than six weeks.[28] The FEP diagnosis will be made at an interview by psychiatrists based on the International Classification of Diseases, 11th revision (ICD-11) criteria for schizophrenia, persistent delusional disorder, acute and transient psychotic disorders, schizoaffective disorder, mood disorders (mania, severe depressive episode, bipolar affective disorder) with psychotic symptoms.

The exclusion criteria will be (i) evidence of overt learning disability, (ii) organic brain damage or pervasive developmental disorder; (iii) severe substance abuse (except nicotine dependence) and a young person or parent not willing to provide informed consent.

Recruitment:

Participants suspected of having FEP will be identified from the following possible sources (1) potentially eligible participants seen by TSH, (2) potentially eligible participants identified by the PCPs, (3) those presenting in mental health services with suspected FEP and have been in contact and receiving treatment from a TSH in the area.

The cluster allocation will determine the treatment path for each participant. It is possible, for example, that a potentially eligible patient residing in the control cluster presents to the TSH in the intervention arm and vice versa. In such a case, the patient will be given the choice of visiting the nearest TSH based in the control arm (and vice versa). Otherwise, he/she will not be included in the study.

To avoid any selection bias in both groups, the recruitment into the study (both arms) would be done using the same methods by an independent researcher who would enrol in a non-selection-bias way that gives parity/equality in uptake. The baseline information about all suspected cases of psychosis by TSH will be collected, including symptomatology and demographic features. Those with a confirmed diagnosis of psychosis (by the psychiatric team following the procedures given below) will be treated in one of the treatment arms.

Potential participants will be verbally informed about the trial by the TSH, PCPs or RA working with the research team. Patients who agree to participate will be given a full written information sheet about the study by a research assistant (RA) (written information sheets read by RA for those who cannot read) about the study. In case of a young person below the age of 16, written informed consent will be obtained from the patient and the parent, who will almost always be accompanying the young person (Appendix 1 and 2). For illiterate participants, witnessed oral consent and a thumbprint in lieu of a signature will be sufficient. The witness will not be a member of the research team. Both alternatives to full written consent are routinely practised in Pakistan, are culturally acceptable and have been used in our previous studies in this setting [27, 29]. See Figure 2.

For peer review only - http://bmjopen.bmj.com/site/about/guidelines.xhtml

Those willing to participate will be requested to complete the baseline socio-demographic data and a suicide risk screen based on the MhGAP intervention guide by a research assistant (RA). In case of any concerns about the safety of the individual due to suicide risk, the RA will seek help from psychiatric services following the study SOP developed for this purpose.

Study procedures and data collection (see Figure 2):

The RA will refer all suspected cases from TSH and PCP to the psychiatrists assigned to each arm, who will independently assess patients within 48 hours of referral, being blind to the diagnosis (by PCP) or impression of TSH. The 'diagnosis' of FEP by TSH will be recorded using an instrument developed in consultation with TSH. This instrument will indicate the likelihood of a person suffering from FEP, as judged by the TSH. Separately, TSH will also be asked to identify the lay terminology they use in their routine work to capture their conceptualisation of FEP. For patients referred from primary care, PCPs will be asked to provide the likely diagnosis based on ICD-10.[30]

The Psychiatrists will be trained to achieve adequate interrater reliability in making the FEP diagnosis. The psychiatrists will diagnose based on the ICD-10 criteria of first-episode psychosis described above.

Those diagnosed with FEP will be started on treatment in collaboration with participating PCPs or TSH (if the patients and family want to continue treatment by TSH) in the intervention arm, while those in the control arm will be provided with the treatment as usual. The treatment will be based on recent guidelines for the treatment of FEP adopted for the purpose of the study.

Quantitative Data Collection and Follow-up:

Once the participant has provided informed consent, trained RAs will collect the demographic information and complete baseline assessment and suicide risk screen based on the MhGAP intervention guide. Follow-up data will be collected 12 weeks post-baseline by the RAs blinded to the allocation status.

For peer review only - http://bmjopen.bmj.com/site/about/guidelines.xhtml

Outcome Measures

Primary outcome measures: Key feasibility outcomes for the trial will include the total number of cases recruited in the 12-month period and cases referred by TSH and primary care physicians to psychiatrists with suspected FEP during the recruitment period. It will also include the number of FEP cases suspected by TSH and PCPs and the likelihood of the confirmed diagnosis of FEP based on interview diagnosis by psychiatrist and DUP in those meeting criteria for FEP.

Secondary outcome measures: In addition to feasibility outcomes, the study will aim to measure a range of clinical outcomes and service utilisation measures for assessing the feasibility and acceptability of the intervention. In addition,_process evaluation (see below) will be done using qualitative methods. Table 1 provides details of these outcomes and the baseline and follow-up points for these assessments.

For peer review only

1. **Psychopathology:** Brief Psychiatric Rating Scale (BPRS)[31], Young Mania Rating Scale (YMRS)[32,] Hamilton Depression Rating Scale (HDRS)[33], Clinical Global Impression Scale (CGI)[34] for measuring improvement in symptoms and remission rates
2. **Functioning:** Global assessment of functioning GAF[35]
3. **Duration of Untreated Psychosis:** Comprehensive Assessment of At-Risk Mental States (CAARMS) at baseline[36]
4. **Quality of life:** EuroQol-5[37]
5. **Family burden**: Family Burden Scale[38]
6. **Stigma**: Internalised Stigma of Mental Illness[39]
7. **Physical health**: weight and Body Mass Index
8. **Substance abuse**: Drug Abuse Screening Tool[40]
9. **Response to antipsychotic treatment:** Change in BPRS[31] from baseline to four weeks after starting antipsychotics.
10. **Adherence to antipsychotic treatment:** Medication Adherence Rating Scale (MARS)[41]
11. **Rates of disengagement** are defined as a participant's refusal to engage with treatment or not responding to contact requests for a consecutive 4-week period during follow-up while still residing within the study site catchment area.[42]
12. **Remission rates:** Improvement in psychotic symptoms from baseline (operationalised as a score of 3 or below on the Brief Psychiatric Rating Scale[31]) maintained for three months.
13. **Service utilisation metrics** measures will be used as a measure of the acceptability of the intervention. These will include patients screened for suspected psychosis out of all those presenting to at different contact points, the median time taken in screening, frequency and duration of contacts, the median length of time to treatment from assessment to starting treatment and the proportion of cases identified as At-Risk Mental State (ARMS) for FEP out of total accepted cases, the willingness of TSH to work with health services (indicated by the number of TSH approached, consented and completing training programme)

For peer review only - http://bmjopen.bmj.com/site/about/guidelines.xhtml

14. **The acceptability of trial procedures** will also be assessed through adherence to trial procedures and completion of the assessments at baseline and 12 weeks.
15. **Fidelity of intervention:** First-Episode psychosis services fidelity scale, modified and adapted for the local settings[43] will be used to assess the degree to which mental health teams deliver evidence-based care for FEP.
16. **Implementation costs:** The client Service Receipt Inventory[44] will measure direct and indirect costs in implementing the intervention to estimate health economic costs from the payer and provider perspective. We will estimate mean, median and quartile costs at each time point.

Table 1. Data Collection Schedule and List of Assessments

Description	Measure	Screening Assessment	Baseline Assessment	4 weeks post-baseline	3 Month Post baseline
Age	Date of birth	✓			
Gender	Male/female	✓			
Education	Years spent in education	✓			
Marital status		✓			
Work status		✓			
Psychopathology	Brief Psychiatric Rating Scale (BPRS)[31]		✓		✓
	Young Mania Rating Scale (YMRS)[32]		✓		✓

For peer review only - http://bmjopen.bmj.com/site/about/guidelines.xhtml

	Hamilton Depression Rating Scales (HDRS)[33]		✓		✓
	Clinical Global Impression Scale (CGI)[34]		✓		✓
Occupational and Social Functioning	Global Assessment of Functioning (GAF)[35]		✓		✓
Duration of untreated psychosis	Comprehensive Assessment of At-Risk Mental States (CAARMS)[36]	✓			
Quality of Life	EuroQol-5[37]		✓		✓
Family Burden	Perceived Family Burden Scale (PFBS)[38]		✓		✓
Perceived Stigma:	Internalized Stigma of Mental Illness (ISMI)[39]		✓		✓
Physical Health	Weight and Body Mass Index		✓		✓
Substance Abuse	Drug Abuse Screening Tool (DAST-10)[40]		✓		✓
Response to antipsychotics treatment	Brief Psychiatric Rating Scale (BPRS)[31]			✓	
Adherence to antipsychotic treatment	Medication Adherence Rating Scale (MARS)[41]				✓
Rates of disengagement	Response to contact requests for a consecutive 4-week period during follow-up				✓
Remission rates	Score of 3 or below on the Brief Psychiatric		✓		✓

For peer review only

	Rating Scale[31]) maintained at least for three months.				
Service utilisation metrics	indicated by;<br>- number of TSH approached, consented and completed the training programme.<br>- patients screened for suspected psychosis<br>-Total number of confirmed cases	✓	✓		✓
The acceptability of trial procedures	adherence to trial procedures and completion of the assessments at baseline and 12 weeks.		✓		✓
Fidelity of intervention:	First-Episode psychosis services fidelity scale, modified and adapted for the local settings[43]		✓		✓
Implementation costs:	The client Service Receipt Inventory[44]		✓		✓

For peer review only

For peer review only - http://bmjopen.bmj.com/site/about/guidelines.xhtml

Blinding and allocation concealment:

It is not possible to blind study participants from their treatment arm allocation. However, the assessment team conducting the baseline and follow-up assessments will be masked to the treatment arm of the cluster. During all assessments, the key feasibility outcome measures will be completed first to minimise the risk of bias in the event of unmasking, and if it occurs, the point of unmasking will be recorded. Sensitivity analyses will be carried out to assess the effect of unmasking on the clinical outcomes. The trial statistician will be blind to allocation status. The psychiatrist diagnosing FEP will be blind to the diagnosis given by PCPs and the impression of illness by TSHs. This will help to minimise the bias and provide a good measure of training intervention.

Qualitative Data Collection:

We will use semi-structured interviews after the trial follow-up to explore the views of participants and carers from both trial arms, physicians, traditional healers, and decision-makers with responsibilities for developing or implementing health policy in the directorate of health for future implementation and evaluation.   Around 4-6, separate interviews will be conducted with each group. The semi structured interviews will explore the (1) acceptability of educational materials and delivery, (2) the acceptability of evaluating THE HOPE in a definitive trial, (3) the burden of training, (4) barriers and facilitators to early detection and collaboration with mental health services and (5) overall satisfaction with the intervention. Participants for these interviews will be purposively sampled to stratify by gender and urban setting. A semi-structured interview topic guide will be used to facilitate the interviews.

Process evaluation:

We will conduct (i) Interviews with those receiving and delivering THE HOPE (assess acceptability) and (ii) Interviews with healthcare decision-makers (to support implementation) evaluation of training (pre- and post-training scores).

Withdrawal criteria:

There are no anticipated risks involved for those who decide to take part in this study. The study includes sharing personal experiences, which some may find uncomfortable. Participants are under

For peer review only

no obligation to answer any questions that make them feel uncomfortable. The information received from the participant will not be used in any way that might cause any harm. Participants will be informed that if there is any problem throughout the participation, the trained mental health professionals will be available for help.

Participants will be informed that they can withdraw from the study at any time without giving a reason and that their clinical care will not be affected. Participants will be withdrawn from the study in the event of any of the following (i) Non-compliance with the study protocol that results in a serious breach of protocol (GCP or other), which can compromise data integrity or significantly affect the scientific value of the reported results. (ii) A serious untoward incident compromising the patient's safety. Any data collected up to the point of discontinuation from the study will be reported as such.

Patients requiring inpatients inpatient admission or ECT (electroconvulsive therapy) or any other therapeutic procedure as part of the treatment or exacerbation of the symptoms will not be discontinued from the study, provided that the procedure is as advised by their treating psychiatrist and the patient continues to consent for the study.

In case of reported harm to the participants directly because of receiving the treatment or the control, the harms will be reported to the Data and Safety Monitoring Board (DSMB) and Trial Steering Committee (TSC), who will further advise if the trial needs to be stopped.

Study timeline:

We aim for the pilot RCT to start on 1st April 2023. Recruitment will commence when the trial commences and will be conducted till 1st April 2024; there will be a three-month follow-up after the recruitment of the last patient. We anticipate that the analysis will start in September 2024.

End of Trial:

The end of the study is defined as when the 12-week follow-up assessment and all qualitative interviews have been completed. Keele University ethics and the ethics board in the local context will be notified of the end of the study.

For peer review only - http://bmjopen.bmj.com/site/about/guidelines.xhtml

Data Management:

All data will be managed in line with the protocol and will be stored securely following the KMU and Keele University standard policy on data storage and confidentiality. Individual participant data will be pseudonymised through the use of a unique study ID and will be stored securely and separately from any identifiable data. Access to data will be restricted to members of the Keele and KMU Research Team and will be stored in accordance with SOPs. Outcome data will be entered into an electronic database which will be stored on an encrypted secure network at KMU, requiring a password to access. Data entry will be quality-checked and coded using standard processes.

Data Analysis:

Quantitative analysis:

Being a feasibility trial, the main analysis will focus on process outcomes and include estimation (with one- and two-sided 80% and 90% confidence interval estimation) of the number/proportion of potentially eligible patients, consent rate, overall uptake rate (in relation to number potentially eligible), average cluster sizes (and variation in cluster sizes), treatment fidelity – the proportion of patients having the protocolled intervention pathway (intervention arm), intervention acceptability, assessment of possible contamination effect across clusters, overall retention/follow-up rate of participants. Our point and interval estimates will be considered alongside target success criteria for informing overall feasibility and potential progress to a main RCT.

We will also calculate means and confidence intervals of clinical outcomes (both within- and between- study groups) and determine which outcome(s) is/are most sensitive to change, and evaluate parameters required in order to inform the sample size calculation for the main trial.

Due to the lack of similar studies in this area, it is not possible to suggest clear progression criteria to guide the decisions about the feasibility of proceeding to a definitive trial in future. We suggest following a priori progression criteria in order to guide the feasibility of a future definitive trial. In light of the following criteria, the trial findings will be presented to the steering committee, which will advise about the feasibility of proceeding to the definitive trial.

Red: i.e., the progression to a definitive trial using the proposed intervention and procedures is not feasible. Recruitment below 90 in total (45 in both arms) over 12 months period, less than 70% of

For peer review only - http://bmjopen.bmj.com/site/about/guidelines.xhtml

recruited sample completed all follow-up visits, and less than 70% of TSH in both arms consented, participated in the training, and worked with psychiatric services in referring the suspected cases.

Amber, i.e., the progression to a definitive trial using the proposed intervention is feasible but would require modifications in the intervention, recruitment or follow-up procedures. Recruitment of 90-130 participants in total over 12 months period, 70-80 % of recruited sample completed all follow-up visits, and 70-85% of TSH in both arms consented, participated in trainings, and worked with psychiatric services in referring the suspected cases.

Green, i.e. the progression to a definitive trial using the proposed intervention is feasible without changes in the intervention, recruitment or follow-up procedures.

Recruitment of more than 130 participants in total over 12 months period (averaging at least about 1.5 patients recruited per UC), more than 80% of the recruited sample completed all follow-up visits and >85% proportion of TSH in both arms consented, participated in trainings and worked with psychiatric services in referring the suspected cases.

Qualitative analysis:

An inductive, exploratory framework will be adopted using thematic analysis incorporating elements of grounded theory.[45] A sample of early transcripts will be independently coded by the research team, and a coding framework will be agreed upon. This framework will then be applied to subsequent coding. Independent researchers will analyse coded data to develop categories and themes. The constant comparison[46] will be used in the analysis to explore connections within and across transcripts and codes, highlighting consistencies and variation. The analysis will be an iterative process; emergent findings will be used to further refine topic guides for subsequent interviews.

Trial Management and Monitoring: The Project Management Committee for the HOPE study will be responsible for setting up, ongoing management and monitoring of the trial. The trial will be monitored in line with the protocol and the trial standard operating procedures. The standard operating procedure will be based on the recently concluded STOPS+ trial[47,] and the procedures will be audited by an independent team. An independent TSC, which will have service user and sponsor representatives, will monitor the trial progress. The TSC is empowered to independently review the

For peer review only - http://bmjopen.bmj.com/site/about/guidelines.xhtml

ethical and data management procedures. A DSMB will be convened to ensure the safety of participants and the integrity of the data.

Safety and Adverse events reporting:

The principal investigator and local site staff will be responsible for detecting, documenting, and reporting events that meet the definition of adverse events AE or serious adverse event SAE. Any potential AE or SAE reported by the participant, family member or identified by the trial staff will be reported to the supervising psychiatrist within 24 hours. It will be the responsibility of the supervising psychiatrist to assess the seriousness and causality, depending on the nature of the event reported. The relevant safety data will be reported to the principal Investigator and the Local Ethical Advisory and Review Board, according to the nature of the AE and the necessary reporting timeframe. AEs categorised as "serious" (SAE) will also be reported to Data Safety and Management Board (DSMB) and TSC within 15 days, whereas non-serious AEs will be 'bundled' and documented in the annual summary sent to the regulatory authority if required.

Ethics and Dissemination:

This study has obtained ethical approval from Keele University Ethical Review Panel (ref: MH210177), Khyber Medical University Ethical Review Board (ref: DIR/KMU-EB/IG/001005) and National Bioethics Committee Pakistan (Ref: No.4-87/NBC-840/22/621).

Any amendments to the protocol will not be implemented prior to receipt of the required approvals. This does not affect the individual clinician's responsibility to take immediate action if thought necessary to protect the health and interest of individual participants. The Chief Investigator/Sponsor will ensure that the main REC is notified that the trial has finished within 90 days after the end of the trial. If the trial is terminated prematurely, those reports will be made within 15 days after the end of the trial.

Study results will be shared with local communities, service users, traditional healers and health administrators. Throughout the study period, we will hold several information-sharing and dissemination meetings involving different stakeholders to discuss the findings from different phases of the study and trial findings. We will also produce training manuals for healthcare professionals and TSHs, and these will be shared widely with relevant local and international health and policy agencies. The study findings will also be presented in local and international research and health-

For peer review only - http://bmjopen.bmj.com/site/about/guidelines.xhtml

service research. At least three publications are planned, which will be published in open-access resources. Manuscripts with the results of the pilot trial will be submitted for publication in peer-reviewed journals and presented at academic conferences. Trial results will be disseminated to patients with a summary sheet outlining the trial findings in lay language. We will also disseminate findings through social media such as Twitter accounts. Participant-level dataset will be available after trial completion on request for academic use, in line with patient confidentiality and anonymity guidelines.

Discussion:

In many LMICs, psychiatric disorders are considered mainly as a result of a social imbalance or to have spiritual causes, and these beliefs are also prevalent in immigrants living in high-income countries. [48, 9] These alternate explanations of disease, among other factors, alter the help-seeking behaviour of patients and their pathway to care, which seems to be of great importance in psychotic disorders.[49] A recent qualitative study highlighted the role of alternative medicine and stigma in DUP in an LMIC setting. It was reported that patients and families had difficulty recognising symptoms as an illness and would attribute these to supernatural causes, resulting in a delay before contacting a GP or psychiatrist. Similarly, a systematic review concluded that a large proportion of patients in LMICs use TSHs as their first point of contact for accessing care associated with longer DUP. The review recommended that services in these countries need to focus on collaborative working with TSH to facilitate access to biomedical care.[50]

There is almost no literature on implementation research involving traditional healers in the care of psychosis. This study aims to include TSHs and primary care in the treatment pathways for improving the care of FEP. THE HOPE will make a significant contribution to the study of working collaboratively with traditional healers. This will have a significant impact on developing new ways of working with traditional healers and evaluating these using the randomised controlled trial.

The proposed research has three major innovations: developing intervention by using ethnographic and qualitative methods to understand the context and the working of TSH; improving the skills of primary care and TSH by using a well-structured education and training programme and using a task sharing and task shifting approach involving psychiatrists, primary care and traditional healers. We also include a complete process evaluation of the study after the trial is completed.

For peer review only - http://bmjopen.bmj.com/site/about/guidelines.xhtml

The results of this study and process evaluation will inform the planning and conduct of a full-scale pragmatic RCT**.** At present, it is not possible to conduct an adequately powered trial of any intervention with TSHs in these settings, as the feasibility and acceptability of such an intervention has not been studied.

Developing a potential model that can be implemented with the help of traditional healers will have a wide economic and social impact and is likely to be highly acceptable to those using these services. By promoting active partnership between patients, members of the public and researchers, we will maximise impact by recognising and supporting the unique roles service users have in research prioritisation, design and management, data collection and the analysis and dissemination of findings.

The integration of mental health and primary care services is advocated in developed as well as developing countries[51,52]. The detection and management of FEP in primary care is a major challenge and is practically lacking in most LMICs, and evidence suggests the importance of training GPs to improve the early detection and management of FEP to reduce the delay between the onset of symptoms and initiation of treatment[53]. The proposed intervention involves primary care physicians in training the TSH. Involving PCPs in The HOPE intervention and cascade model of training will help to involve the primary care in the treatment of FEP and will help to reduce the duration of untreated psychosis and build capacity in primary care.

Limitations of the Proposed Study

To the best of our knowledge, no study prior to this has attempted collaborating with TSHs to integrate mental health and primary care services to provide early intervention in untreated psychosis and evaluate the model in a feasibility randomised controlled trial. We use a mixed methods approach, comprising both quantitative and qualitative methods, to develop, test and evaluate the feasibility of the intervention and understand its strengths and limitations.

A major limitation of our study is the small sample size. As discussed in the methods, the sample size is aimed to evaluate the acceptability and feasibility of the intervention, but the study is not adequately powered to test the clinical parameters. Contamination between the intervention and control arm is possible, considering that the participants in the intervention and the control arm may

For peer review only - http://bmjopen.bmj.com/site/about/guidelines.xhtml

interact within the community. We propose measures to implement the masking of assessors to the trial arms. However, it may not be possible to maintain the assessors' blindness to the intervention or control arm.

For peer review only

For peer review only - http://bmjopen.bmj.com/site/about/guidelines.xhtml

Footnotes

Acknowledgements: The author would like to thank Dr Tom Kingstone, Keele University, for providing valuable suggestions regarding qualitative methods. We also want to thank the service user representative, Mr Sher Dil Khan, for his valuable contribution to the design and oversight of the study as part of the Trial Steering Committee. We will also like to express our gratitude to Keele Patient and Public Involvement and Engagement (PPIE) group and service users' group in Peshawar, Pakistan, for their useful suggestions in the study design. We would also like to thank the Office of Research, Innovation, and Commercialization (ORIC) at Khyber Medical University KMU Pakistan for supporting the study set-up and obtaining the ethics and governance approvals.

Author Contributions: SF wrote the grant application and obtained funding. SF is the chief investigator and designed the methodology and wrote the initial draft. ML provided the statistical expertise and contributed to designing the study and methodological details of the RCT, wrote the analysis plan and economic evaluation part of the protocol and contributed significantly to obtaining the funding. LD and NS contributed to designing and developing the qualitative and anthropological studies methodology for intervention development and process evaluation. SF, LD, MMA, AK, MA, JA, IC, NH, MG, MI, AM, CM, ML and MFK obtained the funding for the study, conceived, and developed the intervention and study design, and led the team's creation. GA, SS, JA, MI and PK contributed to writing the ethics application, developing and designing participants' information sheets and consent forms, submitting l ethics applications, obtaining ethics and governance approvals, and submitting the article. IA, SSMU and SH contributed to patient and public involvement, community engagement and setting up of the study sites. The initial and final manuscript has been read, revised, and approved by all named authors, and no other persons have satisfied the authorship criteria but are not listed.

Funding Statement: This study is funded by Medical Research Council (MRC) UK grant number (MR/T040378/1). The funder has no role in designing and reporting the study. An independent Trial Steering Committee (TSC), with service user and sponsor representatives, will monitor the trial progress.

For peer review only - http://bmjopen.bmj.com/site/about/guidelines.xhtml

Conflict of Interest: None declared.

For peer review only

References:

1. Kelleher I, Connor D, Clarke MC, Devlin N, Harley M, Cannon M. Prevalence of psychotic symptoms in childhood and adolescence: a systematic review and meta-analysis of population-based studies. Psychological medicine. 2012 Sep;42(9):1857-63. doi:10.1017/S0033291711002960

2. Gerstenberg M, Theodoridou A, Traber-Walker N, Franscini M, Wotruba D, Metzler S, Müller M, Dvorsky D, Correll CU, Walitza S, Rössler W. Adolescents and adults at clinical high-risk for psychosis: age-related differences in attenuated positive symptoms syndrome prevalence and entanglement with basic symptoms. Psychological medicine. 2016 Apr;46(5):1069-78. doi:10.1017/S0033291715002627

3. Sullivan SA, Kounali D, Cannon M, David AS, Fletcher PC, Holmans P, Jones H, Jones PB, Linden DE, Lewis G, Owen MJ. A population-based cohort study examining the incidence and impact of psychotic experiences from childhood to adulthood, and prediction of psychotic disorder. American Journal of Psychiatry. 2020 Apr 1;177(4):308-17. doi: 10.1176/appi.ajp.2019.19060654

4. Farooq S, Large M, Nielssen O, Waheed W. The relationship between the duration of untreated psychosis and outcome in low-and-middle income countries: a systematic review and meta-analysis. Schizophrenia research. 2009 Apr 1;109(1-3):15-23. doi: 10.1016/j.schres.2009.01.008

5. Lora A, Kohn R, Levav I, McBain R, Morris J, Saxena S. Service availability and utilisation and treatment gap for schizophrenic disorders: a survey in 50 low-and middle-income countries. Bulletin of the World Health Organization. 2012 Jan;90(1):47-54B. doi:10.2471/BLT.11.089284

6. De Silva MJ, Lee L, Fuhr DC, Rathod S, Chisholm D, Schellenberg J, Patel V. Estimating the coverage of mental health programmes: a systematic review. International journal of epidemiology. 2014 Apr 1;43(2):341-53. doi:10.1093/ije/dyt191

7. Healy C, Brannigan R, Dooley N, Coughlan H, Clarke M, Kelleher I, Cannon M. Childhood and adolescent psychotic experiences and risk of mental disorder: a systematic review and meta-analysis. Psychological Medicine. 2019 Jul;49(10):1589-99. doi:10.1017/S0033291719000485

8. Burns JK, Tomita A. Traditional and religious healers in the pathway to care for people with mental disorders in Africa: a systematic review and meta-analysis. Social psychiatry and psychiatric epidemiology. 2015 Jun;50(6):867-77. doi:10.1007/s00127-014-0989-7

9. Bhikha AG, Farooq S, Chaudhry N, Husain N. A systematic review of explanatory models of illness for psychosis in developing countries. International Review of Psychiatry. 2012 Oct 1;24(5):450-62. doi:10.3109/09540261.2012.711746

10. Khattak MI, Dikomitis L, Khan MF, Haq MU, Saeed U, Awan NR, Haq ZU, Shepherd T, Mallen CD, Farooq S. Patients' and healthcare professionals' perspectives on a community-based intervention for schizophrenia in Pakistan: A focus group study. Plos one. 2022 Aug 29;17(8):e0273286. doi:10.1371/journal.pone.0273286

11. Saeed K, Gater R, Hussain A, Mubbashar M. The prevalence, classification and treatment of mental disorders among attenders of native faith healers in rural Pakistan. Social psychiatry and psychiatric epidemiology. 2000 Oct;35(10):480-5.

For peer review only - http://bmjopen.bmj.com/site/about/guidelines.xhtml

12. Qidwai W. Use of services of spiritual healers among patients presenting to family physicians at a teaching hospital in Karachi, Pakistan. Pakistan Journal of Medical Sciences. 2003;19(1):52.

13. Mubbashar MH, Saeed K. Development of mental health services in Pakistan. EMHJ-Eastern Mediterranean Health Journal, 7 (3), 392-396, 2001. 2001.

14. Irfan M, Caldas de Almeida JM, Irfan UM, Raza UA, Farooq S. Schizophrenia diagnosis and treatment by general practitioners: A cross-sectional study in district Peshawar, Pakistan. JPMA J Pak Med Assoc. 2015 Sep 1;65(9):937-42.

15. McDaid D, Park AL, Iemmi V, Adelaja B, Knapp M. Growth in the use of early intervention for psychosis services: An opportunity to promote recovery amid concerns on health care sustainability.

16. Homsy J, King R, Balaba D, Kabatesi D. Traditional health practitioners are key to scaling up comprehensive care for HIV/AIDS in sub-Saharan Africa. Aids. 2004 Aug 20;18(12):1723-5. doi: 10.1097/01.aids.0000131380.30479.16

17. Nortje G, Oladeji B, Gureje O, Seedat S. Effectiveness of traditional healers in treating mental disorders: a systematic review. The Lancet Psychiatry. 2016 Feb 1;3(2):154-70. doi:10.1016/S2215-0366(15)00515-5

18. Veling, W., Burns, J. K., Makhathini, E. M., Mtshemla, S., Nene, S., Shabalala, S., ... & Susser, E. (2019). Identification of patients with recent-onset psychosis in KwaZulu Natal, South Africa: a pilot study with traditional health practitioners and diagnostic instruments. Social psychiatry and psychiatric epidemiology, 54(3), 303-312. doi:10.1007/s00127-018-1623-x

19. Skivington K, Matthews L, Simpson SA, Craig P, Baird J, Blazeby JM, Boyd KA, Craig N, French DP, McIntosh E, Petticrew M. A new framework for developing and evaluating complex interventions: update of Medical Research Council guidance. bmj. 2021 Sep 30;374. doi:10.1136/bmj.n2061

20. Eldridge SM, Chan CL, Campbell MJ, Bond CM, Hopewell S, Thabane L, Lancaster GA. CONSORT 2010 statement: extension to randomised pilot and feasibility trials. bmj. 2016 Oct 24;355. | doi: 10.1136/bmj.i5239

21. Eldridge SM, Costelloe CE, Kahan BC, Lancaster GA, Kerry SM. How big should the pilot study for my cluster randomised trial be?. Statistical methods in medical research. 2016 Jun;25(3):1039-56. doi: 10.1177/0962280215588242

22. Jongsma HE, Turner C, Kirkbride JB, Jones PB. International incidence of psychotic disorders, 2002–17: a systematic review and meta-analysis. The Lancet Public Health. 2019 May 1;4(5):e229-44. doi:10.1016/S2468-2667(19)30056-8

23. Julious SA. Pilot studies in clinical research. Statistical Methods in Medical Research. 2016 Jun;25(3):995-6. doi: 10.1177/0962280216651022

24. National Institute of Health and Clinical Sciences NICE Psychosis and schizophrenia in children and young people: recognition and management. Last updated: 26 October 2016, available at https://www.nice.org.uk/guidance/cg155.

25. Early Psychosis Guidelines Working Group. Australian clinical guidelines for early psychosis. Orygen The National Centre of Excellence in Youth Mental Health: Melbourne, Australia. 2016. doi:10.1007/s00127-015-1140-0

For peer review only - http://bmjopen.bmj.com/site/about/guidelines.xhtml

26. mhGAP training manuals for the mhGAP Intervention Guide for mental, neurological and substance use disorders in non-specialised health settings version 2.0 (for eld testing). Geneva: World Health Organization; 2017 (WHO/MSD/MER/17.6). Licence: CC BY-NC-SA 3.0 IGO. https://www.who.int/mental_health/mhgap/training_manuals/en/

27. Rahman A, Hamdani SU, Awan NR, Bryant RA, Dawson KS, Khan MF, Azeemi MM, Akhtar P, Nazir H, Chiumento A, Sijbrandij M. Effect of a multicomponent behavioral intervention in adults impaired by psychological distress in a conflict-affected area of Pakistan: a randomised clinical trial. Jama. 2016 Dec 27;316(24):2609-17. doi:10.1001/jama.2016.17165

28. Breitborde NJ, Srihari VH, Woods SW. Review of the operational definition for first-episode psychosis. Early intervention in psychiatry. 2009 Nov;3(4):259-65. doi:10.1111/j.1751-7893.2009.00148.x

29. Farooq S, Taylor M. Clozapine: dangerous orphan or neglected friend? The British Journal of Psychiatry. 2011 Apr;198(4):247-9. doi: 10.1192/bjp.bp.110.088690

30. Jakobsen KD, Frederiksen JN, Hansen T, Jansson LB, Parnas J, Werge T. Reliability of clinical ICD-10 schizophrenia diagnoses. Nordic journal of psychiatry. 2005 Jun 1;59(3):209-12. doi: 10.1080/08039480510027698

31. Overall JE, Gorham DR. The brief psychiatric rating scale. Psychological reports. 1962 Jun;10(3):799-812.

32. Young RC, Biggs JT, Ziegler VE, Meyer DA. Young mania rating scale. Journal of Affective Disorders. 2000. doi: 10.1037/t20936-000

33. Williams JB. A structured interview guide for the Hamilton Depression Rating Scale. Archives of general psychiatry. 1988 Aug 1;45(8):742-7. doi:10.1001/archpsyc.1988.01800320058007

34. Haro JM, Kamath SA, Ochoa SO, Novick D, Rele K, Fargas A, Rodriguez MJ, Rele R, Orta J, Kharbeng A, Araya S. The Clinical Global Impression–Schizophrenia scale: a simple instrument to measure the diversity of symptoms present in schizophrenia. Acta Psychiatrica Scandinavica. 2003 May;107:16-23. doi: 10.1034/j.1600-0447.107.s416.5.x.

35. Hall RC. Global assessment of functioning: a modified scale. Psychosomatics. 1995 May 1;36(3):267-75. doi: 10.1016/S0033-3182(95)71666-8

36. Yung A, Phillips L, McGorry P, Ward J, Donovan K, Thompson K. Comprehensive assessment of at-risk mental states (CAARMS). Melbourne, Australia, University of Melbourne, Department of Psychiatry, Personal Assessment and Crisis Evaluation Clinic. 2002 Jan.

37. Group TE. EuroQol-a new facility for the measurement of health-related quality of life. Health policy. 1990 Dec 1;16(3):199-208. doi: 10.1016/0168-8510(90)90421-9

38. Pai S, Kapur RL. The burden on the family of a psychiatric patient: development of an interview schedule. The British Journal of Psychiatry. 1981 Apr;138(4):332-5. doi: 10.1192/bjp.138.4.332

39. Lysaker PH, Davis LW, Warman DM, Strasburger A, Beattie N. Stigma, social function and symptoms in schizophrenia and schizoaffective disorder: Associations across 6 months. Psychiatry research. 2007 Jan 15;149(1-3):89-95. doi: 10.1016/j.psychres.2006.03.007

40. Skinner HA. The drug abuse screening test. Addictive behaviors. 1982 Jan 1;7(4):363-71.

For peer review only - http://bmjopen.bmj.com/site/about/guidelines.xhtml

For peer review only

41. Thompson K, Kulkarni J, Sergejew AA. Reliability and validity of a new Medication Adherence Rating Scale (MARS) for the psychoses. Schizophrenia research. 2000 May 5;42(3):241-7. doi:10.1016/S0920-9964(99)00130-9

42. Tait L, Birchwood MA, Trower P. Predicting engagement with services for psychosis: insight, symptoms and recovery style. The British Journal of Psychiatry. 2003 Feb;182(2):123-8. doi: 10.1192/bjp.182.2.123

43. Addington DE. First Episode Psychosis Services Fidelity Scale (FEPS-FS 1.0) and Manual. University of Calgary Press; 2021.

44. Knapp MR, Knudsen HC, Amaddeo F, Gaite L, van Wijngaarden B. Client Socio-Demographic and Service Receipt Inventory-European Version: Development of an instrument for international research-EPSILON Study 5. British Journal of Psychiatry. 2000;177:S28-33. doi: 10.1192/bjp.177.39.s28

45. Glaser BG, Strauss A. The discovery of grounded theory: theories for qualitative research.1967

46. Corbin JM, Strauss A. Grounded theory research: Procedures, canons, and evaluative criteria. Qualitative sociology. 1990 Mar;13(1):3-21. doi: 10.1007/BF00988593

47. Shepherd TA, Ul-Haq Z, Ul-Haq M, et al Supervised treatment in outpatients for schizophrenia plus (STOPS+): protocol for a cluster randomised trial of a community-based intervention to improve treatment adherence and reduce the treatment gap for schizophrenia in Pakistan BMJ Open 2020;10:e034709. doi: 10.1136/bmjopen-2019-034709

48. Awan NR, Jehangir SF, Irfan M, Naeem F, Farooq S. Explanatory model of illness of the patients with schizophrenia and the role of educational intervention. Schizophrenia Research. 2017 Dec 1;190:68-73. doi: 10.1016/j.schres.2017.03.006

49. Bhikha A, Farooq S, Chaudhry N, Naeem F, Husain N. Explanatory models of psychosis amongst British South Asians. Asian journal of psychiatry. 2015 Aug 1;16:48-54. doi: 10.1016/j.ajp.2015.05.042

50. Lilford P, Rajapakshe OB, Singh SP. A systematic review of care pathways for psychosis in low-and middle-income countries. Asian Journal of Psychiatry. 2020 Dec 1;54:102237. doi: 10.1016/j.ajp.2020.102237

51. Jacob KS. Community care for people with mental disorders in developing countries: problems and possible solutions. The British Journal of Psychiatry. 2001 Apr;178(4):296-8.doi: 10.1192/bjp.178.4.296

52. World Health Organization. Mental health in primary care: illusion or inclusion? World Health Organization; 2018.

53. Lester H, Birchwood M, Freemantle N, Michail M, Tait L. REDIRECT: cluster randomised controlled trial of GP training in first-episode psychosis. British Journal of General Practice. 2009 Jun 1;59(563):e183-90. doi: 10.3399/bjgp09X420851

For peer review only - http://bmjopen.bmj.com/site/about/guidelines.xhtml

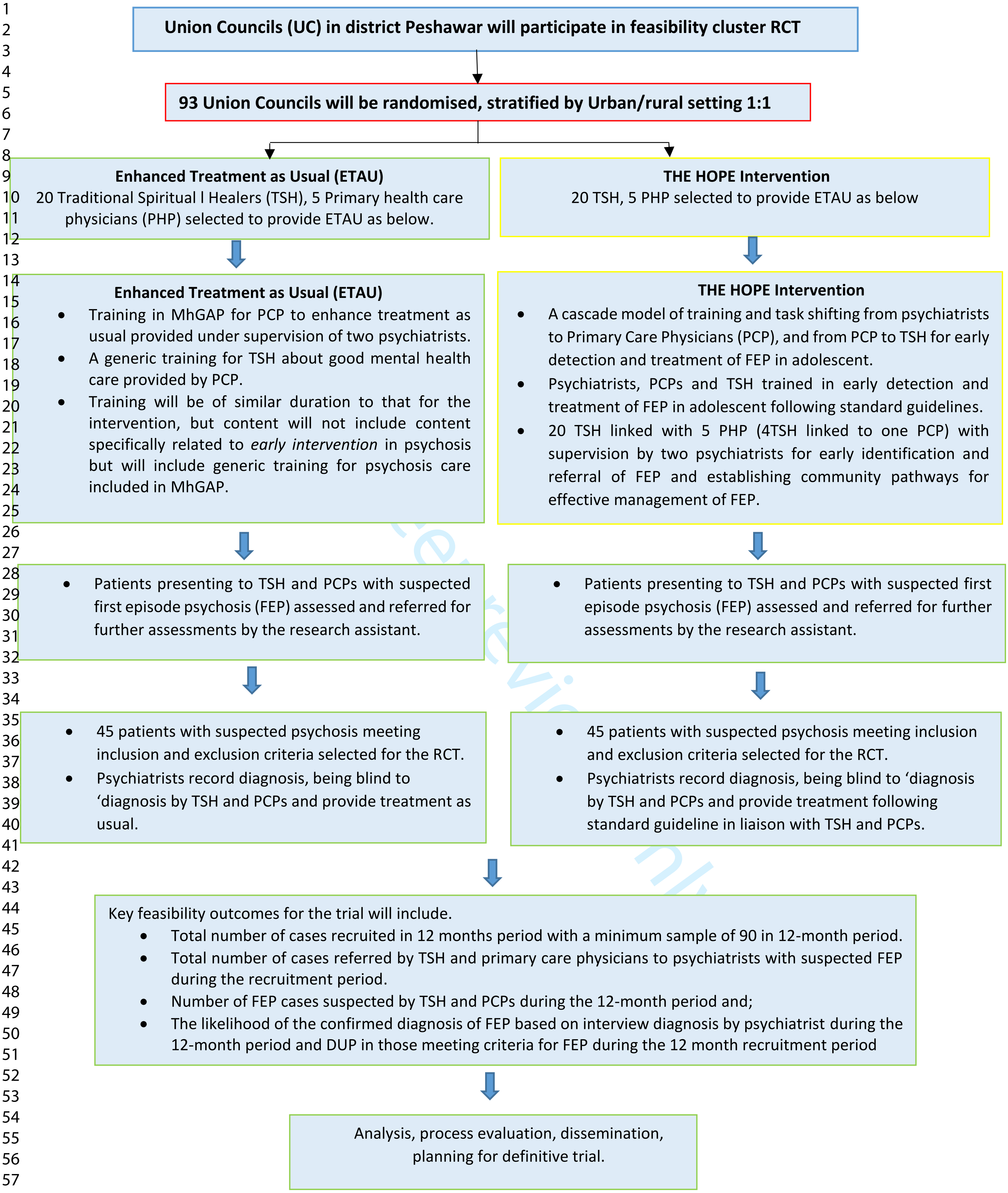

Figure 1: The HOPE Flow Diagram

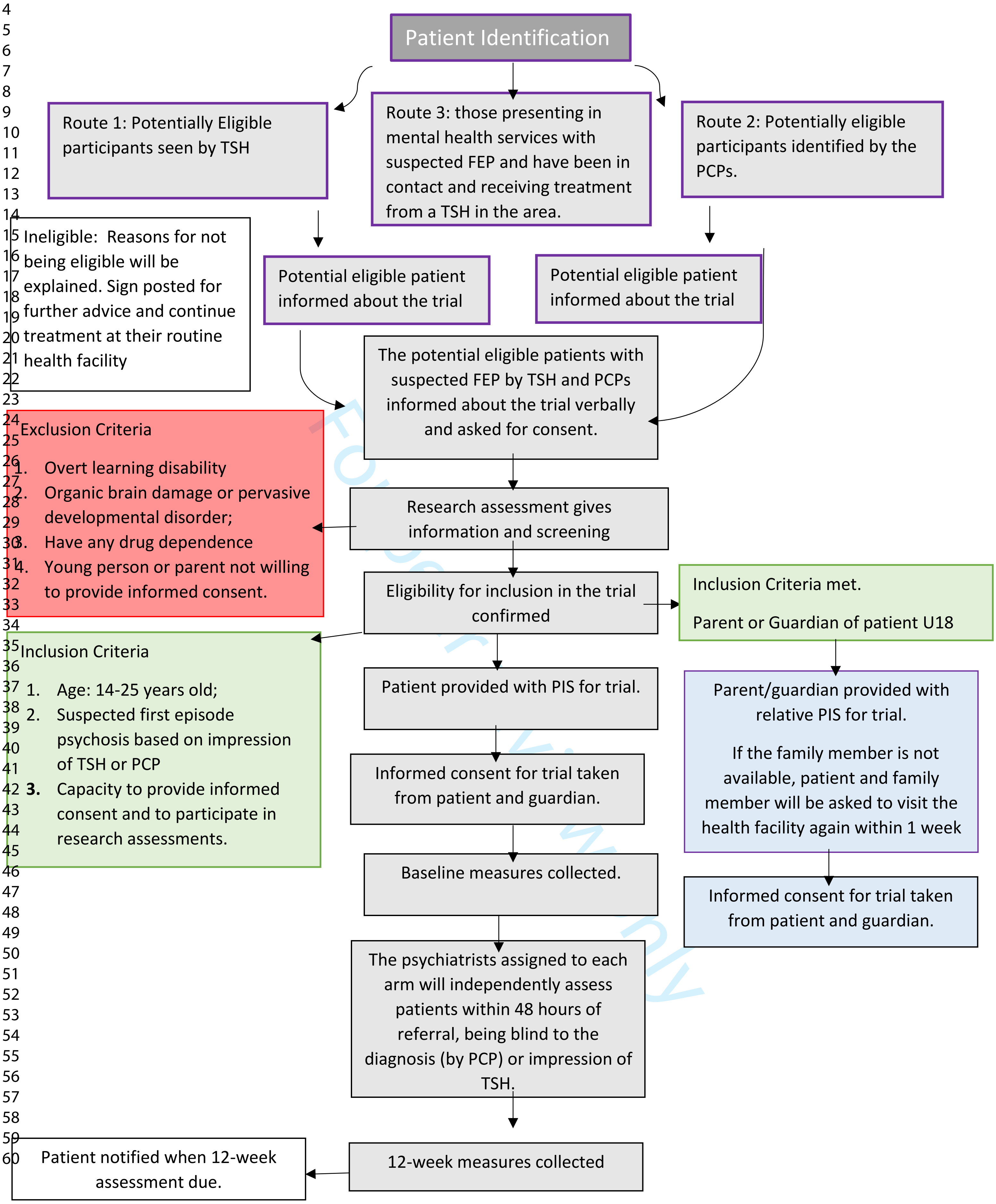

Figure 2. Patient flow and assessments in THE HOPE trial

For peer review only

Appendices

Appendix 1. Participant Consent Form – Young Person 16+

Participant Identification Number:

Participant Consent Form –

Young Person 16+

Study title: Traditional healers working with primary care and mental health for early intervention in psychosis in adolescent: intervention development and feasibility study

This form should be initialled and signed unless the young person is unable to read and write. If they cannot write then the form should be read aloud to the young person and they should be asked to mark the form in the relevant places.

	Please initial/mark box if you agree with the statements
1. I confirm that I have read and understood the information sheet dated 05/Mar/2021 (Version 1.0) for the above study. I have had the opportunity to consider the information, ask questions and have had these answered satisfactorily.	☐
2. I understand that participating in the THEHOPE study involves:<br>- Attending appointments with the research team. This includes; research assistants, primary care physicians, spiritual healers and psychiatrists.<br>- Attending appointments with clinicians where my mental health will be assessed<br>- Receiving treatment for my mental health condition if it is appropriate	☐
3. I understand that my participation is voluntary and that I am free to withdraw at any time without giving any reason and without my legal or medical rights being affected.	☐

For peer review only

4. I understand that all information and data collected during the course of the research will be kept strictly confidential and that any personal data e.g. name and address will only be used for the THEHOPE study. ☐

5. I understand that my data will be pseudonymised through the use of a unique study code. ☐

6. I agree that if the researcher is worried about my safety they may have to tell my doctor or other clinician about things I have told them. ☐

Please continue to next page

7. I understand that I may be invited to take part in research relating to THEHOPE in the future. ☐

8. I voluntarily agree to take part in this study. ☐

________________________________	____________________________	__________________
Name of Participant	Date	Signature or mark
________________________________	____________________________	__________________
Name of Researcher taking consent	Date	Signature

1 copy for participant and 1 copy for researcher.

For peer review only

Appendix 2. Participant Consent Form – Parent or Carer

Participant Identification Number:

Participant Consent Form – Parent or Carer

Study title: Traditional healers working with primary care and mental health for early intervention in psychosis in adolescent: intervention development and feasibility study

This consent form is intended for the parents or caregivers of the young person participating in the study.
This form should be initialled and signed unless the parent or carer is unable to read and write.

If they cannot write then the form should be read aloud and they should be asked to mark the form in the relevant places. This consent procedure must be witnessed by a trusted member of the family or community who is independent to the project. It is necessary that this individual then signs the form at the bottom in the indicated place.

Please initial/mark box if you agree with the statements

9. I confirm that I have read and understood the information sheet dated 05/Mar/2021 (Version 1.0) for the above study. I have had the opportunity to consider the information, ask questions and have had these answered satisfactorily. ☐ ☐

10. I understand that participation of **my child / young person I care for** in the THEHOPE study involves:
    - **My child / young person I care for** attending appointments with the research team. This includes; research assistants, primary care physicians, spiritual healers and psychiatrists.

For peer review only

- Attending appointments with clinicians where **my child / young person I care for** will be assessed for their mental health condition
- **My child / young person I care for** receiving treatment for their mental health condition if it is appropriate

11. I understand that participation of **my child / young person I care for** is voluntary and that we are free to withdraw at any time without giving any reason and without our legal or medical rights being affected. The information I have provided up to that point will be included for research purposes unless I state otherwise. After publication, my information cannot be withdrawn. ☐

Please continue to next page

12. I understand that all information and data collected during the course of the research will be kept strictly confidential and that any personal data e.g. name and address will only be used for the THEHOPE study. ☐

13. I understand that data collected from **my child / young person I care for** will be pseudonymised through the use of a unique study code. ☐

14. I understand and agree that should there be any concern for the safety and welfare of **my child/young person I care for** that the research team may wish to contact their doctor or other clinician. ☐

15. I understand that **my child / young person I care for** may be invited to take part in research relating to THEHOPE in the future. ☐

16. I voluntarily agree for **my child / young person I care for** to take part in this study. ☐

________________________	____________________	______________
Name of Parent / Carer	Date	Signature or Mark
________________________	____________________	______________
Name of Researcher	Date	Signature

For peer review only

taking consent

If parent or carer cannot write:

_________________________ ______________________ _______________________

Name and role of trusted observer	Date	Signature

1 copy for participant and 1 copy for researcher.

For peer review only

Reporting checklist for protocol of a clinical trial.

Based on the SPIRIT guidelines.

Instructions to authors

Complete this checklist by entering the page numbers from your manuscript where readers will find each of the items listed below.

Your article may not currently address all the items on the checklist. Please modify your text to include the missing information. If you are certain that an item does not apply, please write "n/a" and provide a short explanation.

Upload your completed checklist as an extra file when you submit to a journal.

In your methods section, say that you used the SPIRIT reporting guidelines, and cite them as:

Chan A-W, Tetzlaff JM, Gøtzsche PC, Altman DG, Mann H, Berlin J, Dickersin K, Hróbjartsson A, Schulz KF, Parulekar WR, Krleža-Jerić K, Laupacis A, Moher D. SPIRIT 2013 Explanation and Elaboration: Guidance for protocols of clinical trials. BMJ. 2013;346:e7586

		Reporting Item	Page Number
**Administrative information**			
Title	#1	Descriptive title identifying the study design, population, interventions, and, if applicable, trial acronym	1
Trial registration	#2a	Trial identifier and registry name. If not yet registered, name of intended registry	1
Trial registration: data set	#2b	All items from the World Health Organization Trial Registration Data Set	NA. Available on trial registry record
Protocol version	#3	Date and version identifier	1
Funding	#4	Sources and types of financial, material, and other support	23

For peer review only

Roles and responsibilities: contributorship	#5a	Names, affiliations, and roles of protocol contributors	1-3,23
Roles and responsibilities: sponsor contact information	#5b	Name and contact information for the trial sponsor	23
Roles and responsibilities: sponsor and funder	#5c	Role of study sponsor and funders, if any, in study design; collection, management, analysis, and interpretation of data; writing of the report; and the decision to submit the report for publication, including whether they will have ultimate authority over any of these activities	23
Roles and responsibilities: committees	#5d	Composition, roles, and responsibilities of the coordinating centre, steering committee, endpoint adjudication committee, data management team, and other individuals or groups overseeing the trial, if applicable (see Item 21a for data monitoring committee)	19
**Introduction**			
Background and rationale	#6a	Description of research question and justification for undertaking the trial, including summary of relevant studies (published and unpublished) examining benefits and harms for each intervention	5-6
Background and rationale: choice of comparators	#6b	Explanation for choice of comparators	5-6
Objectives	#7	Specific objectives or hypotheses	6-7
Trial design	#8	Description of trial design including type of trial (eg, parallel group, crossover, factorial,	7

For peer review only

		single group), allocation ratio, and framework (eg, superiority, equivalence, non-inferiority, exploratory)	
**Methods: Participants, interventions, and outcomes**			
Study setting	#9	Description of study settings (eg, community clinic, academic hospital) and list of countries where data will be collected. Reference to where list of study sites can be obtained	8
Eligibility criteria	#10	Inclusion and exclusion criteria for participants. If applicable, eligibility criteria for study centres and individuals who will perform the interventions (eg, surgeons, psychotherapists)	10-11
Interventions: description	#11a	Interventions for each group with sufficient detail to allow replication, including how and when they will be administered	8-10
Interventions: modifications	#11b	Criteria for discontinuing or modifying allocated interventions for a given trial participant (eg, drug dose change in response to harms, participant request, or improving / worsening disease)	16-17
Interventions: adherence	#11c	Strategies to improve adherence to intervention protocols, and any procedures for monitoring adherence (eg, drug tablet return; laboratory tests)	14-15
Interventions: concomitant care	#11d	Relevant concomitant care and interventions that are permitted or prohibited during the trial	NA. Does not apply
Outcomes	#12	Primary, secondary, and other outcomes, including the specific measurement variable	12-15

For peer review only

		(eg, systolic blood pressure), analysis metric (eg, change from baseline, final value, time to event), method of aggregation (eg, median, proportion), and time point for each outcome. Explanation of the clinical relevance of chosen efficacy and harm outcomes is strongly recommended	
Participant timeline	#13	Time schedule of enrolment, interventions (including any run-ins and washouts), assessments, and visits for participants. A schematic diagram is highly recommended (see Figure)	13-14, 17
Sample size	#14	Estimated number of participants needed to achieve study objectives and how it was determined, including clinical and statistical assumptions supporting any sample size calculations	8
Recruitment	#15	Strategies for achieving adequate participant enrolment to reach target sample size	11
**Methods: Assignment of interventions (for controlled trials)**			
Allocation: sequence generation	#16a	Method of generating the allocation sequence (eg, computer-generated random numbers), and list of any factors for stratification. To reduce predictability of a random sequence, details of any planned restriction (eg, blocking) should be provided in a separate document that is unavailable to those who enrol participants or assign interventions	8

For peer review only

Allocation concealment mechanism	#16b	Mechanism of implementing the allocation sequence (eg, central telephone; sequentially numbered, opaque, sealed envelopes), describing any steps to conceal the sequence until interventions are assigned	NA (Cluster RCT)
Allocation: implementation	#16c	Who will generate the allocation sequence, who will enrol participants, and who will assign participants to interventions	11,12 Allocation sequence generation is not relevant (cluster RCT).
Blinding (masking)	#17a	Who will be blinded after assignment to interventions (eg, trial participants, care providers, outcome assessors, data analysts), and how	16
Blinding (masking): emergency unblinding	#17b	If blinded, circumstances under which unblinding is permissible, and procedure for revealing a participant's allocated intervention during the trial	16
**Methods: Data collection, management, and analysis**			
Data collection plan	#18a	Plans for assessment and collection of outcome, baseline, and other trial data, including any related processes to promote data quality (eg, duplicate measurements, training of assessors) and a description of study instruments (eg, questionnaires, laboratory tests) along with their reliability and validity, if known. Reference to where data collection forms can be found, if not in the protocol	11-16
Data collection plan: retention	#18b	Plans to promote participant retention and complete follow-up, including list of any outcome data to be collected for participants	NA. No specific plans to collect data from patients who discontinue and deviations

For peer review only

		who discontinue or deviate from intervention protocols	from the protocol will be reported to DSMB and TSC.
Data management	#19	Plans for data entry, coding, security, and storage, including any related processes to promote data quality (eg, double data entry; range checks for data values). Reference to where details of data management procedures can be found, if not in the protocol	16-17
Statistics: outcomes	#20a	Statistical methods for analysing primary and secondary outcomes. Reference to where other details of the statistical analysis plan can be found, if not in the protocol	18-19
Statistics: additional analyses	#20b	Methods for any additional analyses (eg, subgroup and adjusted analyses)	18-19
Statistics: analysis population and missing data	#20c	Definition of analysis population relating to protocol non-adherence (eg, as randomised analysis), and any statistical methods to handle missing data (eg, multiple imputation)	NA. feasibility trial
**Methods: Monitoring**			
Data monitoring: formal committee	#21a	Composition of data monitoring committee (DMC); summary of its role and reporting structure; statement of whether it is independent from the sponsor and competing interests; and reference to where further details about its charter can be found, if not in the protocol. Alternatively, an explanation of why a DMC is not needed	19
Data monitoring: interim analysis	#21b	Description of any interim analyses and stopping guidelines, including who will have access to these interim results and make the final decision to terminate the trial	NA. No interim analyses planned, in case of harm reported refer to pg 17.

For peer review only

Harms	#22	Plans for collecting, assessing, reporting, and managing solicited and spontaneously reported adverse events and other unintended effects of trial interventions or trial conduct	19,20
Auditing	#23	Frequency and procedures for auditing trial conduct, if any, and whether the process will be independent from investigators and the sponsor	19
**Ethics and dissemination**			
Research ethics approval	#24	Plans for seeking research ethics committee / institutional review board (REC / IRB) approval	19
Protocol amendments	#25	Plans for communicating important protocol modifications (eg, changes to eligibility criteria, outcomes, analyses) to relevant parties (eg, investigators, REC / IRBs, trial participants, trial registries, journals, regulators)	20
Consent or assent	#26a	Who will obtain informed consent or assent from potential trial participants or authorised surrogates, and how (see Item 32)	11
Consent or assent: ancillary studies	#26b	Additional consent provisions for collection and use of participant data and biological specimens in ancillary studies, if applicable	NA. These will be given in detail in the informed consent form.
Confidentiality	#27	How personal information about potential and enrolled participants will be collected, shared, and maintained in order to protect confidentiality before, during, and after the trial	20

For peer review only

Declaration of interests	#28	Financial and other competing interests for principal investigators for the overall trial and each study site	23
Data access	#29	Statement of who will have access to the final trial dataset, and disclosure of contractual agreements that limit such access for investigators	16-17
Ancillary and post trial care	#30	Provisions, if any, for ancillary and post-trial care, and for compensation to those who suffer harm from trial participation	NA. This is organizational level intervention providing psychosocial and is unlikely to result in serious harm to participants. KMU will provide post trial care in case of any serious harm.
Dissemination policy: trial results	#31a	Plans for investigators and sponsor to communicate trial results to participants, healthcare professionals, the public, and other relevant groups (eg, via publication, reporting in results databases, or other data sharing arrangements), including any publication restrictions	20
Dissemination policy: authorship	#31b	Authorship eligibility guidelines and any intended use of professional writers	NA (No professional writers sought)
Dissemination policy: reproducible research	#31c	Plans, if any, for granting public access to the full protocol, participant-level dataset, and statistical code	20
**Appendices**			
Informed consent materials	#32	Model consent form and other related documentation given to participants and authorised surrogates	Refer to appendices (supplementary file)
Biological specimens	#33	Plans for collection, laboratory evaluation, and storage of biological specimens for	NA

For peer review only

		genetic or molecular analysis in the current trial and for future use in ancillary studies, if applicable	

None The SPIRIT Explanation and Elaboration paper is distributed under the terms of the Creative Commons Attribution License CC-BY-NC. This checklist can be completed online using https://www.goodreports.org/, a tool made by the EQUATOR Network in collaboration with Penelope.ai

For peer review only
