# Supplementary material for: Traditional healers working with primary care and mental health for early intervention in psychosis in young persons: protocol for the feasibility cluster randomised controlled trial
Source: BMJ Open. 2023 Jul 14;13(7):e072471. doi: 10.1136/bmjopen-2023-072471 (PMC10351321; doi:10.1136/bmjopen-2023-072471)
Supplement: Reviewer comments [file bmjopen-2023-072471.reviewer_comments.pdf]

PEER REVIEW HISTORY

BMJ Open publishes all reviews undertaken for accepted manuscripts. Reviewers are asked to complete a checklist review form (**http://bmjopen.bmj.com/site/about/resources/checklist.pdf**) and are provided with free text boxes to elaborate on their assessment. These free text comments are reproduced below.

ARTICLE DETAILS

**TITLE (PROVISIONAL)**	Traditional healers working with primary care and mental health for early intervention in psychosis in young persons: Protocol for the feasibility cluster randomised controlled trial
**AUTHORS**	Farooq, Saeed; Sheikh, Saima; Dikomitis, Lisa; Ul-Haq, Mian; Khan, Abdul Jalil; Sanauddin, Noor; Malik, Wajid; Ali, Johar; Khan, Muhammad Firaz; Chaudhry, Imran; Husain, Nusrat; Gul, Muhammad; Irfan, Muhammad; Andrews, Gabrielle; Kaistha, Prachi; Shah, Syed Muhammad Uzair; Azeemi, Ishfaq; Hamid, Shumaila; Minhaz, Aaliya; Mallen, Christian; Lewis, Martyn

This article was not externally reviewed at BMJ Open. Protocols that have been independently assessed prior to submission to BMJ Open are usually fast-tracked to publication on the grounds that further substantial changes will not be possible. This independent assessment will usually be external, independent review for both a substantial grant award from a non-commercial or government funder and ethics approval.
